# Heavier congeners of CO and CO_2_ as ligands: from zero-valent germanium (‘germylone’) to isolable monomeric GeX and GeX_2_ complexes (X = S, Se, Te)†
†Electronic supplementary information (ESI) available: Experimental procedures and characterisation data for all new compounds, full details of the computational studies. Crystal data, details of data collections and refinements. CCDC 1457869–1457873. For ESI and crystallographic data in CIF or other electronic format see DOI: 10.1039/c6sc01839d


**DOI:** 10.1039/c6sc01839d

**Published:** 2016-05-10

**Authors:** Yun Xiong, Shenglai Yao, Miriam Karni, Arseni Kostenko, Alexander Burchert, Yitzhak Apeloig, Matthias Driess

**Affiliations:** a Department of Chemistry: Metalorganics and Inorganic Materials , Technische Universität Berlin , Strasse des 17. Juni 135, Sekr. C2 , D-10623 Berlin , Germany . Email: matthias.driess@tu-berlin.de; b Schulich Faculty of Chemistry and the Lise Meitner-Minerva Centre for Computational Quantum Chemistry , Technion-Israel Institute of Technology , Haifa 32000 , Israel . Email: apeloig@technion.ac.il

## Abstract

In contrast to molecular CO and CO_2_, their heavier mono- and dichalcogenide homologues, EX and EX_2_ (E = Si, Ge, Sn, Pb; X = O, S, Se, Te), are important support materials (*e.g.*, SiO_2_) and/or semiconductors (*e.g.*, SiS_2_) and exist typically as insoluble crystalline or amorphous polymers under normal conditions. Herein, we report the first successful synthesis and characterisation of an extraordinary series of isolable monomeric GeX and GeX_2_ complexes (X = S, Se, Te), representing novel classes of compounds and heavier congeners of CO and CO_2_. This could be achieved by solvent-dependent oxidation reactions of the new zero-valent germanium (‘germylone’)–GaCl_3_ precursor adduct (bis-NHC)Ge^0^→GaCl_3_**1** (bis-NHC = H_2_C[{NC(H)=C(H)N(Dipp)}C:]_2_, Dipp = 2,6-*i*Pr_2_C_6_H_3_) with elemental chalcogens, affording the donor–acceptor stabilised monomeric germanium(iv) dichalcogenide (bis-NHC)Ge^IV^(=X)=X→GaCl_3_ (X = S, **2**; X = Se, **3**) and germanium(ii) monochalcogenide complexes (bis-NHC)Ge^II^=X→GaCl_3_ (X = Se, **4**; X = Te, **5**), respectively. Moreover, the reactivity of **4** and **5** towards elemental sulphur, selenium, and tellurium has been investigated. In THF, the germanium(ii) monoselenide complex **4** reacts with activated elemental selenium to afford the desired germanium(iv) diselenide complex **3**. Unexpectedly, both reactions of **4** and **5** with elemental sulphur, however, lead to the formation of germanium(iv) disulfide complex **2** under liberation of elemental Se and Te as a result of further oxidation of the germanium centre and replacement of the Se and Te atoms by sulphur atoms. All novel compounds **1–5** have been fully characterised, including single-crystal X-ray diffraction analyses, and studied by DFT calculations.

## Abstract 2

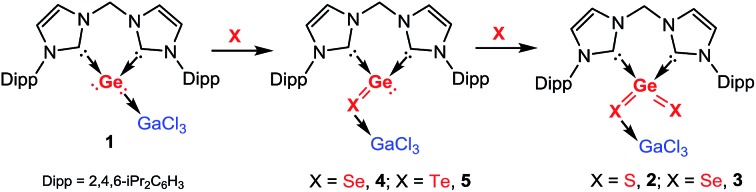
The first isolable germanium chalcogenide complexes **2–5** representing heavier congeners of CO and CO_2_ were synthesised from the germylone adduct **1**.

## Introduction

The binary group 14–16 compounds, EX and EX_2_ (E = Si, Ge, Sn, Pb; X = O, S, Se, Te), are important semiconducting materials and have been widely used in the manufacture of optical and electronic devices.^1^ Unlike their parent homologues CO and CO_2_, which feature a monomeric structure and are gaseous at ambient conditions, the latter chalcogenides are insoluble crystalline or amorphous polymers under the same conditions. Generally, they adopt polymeric structures owing to the relatively weak p_π_–p_π_ bond between germanium and the respective chalcogen atom and the high polarity of the E–X bond. It has been shown that the molecular variants of EX and EX_2_ can exist in condensed cryogenic matrices at very low temperature or diluted in the gas phase at high temperature. Thus they have solely been detected spectroscopically^2^ under extreme conditions or proposed as reactive intermediates^3^ and studied by theoretical calculations.^4^

**Chart 1:**
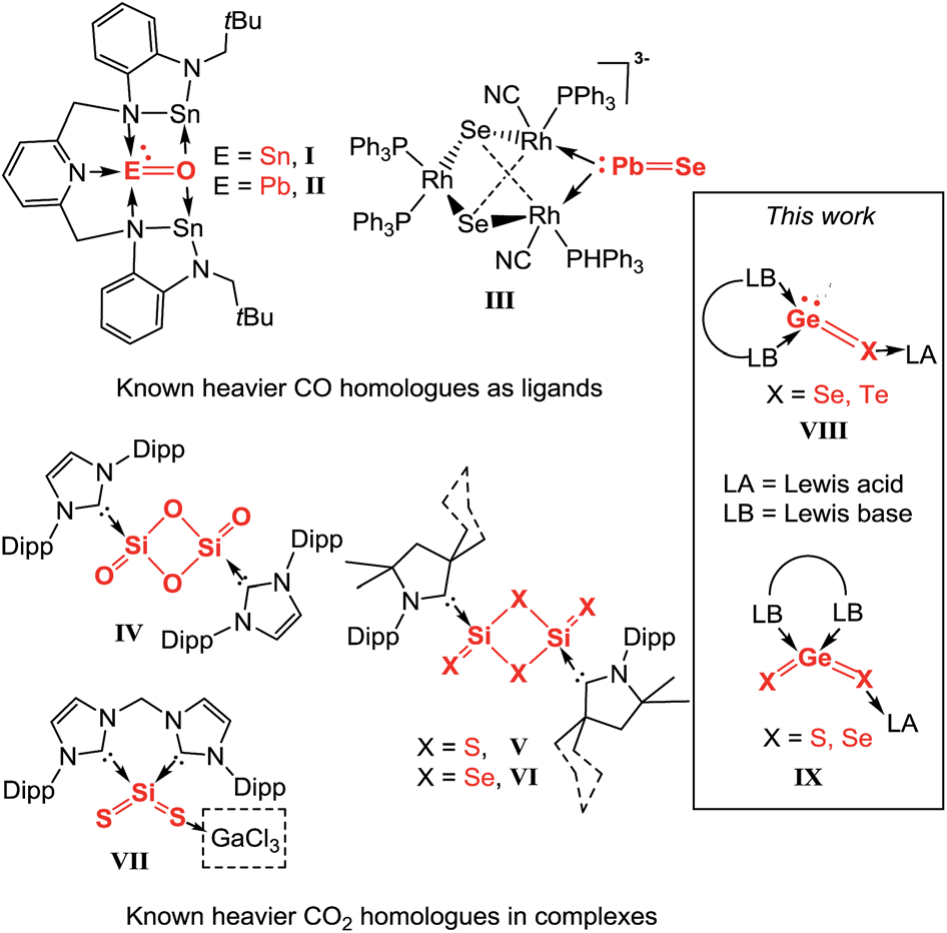
Known complexes **I–VII** of EX or EX_2_ (E = Si, Sn, Pb; X = O, S, Se, Te), respectively, and the novel :GeX and GeX_2_ complexes **VIII** and **IX** reported in this work.

During the last decades, the concept of kinetic and/or thermodynamic stabilisation has enabled great achievements in synthesising isolable low-coordinate group 14 element species as ligands in complexes. Several unusual compounds featuring elusive terminal E=O (E = Si, Ge, Sn, Pb)^5^ and E=X (X = S, Se, Te)^6^ double bonds could be synthesised and structurally characterised. However, complexes containing EX and EX_2_ are still very rare. The first examples include the Sn=O (**I**) and Pb=O (**II**) units stabilised by a benzannulated bis-stannylene reported by Hahn and co-workers (Chart 1).5*i* Very recently, Dehnen *et al.* published the first Pb=Se complex in {[K(18-crown-6)]-[K(en)_2_]K[Rh_3_(CN)_2_(PPh_3_)_4_(μ_3_-Se)_2_(μ-PbSe)]}_2_·3en (en = ethane-1,2-diamine) (**III**) as another type of EX coordinating to Rh sites (Chart 1).6*g* In the case of EX_2_, progress has also been made after the successful synthesis of Lewis base stabilised Si(0) complexes as precursors. By employing an *N*-heterocyclic carbene (NHC) (NHC = [(Dipp)NC(H)=C(H)N(Dipp)]C:, Dipp = 2,6-*i*Pr_2_C_6_H_3_), the disilicon(0) complex (NHC)Si=Si(NHC) was synthesised by Robinson and co-workers,7*a* which served as precursor to form the NHC-stabilised Si_2_O_4_**IV** (Chart 1), a complex of dimeric SiO_2_.7*b* In addition, Roesky *et al.* reported the CAAC (CAAC = cyclic alkyl amino carbene) stabilised disilicon(0) complex (CAAC)Si=Si(CAAC),8*a* from which the corresponding dimeric SiS_2_ (**V**) and SiSe_2_ (**VI**) complexes could be obtained (Chart 1).8b,c In the meantime, by employing the chelating bis-NHC ligand (bis-NHC = H_2_C[{–NC(H)=C(H)N(Dipp)}C:]_2_) we succeeded in the synthesis of a cyclic zero-valent monosilicon complex (‘silylone’)^9^ and its germanium homologue (‘germylone’),^10^ (bis-NHC)E(0) (E = Si, Ge). Recently, starting from the latter silylone, we could synthesize monomeric silicon disulphide complexes stabilised by bis-NHC and GaCl_3_, namely (bis-NHC)SiS_2_ and (bis-NHC)Si(=S)SGaCl_3_ (**VII**; Chart 1).^11^ In fact, the latter complexes represent the first donor–acceptor stabilised monomeric silicon analogues of CS_2_. To the best of our knowledge, no example of a molecular compound containing a divalent GeX or tetravalent GeX_2_ moiety as a ligand has been reported as yet. With the aforementioned bis-NHC supported germylone in hand, we set out to explore its reactivity towards elemental chalcogens with the aim to synthesise isolable :Ge=X (X = Se, Te **VIII**) and X=Ge=X (X = S, Se **IX**) complexes. Herein, we wish to present a series of unprecedented germanium analogues of both CO and CO_2_ utilizing the donor–acceptor stabilisation strategy.

## Results and discussion

**Scheme 1:**
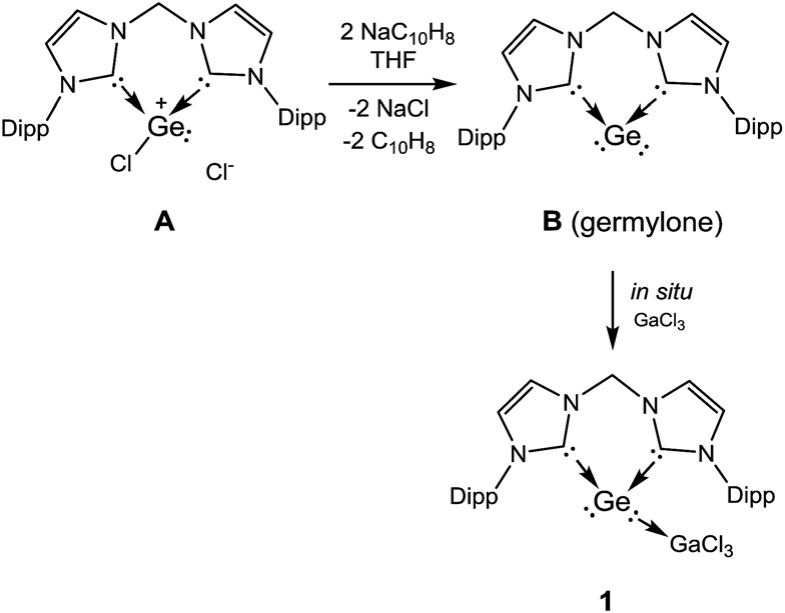
Synthesis of the germylone→gallium trichloride adduct **1** from the bis-NHC supported chlorogermyliumylidene chloride **A***via* the germylone **B**.

Shortly after communicating the bis-NHC supported germanium(0) species (germylone **B**, Scheme 1),^10^ we realized that **B** is sensitive not only towards air and moisture, but also to visible light. For its reactivity investigation, we introduced the Lewis acid GaCl_3_ to prepare the more stable germylone–GaCl_3_ adduct **1** through a one-pot reaction, starting from the bis-NHC supported chlorogermyliumylidene chloride (bis-NHC)GeCl_2_**A** (Scheme 1).^10^ Accordingly, the germylone **B**, prepared by reduction of **A** with two molar equivalents of sodium naphthalenide in THF, reacts *in situ* with one molar equivalent of GaCl_3_ to furnish the desired complex **1** as a colourless precipitate in 65% yield after work-up (Scheme 1).

**Fig. 1:**
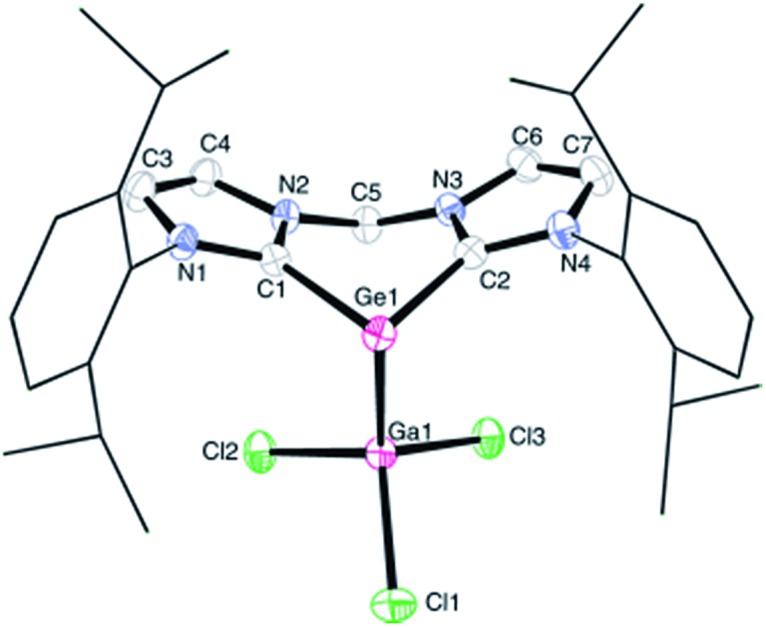
Molecular structure of **1**; thermal ellipsoids are drawn at the 50% probability level; all hydrogen atoms and four lattice solvent CH_3_CN molecules are omitted for clarity. Selected bond lengths (Å) and angles (°): Ge1–Ga1 2.520(1), Ge1–C1 2.043(3), Ge1–C2 2.033(3); C1–Ge1–C2 85.7(1), C1–Ge1–Ga1 90.8(1), C2–Ge1–Ga1 89.8(1).

The proton NMR spectrum of **1** reflects the coordination of the germanium centre *via* one of its lone pair of electrons to the GaCl_3_ moiety, thus lowering the symmetry of the molecule relative to that of its precursor germylone **B**. Therefore the spectrum of **1** exhibits four doublets for the methyl protons and two septets for the methine protons in the isopropyl groups. Moreover, the two geminal protons in the CH_2_ moiety on the backbone in **1** give two doublets with ^2^*J*_HH_ = 13.3 Hz (AB-spin system, ESI†). Complex **1** crystallised from acetonitrile solution in triclinic space group *P*1[combining macron] with four lattice CH_3_CN molecules in the asymmetric unit (Fig. 1). A single-crystal X-ray diffraction analysis of **1** revealed a three-coordinate germanium centre featuring pyramidal coordination geometry. The sum of the angles around the germanium atom amounts to 266.3°, implying that the vertex of the pyramid is occupied by one pair of electrons. Owing to the coordination of GaCl_3_, the average Ge–C bond length of 2.038(3) Å in **1** is longer than that in germylone **B** (Ge–C 1.965(2) Å).^10^ On the other hand, the Ge1–Ga1 distance of 2.520(1) Å is comparable to the Ge–Ga single bond (2.516(1) Å) supported by bulky substituents in [(Dipp)N–CH=CH–N(Dipp)]Ga–Ge[N(Dipp)]_2_CN(*i*Pr)_2_.12*a*

**Scheme 2:**
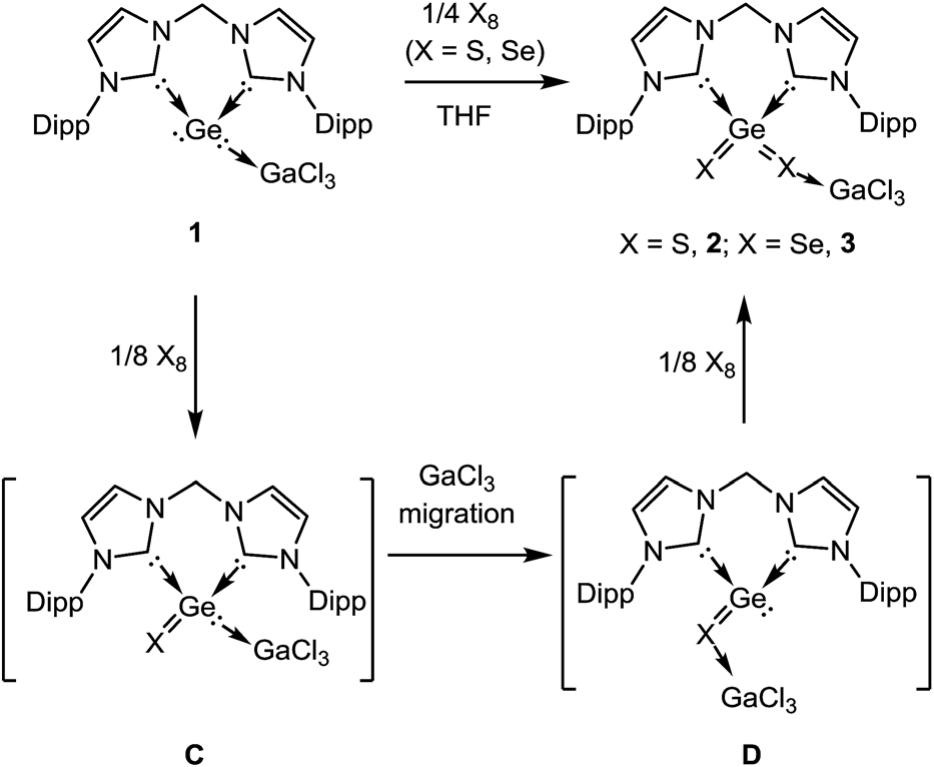
Synthesis of the bis-NHC and GaCl_3_ stabilised monomeric GeS_2_**2** and GeSe_2_**3** from **1**.

Complex **1** is much more stable than its precursor **B** because the zero valent germanium center is coordinated to the Lewis-acid GeCl_3_ by donating one of the two lone pairs of electrons to the electron-deficient gallium atom. It is noteworthy that germylone **B** also reacts readily with other Lewis acids such as AlBr_3_, BCl_3_, but the desired products could not be isolated in pure form as yet. However, **B** reacts smoothly with GaCl_3_ to afford **1** as an isolable product. Compound **1** is soluble in THF and acetonitrile. Treatment of THF solutions of **1** with 1/4 molar equivalents of X_8_ (X = S, Se) leads to quantitative formation of **2** and **3**, which could be isolated in excellent yields (90% for **2**, 88% for **3**) as colourless and pale yellow solids, respectively (Scheme 2). We note in passing that all attempts to synthesize **2** or **3** by dissolution of polymeric GeX_2_ (X = S, Se) in THF or acetonitrile solutions of the respective bis(NHC) ligand in the presence of GaCl_3_ failed. Likewise, other alternative approaches to synthesize **2** or **3** from the respective bis(NHC)GeCl_4_ precursor and *in situ* prepared M_2_X salts (M = Li, Na; X = S, Se) in the presence of GaCl_3_ were also unsuccessful which highlights the benefit of the reported synthetic method to form isolable monomeric GeX_2_ complexes. Compounds **2** and **3** are insoluble in hydrocarbons and only scarcely soluble in polar organic solvents such as THF and CH_3_CN as shown by a series of ESI-MS experiments. Thus only their ^1^H NMR spectra could be recorded in solutions but their low solubility prevents VT-NMR spectroscopy at low temperature.

Fortunately, single-crystals of **2** and **3** suitable for X-ray diffraction analyses were obtained in dilute THF solutions. Unexpectedly, compounds **2** and **3** exhibit higher symmetry than **1**, that is, only two doublets for the methyl protons in the CH(C*H*_3_)_2_ groups, instead of four doublets as in **1**, could be detected in their ^1^H NMR spectra, suggesting the statistically equal position of the GaCl_3_ moiety between two sulphur or selenium atoms in solution, respectively (see ESI†). The ESI-MS of **2** and **3** show the GaCl_3_-free molecular ion peak at *m*/*z* = 607.19696 (calc. 607.19789 for [**2** – GaCl_3_ + H]^+^, corresponding to [(bis-NHC)GeS_2_ + H]^+^) and at *m*/*z* = 703.08749 (calc. 703.08680 for [**3** – GaCl_3_ + H]^+^, corresponding to [(bis-NHC)GeSe_2_ + H]^+^), respectively.

**Fig. 2:**
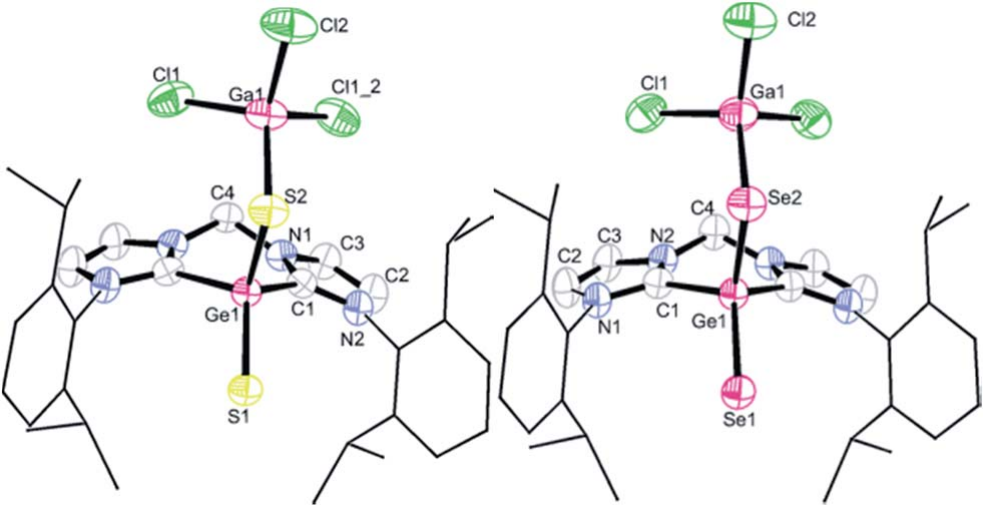
Molecular structures of **2** (left) and **3** (right). Thermal ellipsoids are drawn at the 50% probability level; all hydrogen atoms all hydrogen atoms and two lattice solvent THF molecules are omitted for clarity. Selected bond lengths (Å) and angles (°) for **2**: Ge1–S1 2.087(1), Ge1–S2 2.198(1), Ge1–C1 1.998(3), S2–Ga1 2.249(1); C1–Ge1–C2 91.4(2), S1–Ge1–S2 115.3(1), C1–Ge1–S1 113.0(1), C1–Ge1–S2 110.9(1), Ge1–S2–Ga1 107.9(1). Selected bond lengths (Å) and angles (°) for **3**: Ge1–Se1 2.214(1), Ge1–Se2 2.326(1), Ge1–C1 1.987(5), Se2–Ga1 2.374(1); C1–Ge1–C2 91.8(3), Se1–Ge1–Se2 115.2(1), C1–Ge1–Se1 113.0(1), C1–Ge1–Se2 110.8(1), Ge1–Se2–Ga1 104.5(1).

Compounds **2** and **3** crystallise isotypic in the monoclinic space group *Cm* with two lattice THF molecules in the respective asymmetric unit and the single-crystal X-ray diffraction analyses revealed them to be isostructural, with each of the germanium centres bound to two chalcogen atoms (Fig. 2). Thus the germanium centres in both compounds are four-coordinate and adopt a tetrahedral geometry with almost identical bond angles around the germanium atoms. The S1–Ge1–S2 angle of 115.3(1)° in **2** and Se1–Ge1–Se2 angle of 115.2(1)° in **3**, respectively, are reminiscent of the corresponding S–Si–S value observed in (bis-NHC)Si(=S)=S→GaCl_3_ (115.0(1)°).^11^ The C1–Ge1–C1′ angles of 91.4(2)° in **2** and 91.8(3)° in **3** are larger than that in **1** (85.7(1)°): accordingly, the Ge–C bond distances (1.998(3) Å in **2** and 1.987(5) Å in **3**) are slightly shorter than that in **1** (2.038 Å).

The two Ge–S distances in **2** (2.087(1) *vs.* 2.198(1) Å) and the two Ge–Se bonds in **3** (2.214(1) *vs.* 2.326(1) Å) show a significant difference. The Ge1–S1 bond length of 2.087(1) Å in **2** is slightly longer than that in Tbt(Tip)Ge=S (2.049 Å),12*b* but comparable to the Ge=S double bonds in LGe(=S)SH (2.064(1) Å)12*c* and LGe(=S)–SPh 2.071(1) Å (L = monovalent chelating organic groups),12*d* whereas the Ge1–S2 bond of 2.198(1) Å is slightly shorter than the Ge–S single bonds in the latter species (2.242(1) Å,12*c* 2.240(1) Å (ref. 12*d*)). Similarly, the Ge1–Se1 distance of 2.214(1) Å in **3** is slightly longer than that in Tbt(Tip)Ge=Se (2.180 Å, Tbt = 2,4,6-tris[bis(trimethylsilyl)methyl]phenyl, Tip = 2,4,6-triisopropylphenyl),12*b* but close to the doubly bonded Ge=Se group in LGe(=Se)–SePh (2.205(1) Å, L = aminotroponiminato ligand),12*d* while the Ge1–Se2 of 2.326(1) Å in **3** is only slightly shorter than the Ge–Se single bond in the latter complex (2.367(1) Å).12*d* The relatively large differences in the two Ge–X lengths (X = S, Se) in **2** and **3** can be understood in light of the calculations presented below.

Although the mechanism of formation of **2** and **3** is still unknown, it is reasonable to propose that the dichalcogenide formation occurs stepwise *via* the respective monochalcogenides as shown in Scheme 2. The initial step of the reaction implies the oxidation of the zero-valent germanium centre in **1** by one chalcogen atom to yield the divalent GeX complex **C**. The subsequent GaCl_3_ migration from the Ge(ii) centre to the more Lewis-basic chalcogenide site affords **D** bearing a three-coordinate Ge(ii) centre. Subsequent oxidation of the latter from Ge(ii) to Ge(iv) by an additional chalcogen atom results in the final product **2** or **3**, respectively. These suggestions are supported by results of DFT calculations.^13^ The calculated free energies (at room temperature) for the reaction **1** → **2** (and **3**) are shown in Fig. S7 in the ESI.† The migration of the GaCl_3_ fragment, **C** → **D** (Scheme 2, Fig. S7†), is exothermic, in the range of 11.8 to 15.8 kcal mol^–1^, for both X = S and Se, in the gas phase, in acetonitrile and in THF. However, the migration of GaCl_3_ is less exothermic for X = Se than for X = S by 3 kcal mol^–1^ in CH_3_CN. The free energy barriers for the two-step GaCl_3_ migration (**C** → **D**) is 29.9 and 29.5 kcal mol^–1^ for X = S and X = Se, respectively, in the gas phase (see details in the ESI†). The reaction leading from **D** (X = S) to **2** is by 5.0 kcal mol^–1^ more exothermic than for **D** (X = Se) to **3** (gas phase). The overall reaction from **1** to **2** (X = S) is more exothermic than from **1** to **3** (X = Se) by 10.3 (gas phase) 11.3 (CH_3_CN), and 10.9 (THF) kcal mol^–1^, in line with the experimental observation of a faster reaction for X = S than for X = Se.^14^

**Scheme 3:**
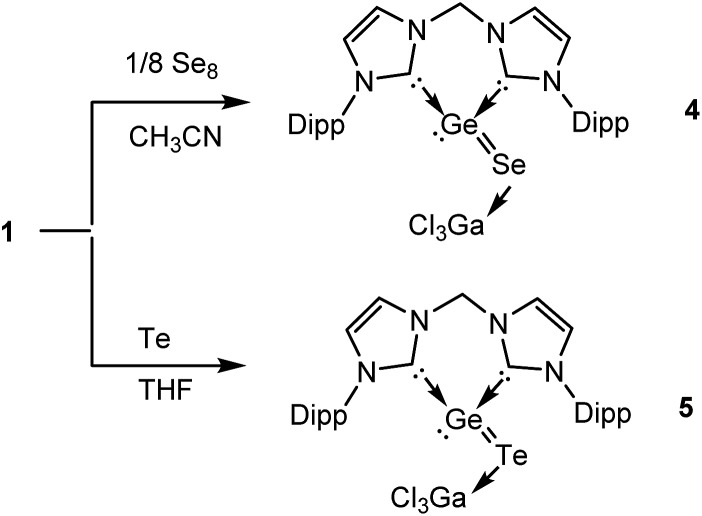
Formation of bis-NHC and GaCl_3_ stabilised GeSe and GeTe **4** and **5**, respectively, starting from **1**.

In order to isolate the proposed divalent germanium intermediates **C** and/or **D**, 1/8 equivalent of S_8_ and Se_8_ were employed for the reaction with **1** at –30 °C in THF solutions. However, even at low temperature, regardless of the ratio of the two reactants, the dichalcogenides **2** and **3** are the exclusive products. In contrast, by employing acetonitrile as solvent the reaction of **1** with 1/8 equivalents of S_8_ at room temperature became much slower than in THF, and the reaction afforded a mixture containing **2** and, presumably, **C** and/or **D** (X = S). Furthermore, the reaction of **1** with 1/8 molar equivalents of activated selenium Se_8_ at room temperature became so slow that the formation of the germanium(ii) monoselenide complex **4**, the selenium version of **D** (Scheme 2 and 3) was realized. In fact, after 3 days, **4** could be isolated from the resulting solution as a colourless solid in moderate yield (64%). For the reaction of **1** with elemental tellurium, the reaction is even slower than that with selenium and furnishes in THF at room temperature only the germanium(ii) monotelluride complex **5** (Scheme 3).

Compound **4** is better soluble in THF and CH_3_CN than the diselenide complex **3**. This allowed us to record its ^1^H- and ^13^C-NMR spectra in CD_3_CN at room temperature. The four doublets for the methyl protons and two septets for the methine protons in the CHMe_2_ groups indicate similar molecular symmetry to that of **1**. Again a similar “roof” effect was observed for the geminal protons of the bridging N–C*H*_2_–N group (^2^*J*_HH_ = 13.4 Hz) in the ^1^H NMR spectrum of **4** (AB-spin system). The ESI-MS spectrum of **4** (positive mode) shows an ion peak of [M – SeGaCl_3_ + Cl]^+^ at *m*/*z* = 577.21454 (calc. 577.21478) corresponding to the [(bis-NHC)GeCl]^+^ (**A^+^**) cation. Indeed, the facile formation of similar species with a [LECl]^+^ cation (L = 1,8-bis(tributylphosphazenyl)naphthalene, E = Si, Ge) have been described in our previous reports.^15^

Akin to **4**, the ^1^H NMR spectrum of **5** exhibits four doublets for the methyl protons and two septets for the methine protons in the CHMe_2_ groups. Similarly, the bridging N–C*H*_2_–N protons are coupled with each other with ^2^*J*_HH_ = 13.1 Hz (AB-spin system). Similar to that of **4**, in the ESI-MS spectrum of **5** a signal at *m*/*z* = 577.21399 (calc. 577.21478 for [M – Te – GaCl_3_ + Cl]^+^ corresponding to **A^+^** was observed.

**Fig. 3:**
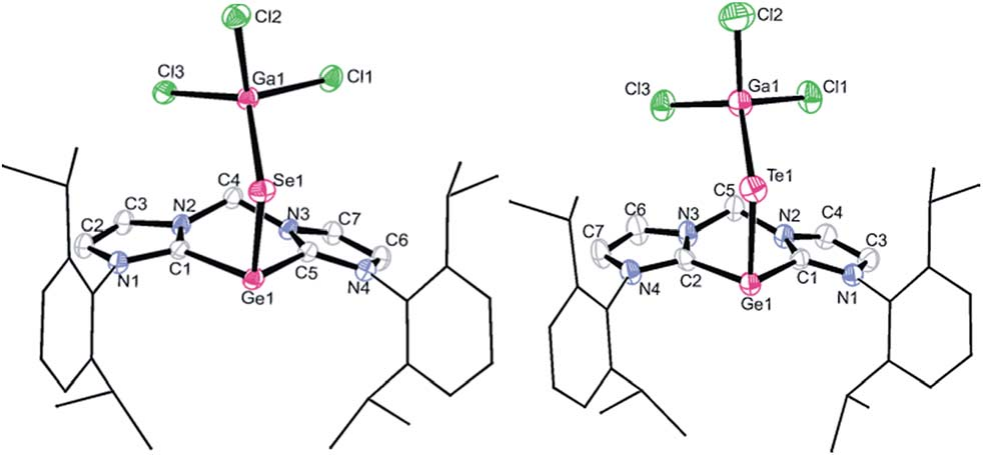
Molecular structures of **4** (left, all hydrogen atoms and one lattice solvent CH_3_CN molecule are omitted for clarity) and **5** (right, all hydrogen atoms and three lattice solvent THF molecules are omitted for clarity). Thermal ellipsoids are drawn at the 50% probability level. Selected bond lengths (Å) and angles (°) for **4**: Ge1–Se1 2.438(1), Ge1–C1 2.047(2), Ge1–C5 2.052(3), Se1–Ga1 2.337(1); C1–Ge1–C5 86.1(1), Se1–Ge1–C1 99.6(1), Se1–Ge1–C5 103.8(1), Ge1–Se1–Ga1 110.7(1). Selected bond lengths (Å) and angles (°) for **5**: Ge1–Te1 2.654(1), Ge1–C1 2.040(4), Ge1–C2 2.034(5), Te1–Ga1 2.526(1); C1–Ge1–C2 86.4(2), Te1–Ge1–C2 97.7(1), Te1–Ge1–C1 101.7(1), Ge1–Te1–Ga1 109.0(1).

Compound **4** crystallises in acetonitrile solutions in the monoclinic space group *P*2_1_/*c* with one lattice CH_3_CN molecule in the asymmetric unit (Fig. 3, left). The single-crystal X-ray diffraction analysis confirmed the proposed structure in which the germanium(ii) centre is stabilised by the chelating bis-NHC ligand and the Ge=Se moiety is supported by GaCl_3_ coordination, leading to a Ge–Se–Ga angle of 110.7(1)°. The three-coordinate Ge(ii) centre features a pyramidal coordination geometry with a sum of angle of 289.5°. The average Ge–C distance of 2.050(3) Å in **4** is very close to that observed in **1** (2.038(3) Å). The Se1–Ga1 length of 2.337(1) Å is comparable to that in **3** (2.374(1) Å) with a similar coordination environment. The Ge1–Se1 distance (2.438(1) Å) is significantly longer than the two Ge–Se bonds in the four-coordinate germanium diselenide complex **3** (2.214(1) and 2.326(1) Å). It is also longer than the Se–Ge length of 2.346 Å in the (CO)_5_W=Ge(SeAr)_2_ (Ar = 2,4,6-triisopropylphenyl) germanium(ii) species with a three-coordinate planar Ge centre.^16^ Thus a Ge=Se double bond character can not be concluded from the Ge–Se distance. This is also manifested in the resonance structures **4′′** calculated by NRT which are discussed below.

Single crystals of **5** suitable for X-ray diffraction analysis were obtained from THF solutions. **5** crystallises in the triclinic space group *P*1[combining macron] with three lattice THF molecules in the asymmetric unit. The latter analysis revealed a similar structure to that of **4** (Fig. 3, right). The germanium monotelluride moiety is coordinated by the bis-NHC and GaCl_3_ ligands, leading to a Ge–Te–Ga angle of 109.0(1)°. The germanium centre also adopts a trigonal–pyramidal coordination geometry with a sum of bond angle of 285.8° around the germanium atom. The average Ge–C bond length of 2.037(4) Å in **5** is close to those in its precursor **1** (2.038(3) Å) and in **4** (2.050(2) Å). The Ge1–Te1 distance of 2.654(1) Å is significantly longer than that in a four-coordinate germanium(iv) species LGe(R)=Te–GeCl_2_ (2.461(7) Å) (L = monovalent chelating organic group, R = monovalent organic group).^17^ Akin to the situation for the selenide **4**, the Ge–Te double bond character in **5** is rather low. It is noteworthy that the ^125^Te NMR resonance of **5**, similar to the ^77^Se NMR resonances of **3** and **4**, could not be observed, presumably owing to the zwitterionic nature of **3** and **4** (Scheme 5 and 6 and Fig. S6 in ESI†) which result in the broadening of the ^125^Te NMR resonances (for calculated chemical shifts see Table S12 in ESI†). Similar signal broadening occurred in the dimeric SiSe_2_ species stabilised by CAAC.8*c*

**Scheme 4:**
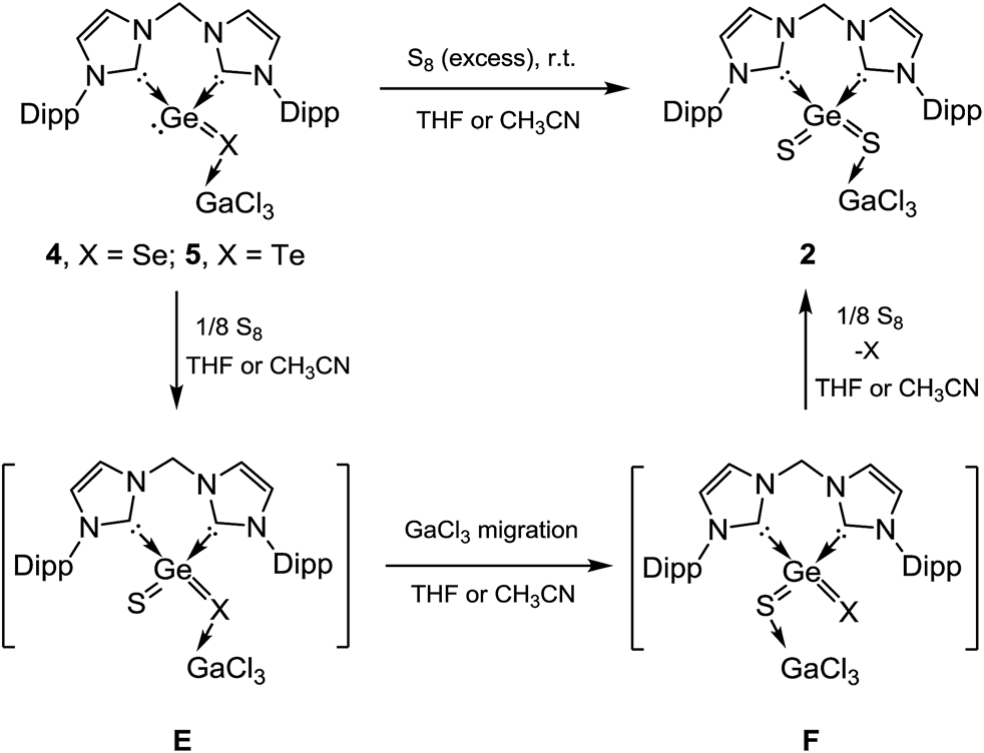
Reactivity of **4** and **5** toward elemental sulphur.

Since the germanium centres in **4** and **5** still feature one lone pair of electrons, their reactivity toward elemental sulphur, selenium, and tellurium has been investigated. Surprisingly, both **4** and **5** react with elemental sulphur to yield **2** immediately either in THF or in CH_3_CN, as shown by the ^1^H NMR spectra and by the precipitation of elemental orange Se and black Te, respectively (Scheme 4). Although the intermediates **E** and **F** could not be detected, they might play a role in the latter reactions. Accordingly, intermediates **E**, resulting from oxidation of **4** or **5** by sulphur, might undergo GaCl_3_ migration from X (X = Se, Te) to sulphur, spontaneously yielding **F**. Subsequent displacement reaction of the latter should afford **2** as the final product. The latter scenario is supported by DFT calculations. For instance, the calculated reaction free energy (at B3LYP-D3(BJ)/def2-SV using the PCM model for the solvents' effect) of **4** + 1/4 S_8_ → **2** + 1/8 Se_8_ is –21.6 (gas phase), –25.2 (acetonitrile) and –22.6 (THF) kcal mol^–1^. The migration from **E** to **F** is nearly thermo-neutral (Δ*G* = –0.74 (gas phase), –1.60 (acetonitrile), and –0.64 (THF) kcal mol^–1^).^14^

As expected, the reaction of **4** with activated selenium can afford compound **3** in CH_3_CN. However, after 24 h only 10% of **4** reacted. In contrast, in THF the reaction is complete after 2 h. This may explain why **4** could be isolated in CH_3_CN, whereas only **3** was isolable in THF. On the other hand, no reaction of **5** with elemental selenium in both THF and CH_3_CN could be detected after 24 h, confirming that **5** is less reactive than **4**. Furthermore, the reactivity of **4** towards tellurium in THF at room temperature was probed, however, after three days, no reaction occurred.

To obtain better understanding of the electronic properties and reactions of the novel compounds described in this article we performed DFT calculations for the synthesised compounds **1–5** and for the respective model compounds **1′–5′** where the bulky Dipp groups are replaced by Ph groups.^13^ In general the calculated optimized geometries of **1–5** at B3LYP-D3(BJ)/def2-TZVPP and those of **1′–5′** optimized at B3LYP-D3(BJ)/def2-SV are in good agreement with the corresponding X-ray structures.^18^ A selection of calculated geometry parameters are presented in Table 1 (more details are provided in Table S11 in ESI†). Unless otherwise stated we discuss the calculated results obtained at the B3LYP-D3(BJ)/def2-SV//B3LYP-D3(BJ)/def2-SV level of theory.

**Table 1:** Experimental representative distances *r* (Å) in **1–5** (R = Dipp) and the calculated^*a*^ values of *r* and the corresponding Wiberg Bond Indices (WBI) in **1′–5′** (R = Ph)

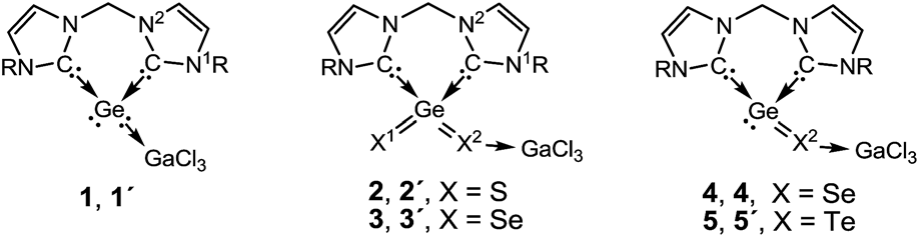						
Compound	Bonds					
	Ge–C:	Ge–X1	Ge–X2	:C–N^1^	:C–N^2^	X^2^–Ga
**1**, **1′**						
*r* (exp.)	2.043	—	—	1.349	1.354	—
*r* (calc.)^*b*^	2.045	—	—	1.356	1.360	—
WBI	0.74	—	—	1.23	1.21	—

**2**, **2′**						
*r* (exp.)	1.998	2.087	2.198	1.329	1.358	2.249
*r* (calc.)	2.022	2.091	2.213	1.344	1.354	2.280
WBI	0.59	1.39	0.92	1.28	1.24	0.71

**3**, **3′**						
*r* (exp.)	1.987	2.214	2.326	1.352	1.366	2.374
*r* (calc.)	2.020	2.231	2.348	1.344	1.354	2.404
WBI	0.60	1.36	0.94	1.28	1.24	0.75

**4**, **4′**						
*r* (exp.)	2.047	—	2.438	1.346	1.353	2.337
*r* (calc.)	2.071	—	2.435	1.352	1.358	2.381
WBI	0.67	—	0.89	1.26	1.23	0.79

**5**, **5′**						
*r* (exp.)	2.040	—	2.654	1.350	1.357	2.526
*r* (calc.)	2.058	—	2.654	1.352	1.359	2.582
WBI	0.69	—	0.92	1.25	1.23	0.85

^*a*^R = Ph, at B3LYP-D3(BJ)/def2-SV.

^*b*^r(Ge–Ga) = 2.549 Å, WBI = 0.73.

The calculated Ge1–X1 distances (**2′**, X = S, and **3′**, X = Se) of 2.091 Å and 2.231 Å are longer than those of Me_2_Ge=X (X = S: 2.045 Å, X = Se: 2.174 Å), respectively. This trend is in line with the Ge–X Wiberg Bond Indices (WBI)^19^ which in Me_2_Ge=X (X = S, 1.82 and X = Se, 1.84) are larger than those of **2′** (1.39) and **3′** (1.36). The calculated Ge=X bond length and WBIs in linear X=Ge=X are 2.016 Å and 1.79, respectively, for X = S, and 2.145 Å and 1.82, respectively, for X = Se. The significantly smaller WBIs in **2′** and **3′** and the longer bond distances (Table 1) reflect a partial contribution of a double bond character in their Ge–X1 bonds. The Ge–X2 bonds, 2.198 Å (exp.), 2.213 Å (calc.), WBI = 0.92 (X = S); 2.326 Å (exp.), 2.348 Å (calc.), WBI = 0.94 (X = Se), are slightly shorter and have somewhat larger WBIs than those calculated in Me_3_Ge–XH (2.265 Å, WBI = 0.88, X = S; 2.397 Å WBI = 0.91, X = Se). The NCN bonds are 3-centre 4-electron bonds. This is manifested in the resonance structures which involve these electrons, *i.e.*, N^1^=C:N^2^ ↔ N^1^:C=N^2^, and which are reflected in the C–N WBI of *ca.* 1.25, suggesting a partial double bond character. The Ge–C bond length is 1.998 Å (2.022 Å, WBI = 0.59, calc.) and 1.987 Å (2.020 Å, WBI = 0.60, calc.) in **2** and **3**, respectively. The small WBI may reflect the contribution of resonance structures in which the Ge is bound to only one carbene unit (see below). The Ge–C bonds are longer than that in the germylone precursor **B** (average r(Ge–C) = 1.963 Å, exp.^10^ 1.981 Å, WBI = 1.0.) and of the acyclic, germylones supported with two CAAC ligands, synthesised by Roesky *et al.*, of 1.940 Å and 1.954 Å.^20^

**Scheme 5:**
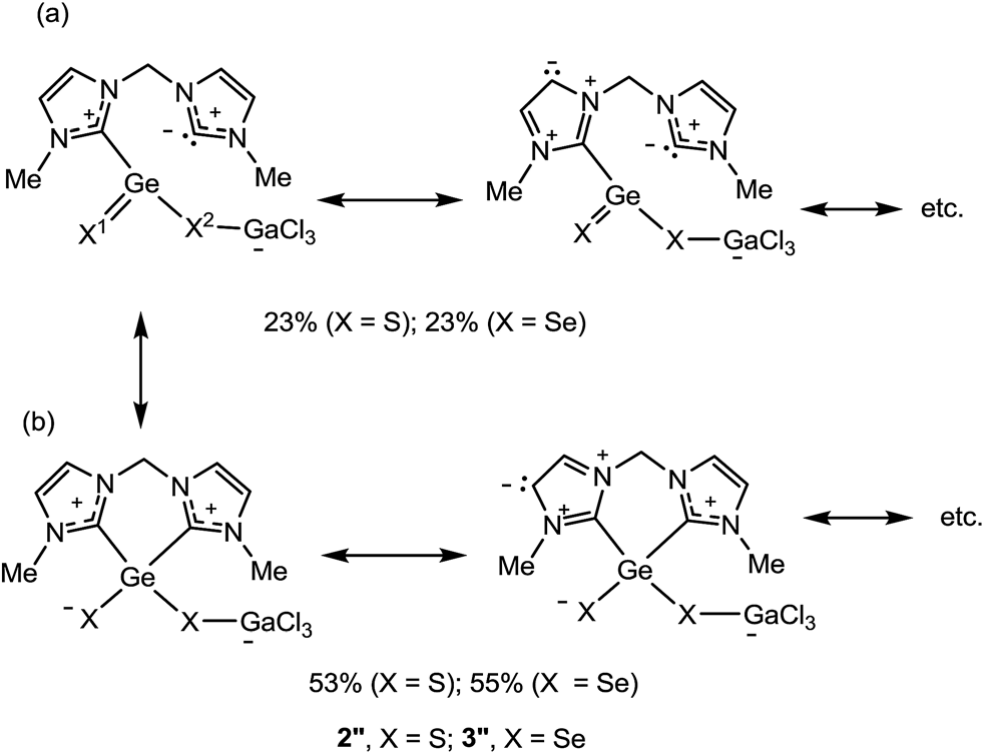
Predominant resonance structures of the model compounds **2′′** and **3′′** calculated by NRT. The relative abundance of the resonance structures exhibits the sum over all possible permutations within the NHC rings (above 1% weight, two are shown, see more in Scheme S1 in the ESI†).

The dichalcogenides **2** and **3** as well as the monochalcogenides **4** and **5** are highly delocalised compounds featuring many resonance structures. The most predominant resonance structures according to NRT calculations, for model compounds **2′′** and **3′′** (R = Me), feature two resonance structure types which are responsible for *ca.* 75% of the total contributions (Scheme 5): (a) structures containing a X^1^=Ge–X–GaCl_3_ subunit with a X^1^=Ge double bond and where the Ge is bound to only one of the carbenes, accounting for 23%, for both X = S and X = Se, of the total. (b) Structures containing a X–Ge–X–GaCl_3_ subunit with a bond between the Ge and each of the NHC units. These structures account for 53% (X = S) and 55% (X = Se) of the total. Many electron permutations in the NHC rings are possible for both resonance structure types and the values in Scheme 5 present their summation. These resonance structures indicate a partial double bond character of the Ge–X1 and of the endocyclic C–N bonds and a single bond character of the Ge–X2 bond. The corresponding NRT bond orders of Ge–X1 are 1.38, with a covalent contribution of 0.85 and an ionic contribution of 0.52 (X = S) and 1.36 with a covalent contribution of 0.94 and an ionic contribution of 0.41 (X = Se), reflecting the lower polarity of the Ge–Se bonds (see Fig. S6 in the ESI†). The NRT bond orders of Ge–X2 are 0.95 and 0.96 for X = S and X = Se, respectively. This bond orders are in good agreement with the calculated WBIs and explain the trends in the bond lengths (see above and in Table 1).

**Scheme 6:**
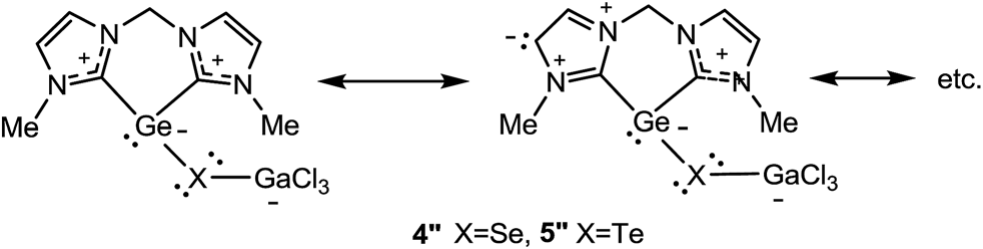
Calculated major resonance structures contributing to the structures of **4′′** and **5′′**.

The relatively long Ge–Se and Ge–Te distances in the monochalcogenides **4** and **5** (see discussion above and Table 1) led to the conclusion that there is no Ge=X double bond in **4** and **5**. This is supported by the NRT calculations for **4′′** and **5′′** R = Me which show that the major contribution stems from the resonance structures shown in Scheme 6 and their possible resonance permutations within the NHC fragments with no contribution from a Ge=X (X = Se, Te) double bond.

The calculated Natural Population Analysis (NPA) charge distribution in compounds **1′–5′** is shown in Fig. S6 in the ESI.† In all compounds the total charge on the bis-carbene moiety is positive (0.52 el.), indicating that the carbene units are donating electrons to the Ge^IV^X_2_GaCl_3_ and Ge^II^XGaCl_3_ fragments. The positive charge on the Ge atom decreases along the series X = S > Se > Te in **2′**, **3′**, **4′** and **5′**, while the charge on the GaCl_3_ fragment increases along this series. Our results indicate that the chalcogen–GaCl_3_ interaction is strong. In fact, attempts to remove the coordinated GaCl_3_ from **2–5** by using strong external Lewis bases such as ‘free’ NHC with methyl ligands at nitrogen or less substituted bis-NHCs or chelating bis-thiols had been performed, but no reaction could be observed. At elevated temperature decomposition occurred, leading to hitherto unidentified mixtures.

## Conclusions

Due to the substantial Lewis acid stabilisation of the Ge(0) atom in **1**, the Ge(0) atom can be readily oxidised with elemental chalcogens to form, in solvent-dependent reactions, the first donor–acceptor stabilised isolable monomeric germanium disulfide **2**, diselenide **3**, monoselenide **4**, and monotelluride complex **5**, respectively. They represent novel classes of heavier congeners of CO and CO_2_ complexes. Apparently, the presence of the GaCl_3_ Lewis acid is essential for the stabilisation of all monomeric species bearing highly polar Ge=X bonds. In THF, the germanium(ii) monoselenide complex **4** can further be oxidised with activated selenium to yield the corresponding germanium diselenide complex **3**. Unexpectedly, both selenide and telluride compounds **4** and **5** react with elemental sulphur to produce **2** with liberation of elemental selenium and tellurium, respectively. The unusual structural, spectroscopic, and electronic properties of these novel species could be determined and analysed by combined experimental and computational investigations. One of the important lessons from the calculations is that the bonding framework in all these compounds is complex and cannot be described properly by a single valance bond structure. Currently, we continue to explore the synthesis of Lewis acid-free germanium and silicon chalcogenide complexes and to exploit their reactivity in the context of small molecule activation and ligand ability in metal coordination chemistry. The strategy of donor–acceptor stabilisation is also expected to pave the way to isolable monomeric SiO and SiO_2_ complexes. Respective studies are currently in progress.

## Supplementary Material

Supplementary information

Crystal structure data

## References

[cit1] (b) Schwarzl T., Böberl M., Heiss W., Springholz G., Fürst J. and Pascher H., Proc. GMe Forum, 2003, p. 103.

[cit2] (b) Friesen M. and Schnöckel H., Organosilicon Chemistry IV: From Molecules to Materials, [Lectures and Poster Contributions presented at the Muechner Silicontage], 4th edn, Muenchen, April 2008, pp. 59–63.

[cit3] (a) Huber K. P. and Herzberg G., Molecular Structure and Molecular Spectra IV. Constants of Diatomic Molecules, Van Nostrand Reinhold, New York, 1979.

[cit4] Wu Z. J. (2003). Chem. Phys. Lett..

[cit5] Yao S., Xiong Y., Brym M., Driess M. (2007). J. Am. Chem. Soc..

[cit6] Tokitoh N., Matsumoto T., Manmaru K., Okazaki R. (1993). J. Am. Chem. Soc..

[cit7] Wang Y., Xie Y., Wie P., King R. B., Schaefer H. F., Schleyer P. v. R., Robinson G. H. (2008). Science.

[cit8] Mondal K. C., Samuel P. P., Roesky H. W., Aysin R. R., Leites L. A., Neudeck S., Lìbben J., Dittrich B., Holzmann N., Hermann M., Frenking G. (2014). J. Am. Chem. Soc..

[cit9] Xiong Y., Yao S., Inoue S., Epping J. D., Driess M. (2013). Angew. Chem., Int. Ed..

[cit10] Xiong Y., Yao S., Tan G., Inoue S., Driess M. (2013). J. Am. Chem. Soc..

[cit11] Xiong Y., Yao S., Müller R., Kaupp M., Driess M. (2015). Angew. Chem., Int. Ed..

[cit12] Green S. P., Jones C., Lippert K.-A., Mills D. P., Stasch A. (2006). Inorg. Chem..

[cit13] (a) Frisch M. J., et al., Gaussian 09, Revision D.01, Gaussian, Inc., Wallingford CT, 2013.

[cit14] (a) In the calculations we used S_8_ and Se_8_ the crown D_4d_ allotropes (for S_8_ see: M. W. Wong, Y. Steudel and R. Steudel, *Chem. Phys. Lett.*, 2002, **364**, 387; for Se_8_ see: T. Maaninen, J. Konu and R. S. Laitinen, *Acta Crystallogr., Sect. E: Struct. Rep. Online*, 2004, **60**, o2235) (b) These energies are not the actual reaction energies because we do not include the S_7_/S_6_ or Se_7_/Se_6_ fragments that may result in these reactions, as no information is available about their structures. The only reaction energies which reflect the actual experimental energies are the calculated migration energies and barriers

[cit15] Xiong Y., Yao S., Inoue S., Irran E., Driess M. (2012). Angew. Chem., Int. Ed..

[cit16] du Mont W.-W., Lange L., Pohl S., Saak W. (1990). Organometallics.

[cit17] Li B., Li Y., Zhao N., Chen Y., Chen Y., Fu G., Zhu H., Ding Y. (2014). Dalton Trans..

[cit18] Very similar geometries were calculated for **3**, **4** and **5** with ZORA-BP86-D3(BJ)/def2-TZVP(-f) relativistic basis set (see details in the ESI)

[cit19] Wiberg K. B. (1968). Tetrahedron.

[cit20] Li Y., Mondal K. C., Roesky H. W., Zhu H., Stollberg P., Herbst-Irmer R., Stalke D., Andrada D. M. (2013). J. Am. Chem. Soc..

