# Supplementary material for: Heavier congeners of CO and CO2 as ligands: from zero-valent germanium (‘germylone’) to isolable monomeric GeX and GeX2 complexes (X = S, Se, Te)
Source: Chem Sci. 2016 May 10;7(8):5462–9. doi: 10.1039/c6sc01839d (PMC6021755; doi:10.1039/c6sc01839d)
Supplement: Supplementary file 1 [file SC-007-C6SC01839D-s001.pdf]

Electronic Supplementary Material (ESI) for Chemical Science.
This journal is © The Royal Society of Chemistry 2016

Electronic Supplementary Information (ESI) for Chemical Science.
This journal is ©The Royal Society of Chemistry 2015

Supporting Information

Heavier congeners of CO and CO_2 as ligands: from zero-valent germanium ('germylone') to isolable monomeric GeX and GeX_2 complexes (X = S, Se, Te)

Yun Xiong,[a] Shenglai Yao,[a] Miriam Karni,[b] Arseni Kostenko,[b] Alexander Burchert,[a] Yitzhak Apeloig,*[b] Matthias Driess*[a]

a. Department of Chemistry: Metalorganics and Inorganic Materials, Technische Universität Berlin, Strasse des 17. Juni 135, Sekr. C2, D-10623 Berlin (Germany). E-mail: matthias.driess@tu-berlin.de
b. Schulich Faculty of Chemistry and the Lise Meitner-Minerva Centre for Computational Quantum Chemistry, Technion-Israel Institute of Technology, Haifa 32000 (Israel). E-mail: apeloig@technion.ac.il

Content

A Synthesis and Characterisation……………………………………2

B Crystallographic data…………………………………………….13

C. Theoretical Calculation…………………………………..…….27

D. References……………………………………..………..…52

A Synthesis and Characterisation

General Considerations

All experiments were carried out under dry oxygen-free nitrogen using standard Schlenk techniques. Solvents were dried by standard methods and freshly distilled prior to use. The NMR spectra were recorded with Bruker spectrometers ARX200 and AV400 referenced to residual solvent signals as internal standards (^{1}H, $^{13}C\{H\}$). Abbreviations: *s* = singlet; *d* = doublet; *t* = triplet; *sept* = septet; *m* = multiplet; *br* = broad. Solid state CP/MAS ^{77}Se and ^{125}Te NMR spectra were recorded with a Bruker AVANCE II 400 spectrometer and 4mm double resonance MAS (Magic angle spinning) probes. Elemental analyses and ESI-MS were performed by the analytical labor in the Institute of Chemistry, Technical University of Berlin, Germany. The precursors **A** is synthesized according to literature.[1]

Single-Crystal X-ray Structure Determination

Crystals were each mounted on a glass capillary in per-fluorinated oil and measured in a cold N_2 flow. The date of **1** - **5** were collected on an Oxford Diffraction Supernova, Single source at offset, Atlas at 150 K (Cu- Kα-radiation, λ = 1.5418 Å). The structures were solved by direct method and refined on F^2 with the SHELX-97 [2] software package. In the crystal of compound **1**, four lattice solvent CH_3CN molecules are observed; while in the crystals of **2** and **3** two lattice THF molecules are found. The crystal of compound **4** contains one CH_3CN molecule as lattice solvent. Compound **5** crystalizes with three lattice THF molecules in the asymmetric unit. One of the latter lattice THF molecule is disordered over two positions which have been refined with 88 restraints on displacement parameters. CCDC 1457869 (**1**), CCDC 1457873 (**2**), CCDC 1457870 (**3**), CCDC 1457872 (**4**), and CCDC 1457871 (**5**) contain the supplementary crystallographic data for this paper. These data can be obtained free of charge by contacting The Cambridge Crystallographic Data Centre, 12, Union Road, Cambridge CB2 1EZ, UK; fax: +44 1223 336033.

Synthesis and Characterisation of 1

To a suspension of bis-NHC stabilized germyliumylidene chloride **A** (6.60 g, 10.78 mmol) in THF (100 mL) was added a freshly prepared THF solution of $NaC_{10}H_8$ (two equivalent in 50 ml THF) at -20 °C under stirring. The reaction flask was wrapped with aluminum foil to keep the reaction mixture away from light. The reaction mixture was stirred for 4h to yield **B**. After the reduction is finished, $GaCl_3$ (1.90 g, 10.78 mmol) was added to the reaction solution containing **B**, and the cooling bath was taken away. The reaction mixture was stirred at room temperature for 2h, the volatiles were then removed under reduced pressure, and naphthalene was washed away with n-hexane (2 × 50ml). The product **1** was extracted with THF (3 × 100 ml). After dried under vacuum, compound **1** was obtained as a colorless powder with a yield of 5.02 g (7.00 mmol, 65%). The single crystals suitable for X-ray analysis was obtained from its solution in CH_3CN at 4 °C. M. p. 255 °C (decomp.); ^{1}H NMR (200.13 MHz, $[D_3]$ CH_3CN, 25°C): δ = 1.15 (*d*, $^{3}J(H,H)$ = 7.0 Hz, 12 H; -CH(CH_3)$_3$), 1.18 (*d*, $^{3}J(H,H)$ = 7.0 Hz, 6 H; -CH(CH_3)$_3$), 1.35 (*d*, $^{3}J(H,H)$ = 7.0

Hz, 6 H; -CH(C*H*$_3$)$_3$), 2.28 (sept., ^3J(H,H) = 7.0 Hz, 2 H; -C*H*(CH$_3$)$_3$), 3.22 (sept., ^3J(H,H) = 7.0 Hz, 2 H; -C*H*(CH$_3$)$_3$), 6.57, 6.74 (AB-spin system, ^2J = 13.3 Hz; 2 H; C*H*$_2$), 7.42 (*d*, ^3J(H,H) = 7.0 Hz, 2 H; *Ph*), 7.48 (*d*, ^3J(H,H) = 7.0 Hz, 2 H; *Ph*), 7.60 – 7.64 (*dd*, ^3J(H,H) = 7.7 Hz, 7.8 Hz, 2 H; *Ph*), 7.55 (*d*, ^3J(H,H) = 2.0 Hz, 2 H; =C*H* (imid)), 7.91 (*d*, ^3J(H,H) = 2.0 Hz, 2 H; =C*H* (imid)); ^{13}C{^{1}H} NMR (100.61 MHz, [D$_3$] CH$_3$CN, 25 ºC): δ = 23.30, 23.47, 24.02, 24.65 (CH(*C*H$_3$)$_3$), 27.62, 28.19 (*C*H(CH$_3$)$_3$), 62.92 (*C*H$_2$), 122.53 (=*C*H(imid)), 124.09 (*m-Ph*), 124.44 (*m-Ph*), 125.99 (=*C*H(imid)), 130.81 (*p-Ph*), 133.21(*Ph*), 145.11(*o-Ph*), 146.86 (*o-Ph*), 174.16 ppm (carbene-*C*); IR (CsI, cm^{-1}): 2967(vs), 2869(s), 1560(w), 1469(vs), 1411(s), 1387(w), 1364(w), 1292(m), 1256(w), 1210(m), 1187(s), 1116(s), 1060(s), 957(w), 938(w), 807(s), 760(vs), 686(m), 627(w), 562(w), 466(w), 393(w), 332(m), 309(s), 285(m); HR ESI-MS: *m*/*z*: 577.21467(calc. 577.21478 [M-GaCl$_3$+Cl]$^+$, depicted below, top: exp.; bottom: calc.). elemental analysis calcd. (%) for $C_{31}H_{40}N_4GeGaCl_3\cdot 3THF$: C 55.32, H 6.91, N 6.00; found: C 55.10, H 6.69, N 6.10.

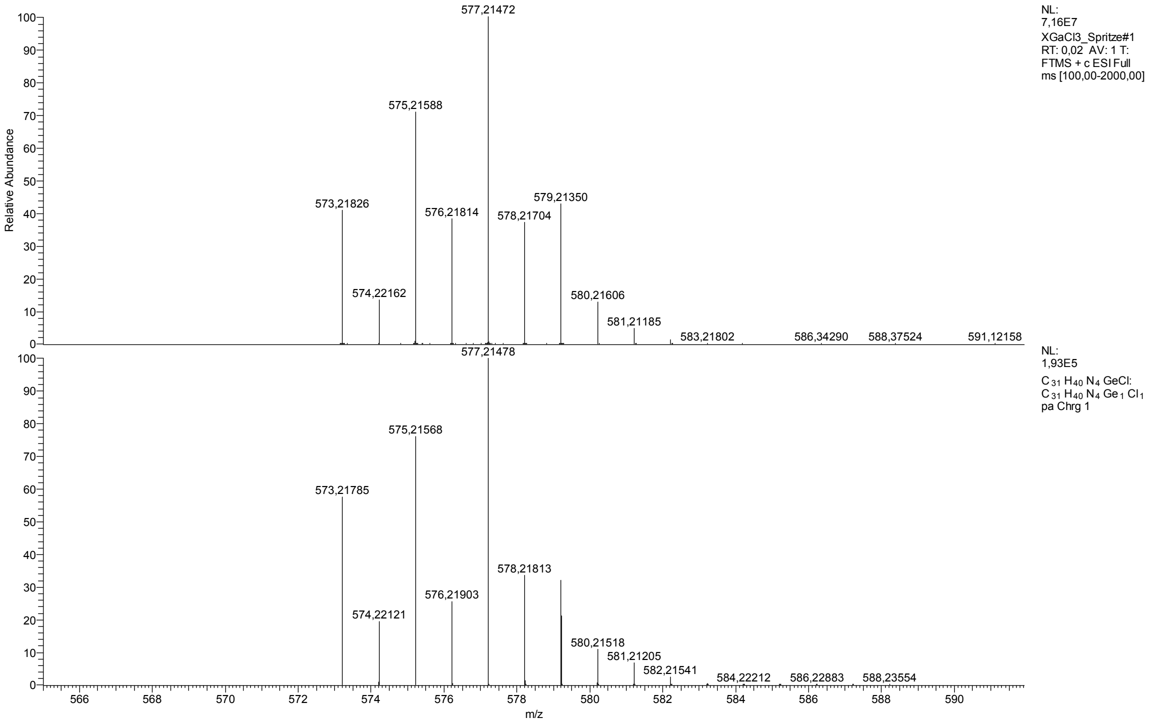

HR ESI-MS spectrum of **1**.

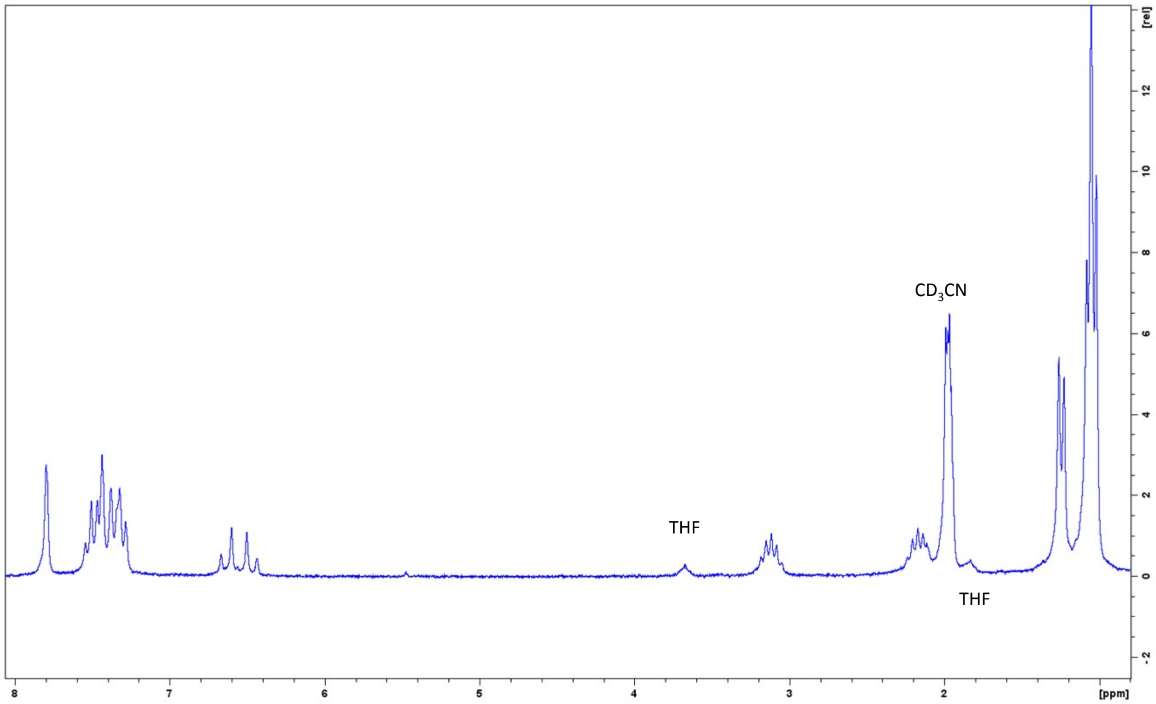

^{1}H NMR spectrum of **1**.

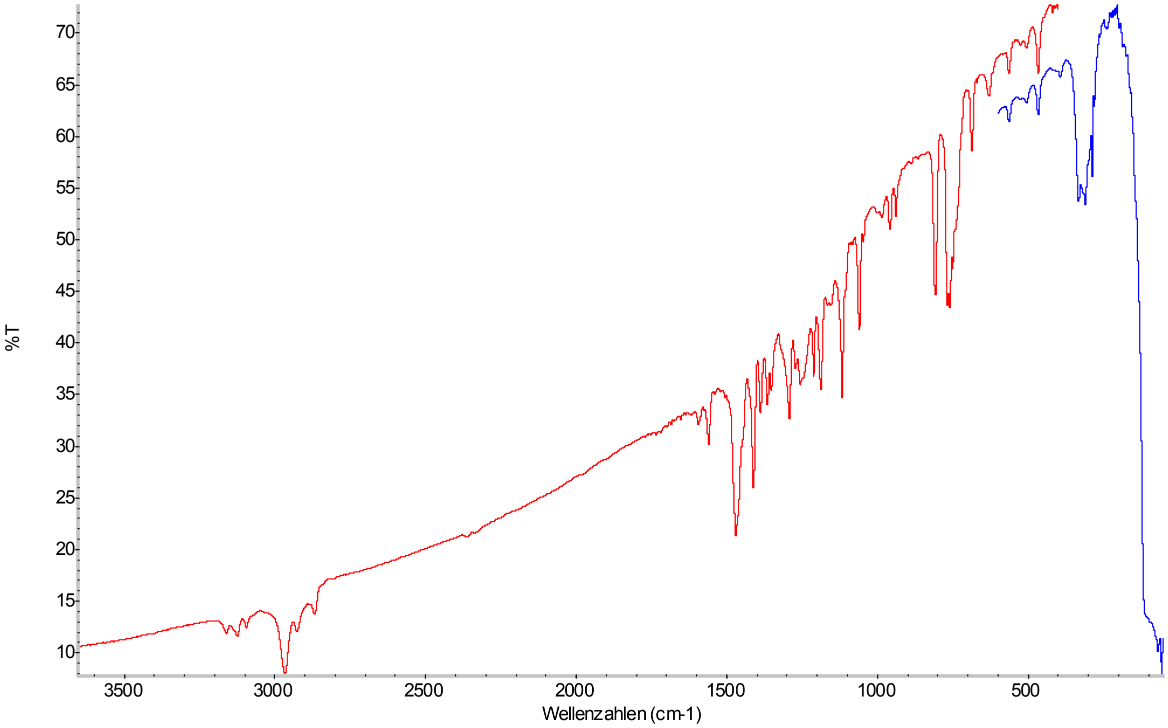

IR spectra (CsI) of **1**.

Synthesis and Characterisation of 2

To a solution of **1** (0.30 g, 0.42 mmol) in THF (20 mL) was added elemental sulfur (S_8, 0.027 g, 0.105 mmol) at -30 ^{o}C under stirring. The reaction mixture was allowed to warm to room temperature and stirred at that temperature for 2h. The volatiles were evaporated and the residue was washed with n-hexane (10 mL). After dried under vacuum compound **2** was obtained as a colorless powder with a yield of 0.30 g (0.38 mmol, 90%). M. p. 201 ºC (decomp.); ^{1}H NMR (200.13 MHz, [D_3] CH_3CN, 25ºC): δ = 1.04 (*d*, ^{3}J(H,H) = 7.0 Hz, 12 H; -CH(CH_3)$_3$), 1.31 (*d*, ^{3}J(H,H) = 7.0 Hz, 12 H; -CH(CH_3)$_3$), 2.95 – 3.20 (*m*, 4H; -C*H*(CH_3)$_3$), 6.80 – 7.00 (*m*, 2 H; CH_2), 7.30 (*d*, ^{3}J(H,H) = 7.0 Hz, 4 H; *m-Ph*), 7.50 (*dd*, ^{3}J(H,H) = 7.7 Hz, 7.8 Hz, 2 H; *p-Ph*), 7.59 (*d*, ^{3}J(H,H) = 2.0 Hz, 2 H; =C*H* (imid)), 7.88 (*d*, ^{3}J(H,H) = 2.0 Hz, 2 H; =C*H* (imid)); HR ESI-MS: *m*/*z*: 607.19696 (calc. 607.19789 [M-$GaCl_3$+H]$^{+}$, depicted below, top: exp.; bottom: calc.). IR (CsI, cm^{-1}): 2966 (vs), 2929(s), 2869(s), 1560(w), 1467(vs), 1420(w), 1386(m), 1365(m), 1307(m), 1257(w), 1212(w), 1195(m), 1131(m), 1061(m), 937(w), 808(s), 758(vs), 692(w), 631(w), 499(s), 416(m), 368(vs) ($\upsilon_{(Ge-S)}$); elemental analysis calcd. (%) for $C_{31}H_{40}N_4GeGaCl_3S_2$·THF: C 49.25, H 5.67, N 6.56, S 7.51; found: C 48.99, H 5.49, N 6.58, S 7.35.

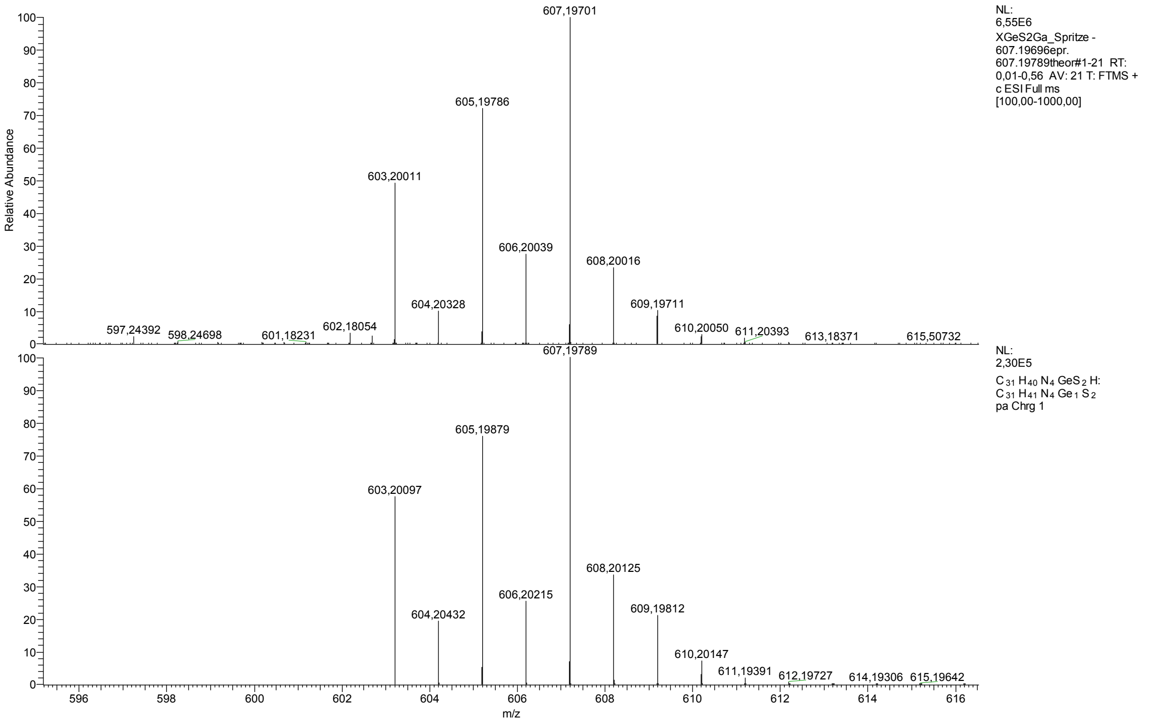

HR ESI-MS spectrum of **2**.

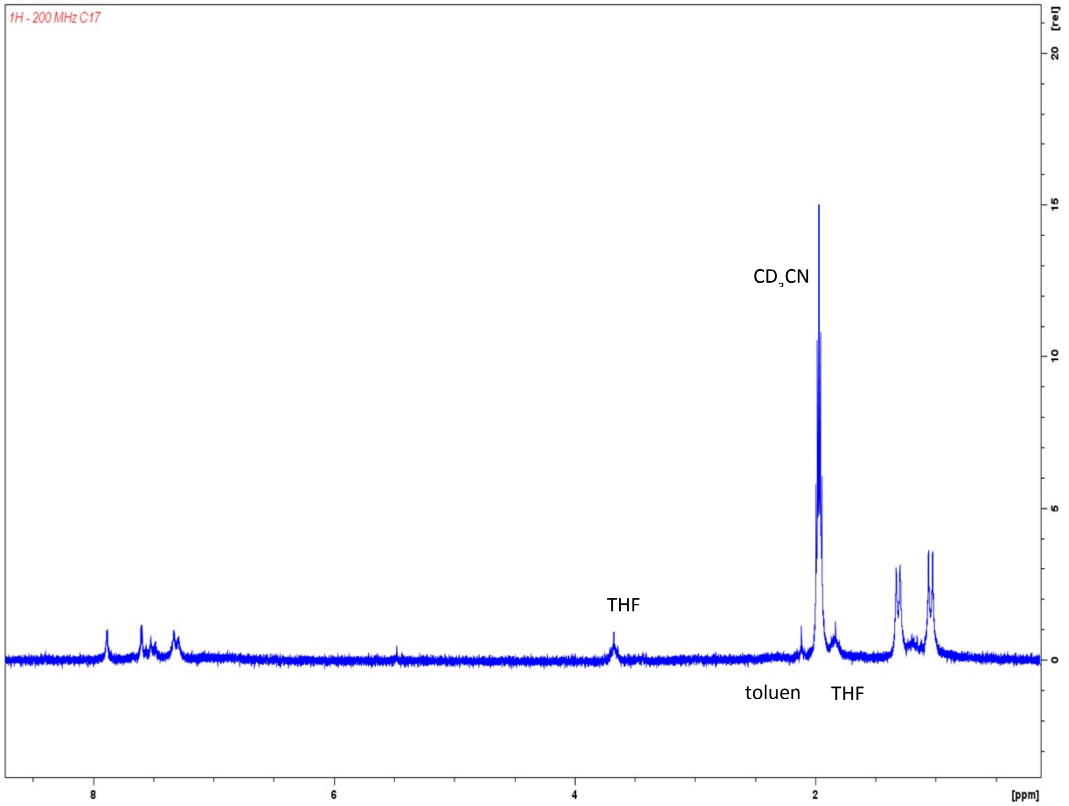

^1H NMR spectrum of **2**.

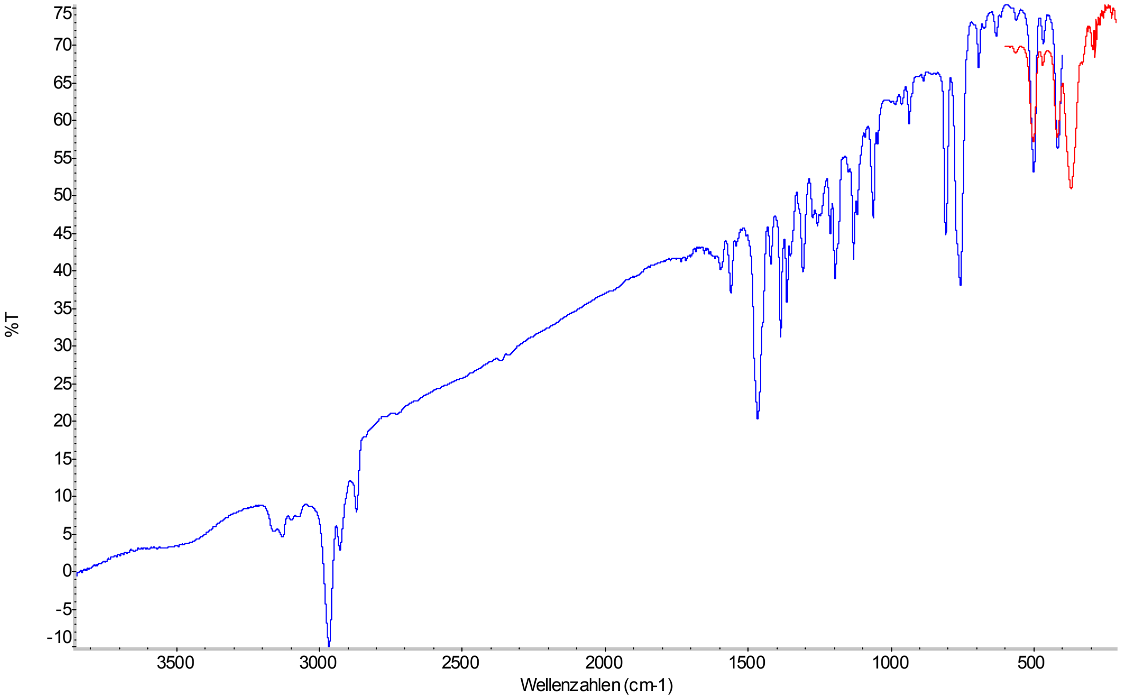

IR spectra (CsI) of **2**.

Synthesis and Characterisation of 3

To a solution of **1** (0.29 g, 0.40 mmol) in THF (20 mL) was added activated elemental selenium (Se_8, 0.063 g, 0.10 mmol) at -30 ^{o}C under stirring. The reaction mixture was allowed to warm to room temperature and stirred at that temperature for 2h. The volatiles were evaporated and the residue was washed with n-hexane (10 mL). After dried under vacuum compound **3** was obtained as a colorless powder with a yield of 0.31 g (0.35 mmol, 88%). M. p. 200 ºC (decomp.); ^{1}H NMR (200.13 MHz, [D_3] CH_3CN, 25ºC): δ = 1.04 (*d*, ^{3}J(H,H) = 7.0 Hz, 12 H; -CH(CH_3)$_3$), 1.33 (*d*, ^{3}J(H,H) = 7.0 Hz, 12 H; -CH(CH_3)$_3$), 2.90 – 3.20 (*m,* 4H; -C*H*(CH_3)$_3$), 6.94 – 7.05 (*m,* 2 H; CH_2), 7.31 (*d*, ^{3}J(H,H) = 7.0 Hz, 4 H; *m-Ph*), 7.52 (*dd*, ^{3}J(H,H) = 7.7 Hz, 7.8 Hz, 2 H; *p-Ph*), 7.59 (*d*, ^{3}J(H,H) = 2.0 Hz, 2 H; =C*H* (imid)), 7.87 (*d*, ^{3}J(H,H) = 2.0 Hz, 2 H; =C*H* (imid)); HR ESI-MS: *m*/*z*: 703.08749 (calc. 703.08680 [M-$GaCl_3$+H]$^+$, depicted below, top: exp.; bottom: calc.). IR (CsI, cm^{-1}): 2966(vs), 2928(s), 2869(s), 1562(w), 1467(vs), 1420(w), 1386(m), 1364(m), 1307(m), 1258(w), 1211(w), 1196(m), 1130(m), 1062(m), 807(s), 756(vs), 690(w), 631(w), 467(w), 415(w), 346(vs) ($\upsilon_{(Ge-Se)}$), 295(m), 285(m); elemental analysis calcd. (%) for $C_{31}H_{40}N_4GeGaCl_3Se_2$·THF: C 44.37, H 5.11, N 5.91; found: C 44.19, H 5.10, N 5.78.

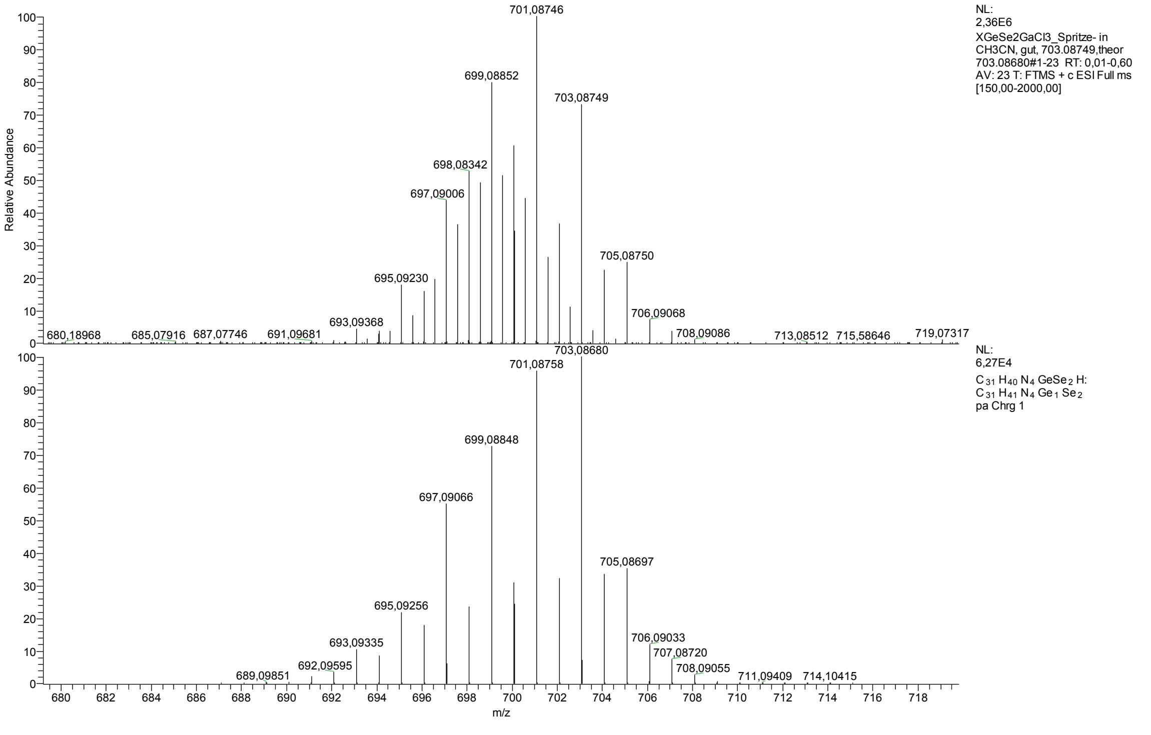

HR ESI-MS spectrum of **3**.

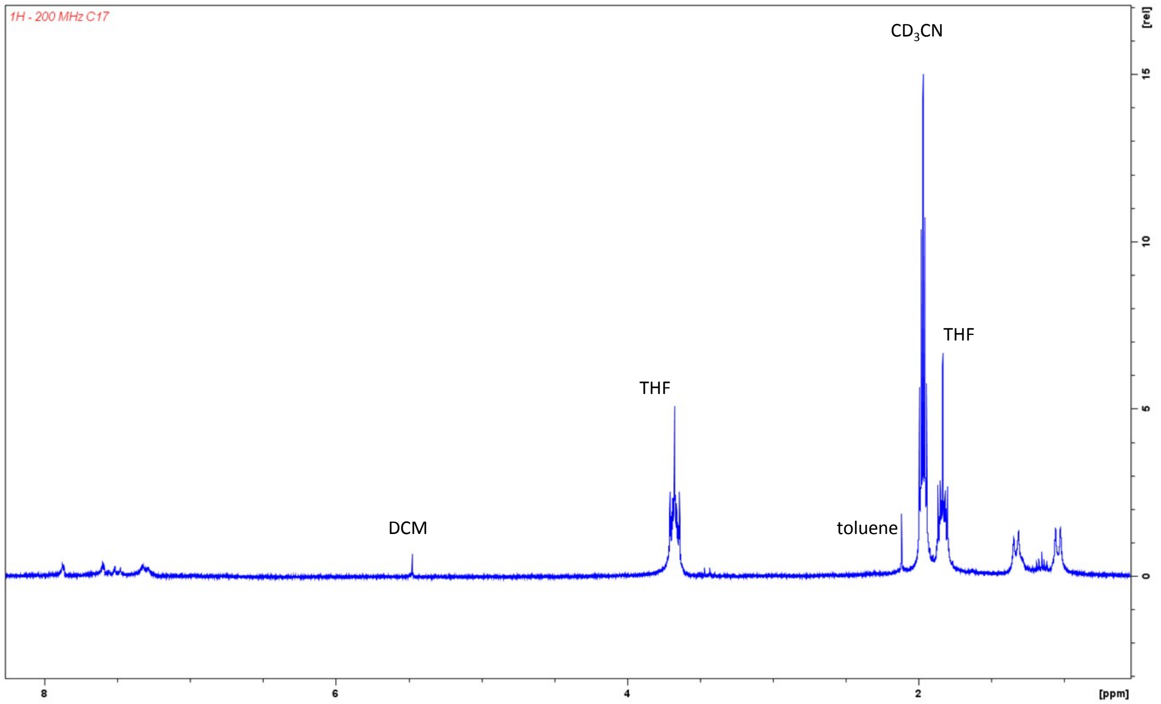

^{1}H NMR spectrum of **3**.

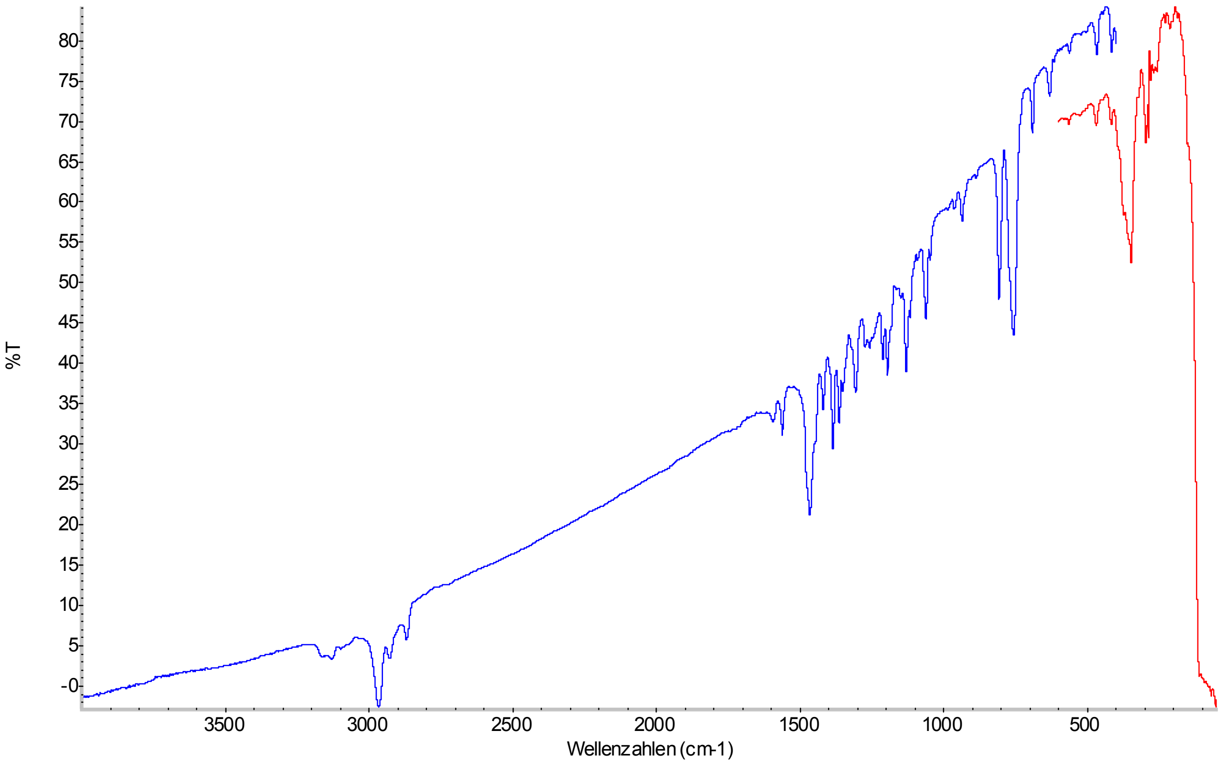

IR spectra (CsI) of **3**.

Synthesis and Characterisation of 4

At room temperature CH_3CN (50 mL) was added to a mixture of **1** (0.26 g, 0.36 mmol) and activated selenium Se_8 (0.029 g, 0.045 mmol) under stirring. After 3 days the selenium was completely consumed, and the solution was concentrated to 10 mL. At room temperature colorless crystals were formed slowly from the solution. The collected **4** amounts 0.18 g (0.23 mmol, 64%). M. p. 195 ºC (decomp.); ^1H NMR (200.13 MHz, $[D_3]$ CH_3CN, 25ºC): δ = 1.03 (*d*, ^3J(H,H) = 7.0 Hz, 6 H; -CH(CH_3)$_3$), 1.05 (*d*, ^3J(H,H) = 7.0 Hz, 6 H; -CH(CH_3)$_3$), 1.16 (*d*, ^3J(H,H) = 7.0 Hz, 6 H; -CH(CH_3)$_3$), 1.32 (*d*, ^3J(H,H) = 7.0 Hz, 6 H; -CH(CH_3)$_3$), 2.29 (sept., ^3J(H,H) = 7.0 Hz, 2 H; -C*H*(CH_3)$_3$), 3.07 (sept., ^3J(H,H) = 7.0 Hz, 2 H; -C*H*(CH_3)$_3$), 6.56, 7.02 (AB-spin system, ^2J = 13.4 Hz; 2 H; CH_2), 7.30 – 7.40 (*m*, 4 H; *Ph*), 7.50 – 7.58 (*m*, 2 H; *Ph*), 7.51 (*d*, ^3J(H,H) = 2.0 Hz, 2 H; =C*H* (imid)), 7.77 (*d*, ^3J(H,H) = 2.0 Hz, 2 H; =C*H* (imid)); $^{13}C\{^1H\}$ NMR (100.61 MHz, $[D_3]$ CH_3CN, 25 ºC): δ = 22.61, 22.88, 24.91, 25.46 (CH(*C*H_3)$_3$), 27.99, 28.59 (*C*H(CH_3)$_3$), 63.57 (*C*H_2), 123.21 (=*C*H(imid)), 124.23 (*m-Ph*), 124.86 (*m-Ph*), 126.24 (=*C*H(imid)), 131.47 (*p-Ph*), 132.60 (*i-Ph*), 145.71 (*o-Ph*), 147.43 (*o-Ph*), 167.00 ppm (carbene-*C*); HR ESI-MS: *m*/*z* = 577.21454 (calc. 577.21478 $[M\text{-}SeGaCl_3\text{+}Cl]^+$, depicted below, top: exp.; bottom: calc.). IR (CsI, cm^{-1}): 3130(w), 2969(vs), 2870(w), 1467(vs), 1410(s), 1386(m), 1365(m), 1347(w), 1296(s), 1243(vs), 1211(w), 1193(m), 1121(s), 1062(s), 984(s), 960(w), 938(w), 806(s), 770(s), 758(vs), 690(m), 628(w), 465(w), 417(w), 347(vs) ($\upsilon_{(Ge\text{-}Se)}$), 285(m); elemental analysis calcd. (%) for $C_{31}H_{40}N_4GeSeGaCl_3\cdot CH_3CN$: C 47.33, H 5.18, N 8.36; found: C 47.24, H 5.03, N 8.23.

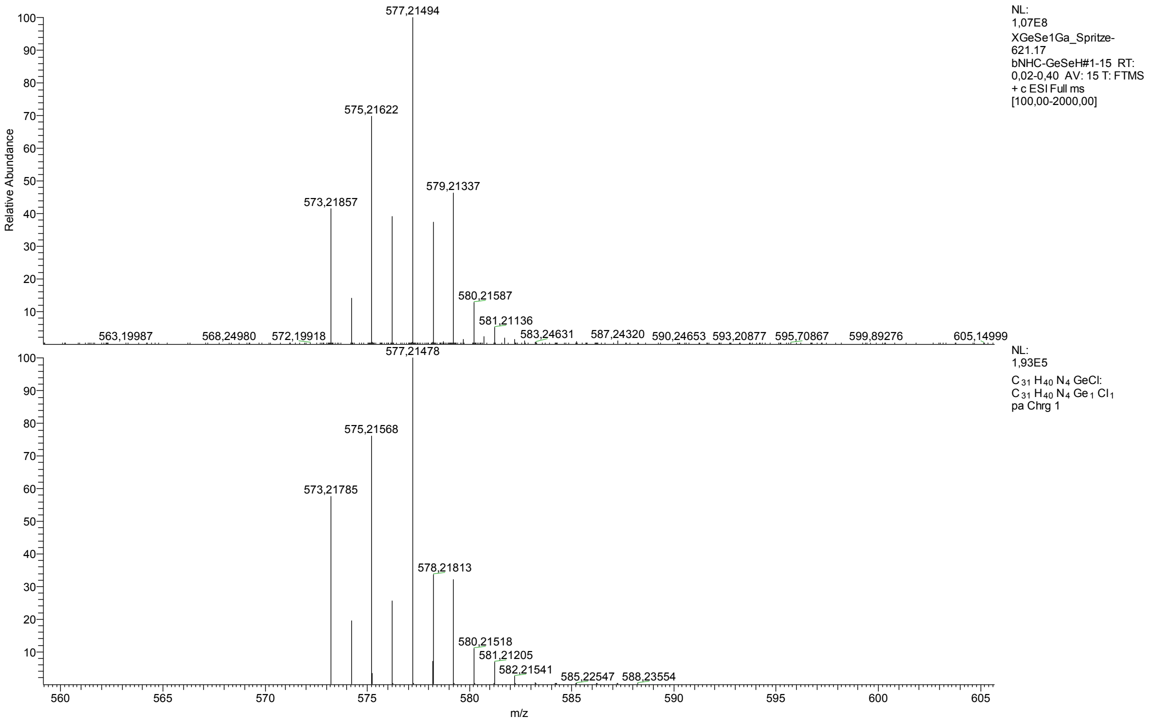

HR ESI-MS spectrum of **4**.

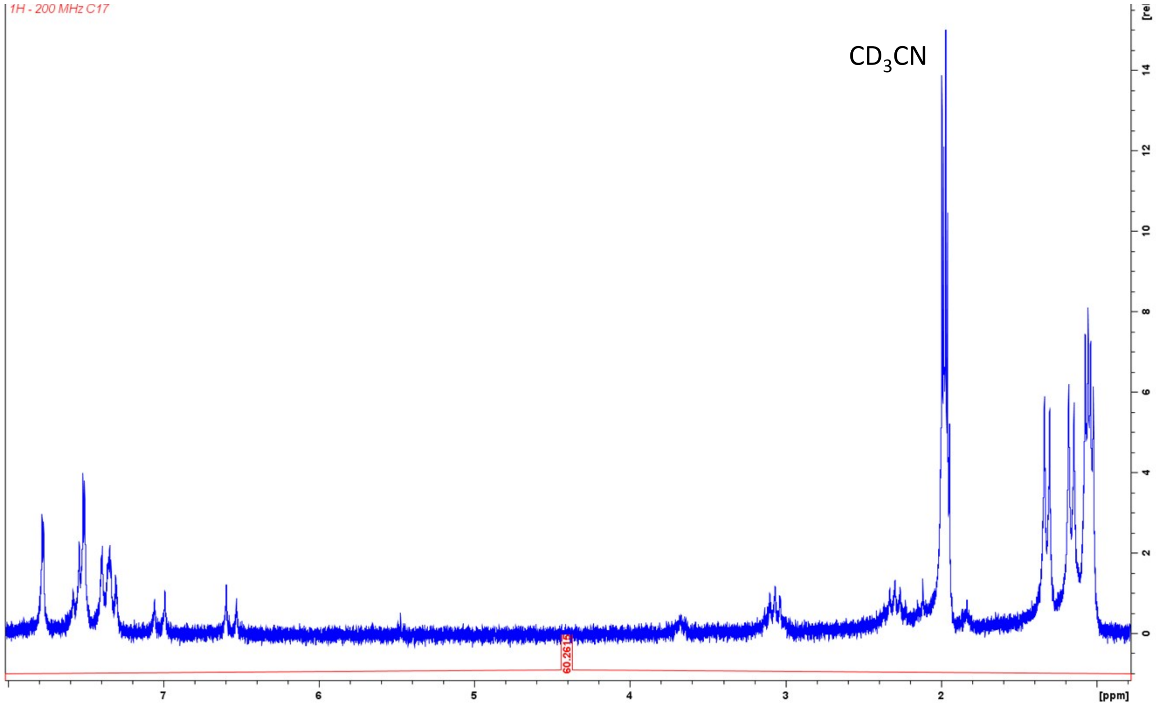

^{1}H NMR spectrum of **4**.

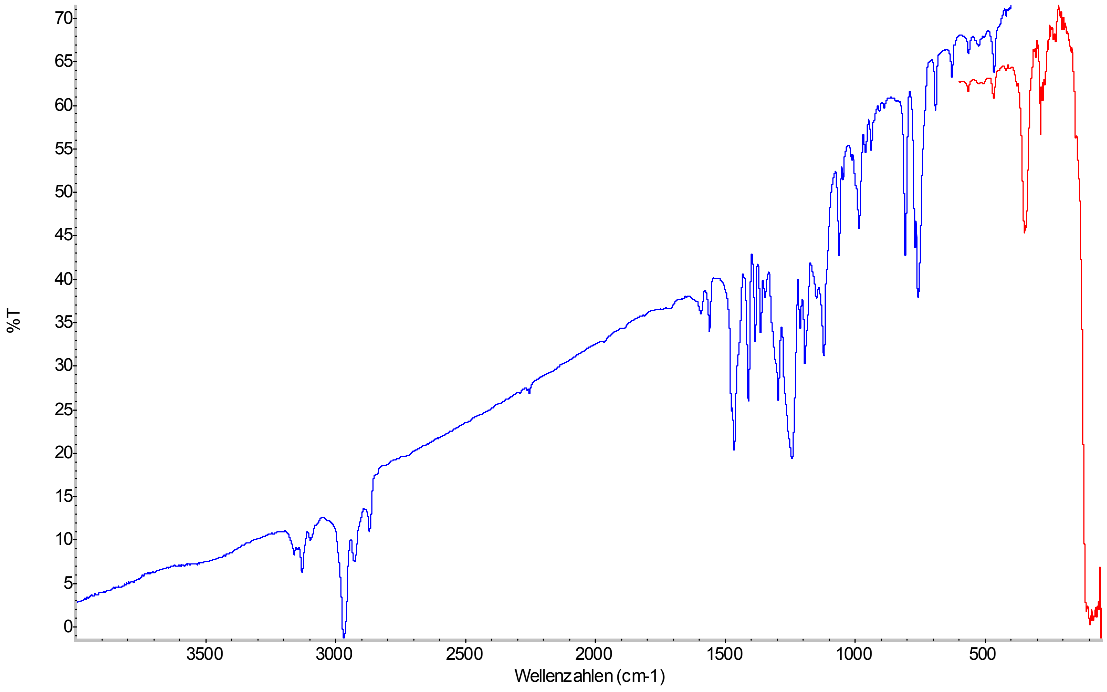

IR spectra (CsI) of **4**.

Synthesis and Characterisation of 5

At room temperature THF (25 mL) was added to a mixture of **1** (0.25 g, 0.35 mmol) and tellurium Te (0.044 g, 0.35 mmol) under stirring. After 3 days the tellurium was completely consumed, and the solution was concentrated to 10 mL. At room temperature pale yellow crystals were formed slowly from the solution. The collected **5** amounts to 0.21 g (0.25 mmol, 71%). M. p. 180 ºC (decomp.); ^{1}H NMR (200.13 MHz, [D$_3$] CH$_3$CN, 25ºC): δ = 1.04 (*d*, ^{3}J(H,H) = 7.0 Hz, 6 H; -CH(C*H*$_3$)$_3$), 1.06 (*d*, ^{3}J(H,H) = 7.0 Hz, 6 H; -CH(C*H*$_3$)$_3$), 1.19 (*d*, ^{3}J(H,H) = 7.0 Hz, 6 H; -CH(C*H*$_3$)$_3$), 1.36 (*d*, ^{3}J(H,H) = 7.0 Hz, 6 H; -CH(C*H*$_3$)$_3$), 2.37 (sept., ^{3}J(H,H) = 7.0 Hz, 2 H; -C*H*(CH$_3$)$_3$), 2.97 (sept., ^{3}J(H,H) = 7.0 Hz, 2 H; -C*H*(CH$_3$)$_3$), 6.56, 6.90 (AB-spin system, ^{2}J = 13.1 Hz; 2 H; C*H*$_2$), 7.32 – 7.38 (*m*, 4 H; *Ph*), 7.50 – 7.56 (*m*, 2 H; *Ph*), 7.53 (*d*, ^{3}J(H,H) = 2.0 Hz, 2 H; =C*H* (imid)), 7.79 (*d*, ^{3}J(H,H) = 2.0 Hz, 2 H; =C*H* (imid)); ^{13}C{^{1}H} NMR (100.61 MHz, [D$_3$] CH$_3$CN, 25 ºC): δ = 22.51, 22.92, 24.66, 25.29 (CH(*C*H$_3$)$_3$), 27.68, 28.25 (*C*H(CH$_3$)$_3$), 63.00 (*C*H$_2$), 123.19 (=*C*H(imid)), 124.02 (*m-Ph*), 124.71(*m-Ph*), 126.13 (=*C*H(imid)), 131.19 (*p-Ph*), 132.23(*i-Ph*), 145.31(*o-Ph*), 146.86 (*o-Ph*), 165.54 ppm (carbene-*C*); HR ESI-MS: *m*/*z*: = 577.21399 (calc. 577.21478 [M-Te-GaCl$_3$+Cl]$^{+}$, depicted below, top: exp.; bottom: calc.). IR (CsI, cm^{-1}): 3130(w), 2966(vs), 2867(m), 1563(w), 1466(vs), 1410(s), 1386(m), 1364(m), 1294(w), 1257(w), 1210(w), 1194(m), 1120(s), 1060(s), 806(s), 758(s), 744(s), 687(m), 625(w), 466(w), 361(vs), 336(s), 286(s), 278(m), 268(w), 254(w), 247(w), 226(s) ($\upsilon_{(Ge-Te)}$), 202(m); elemental analysis calc. (%) for $C_{31}H_{40}N_4GeTeGaCl_3\cdot 2THF$: C 47.36, H 5.71, N 5.66; found: C 47.15, H 5.55, N 5.53.

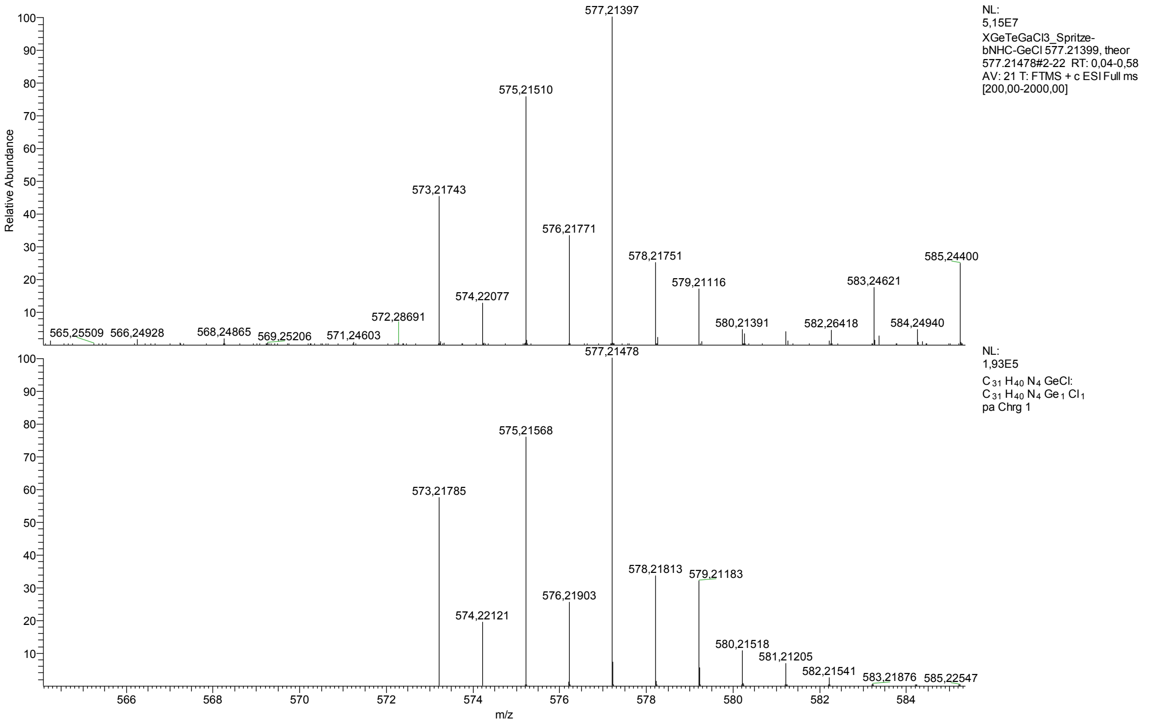

HR ESI-MS spectrum of **5**.

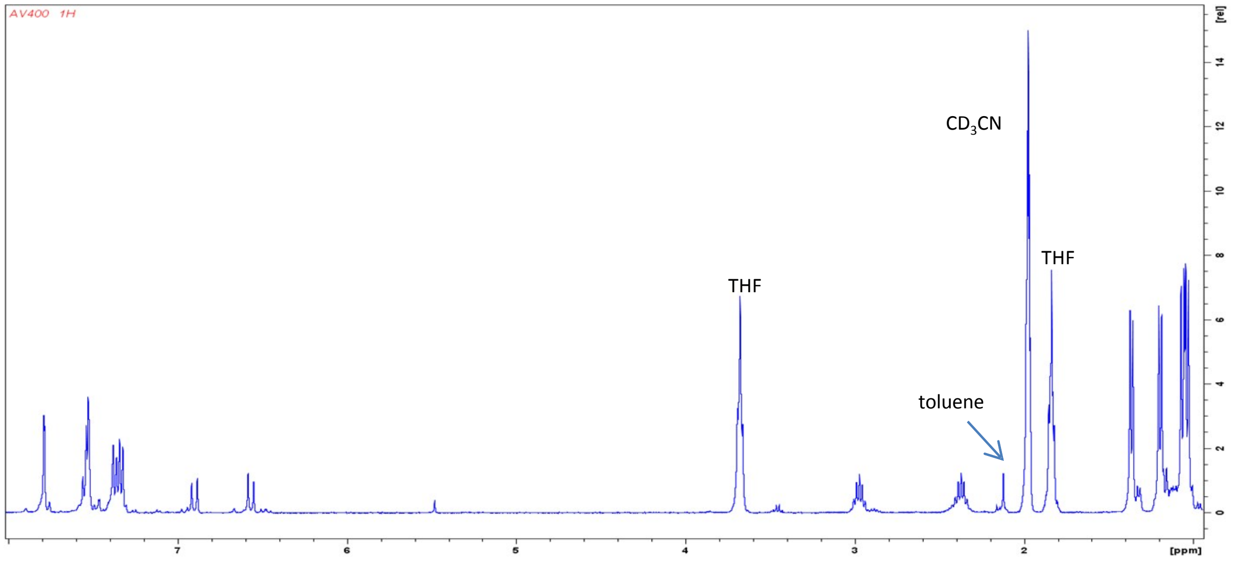

^{1}H NMR spectrum of **5**.

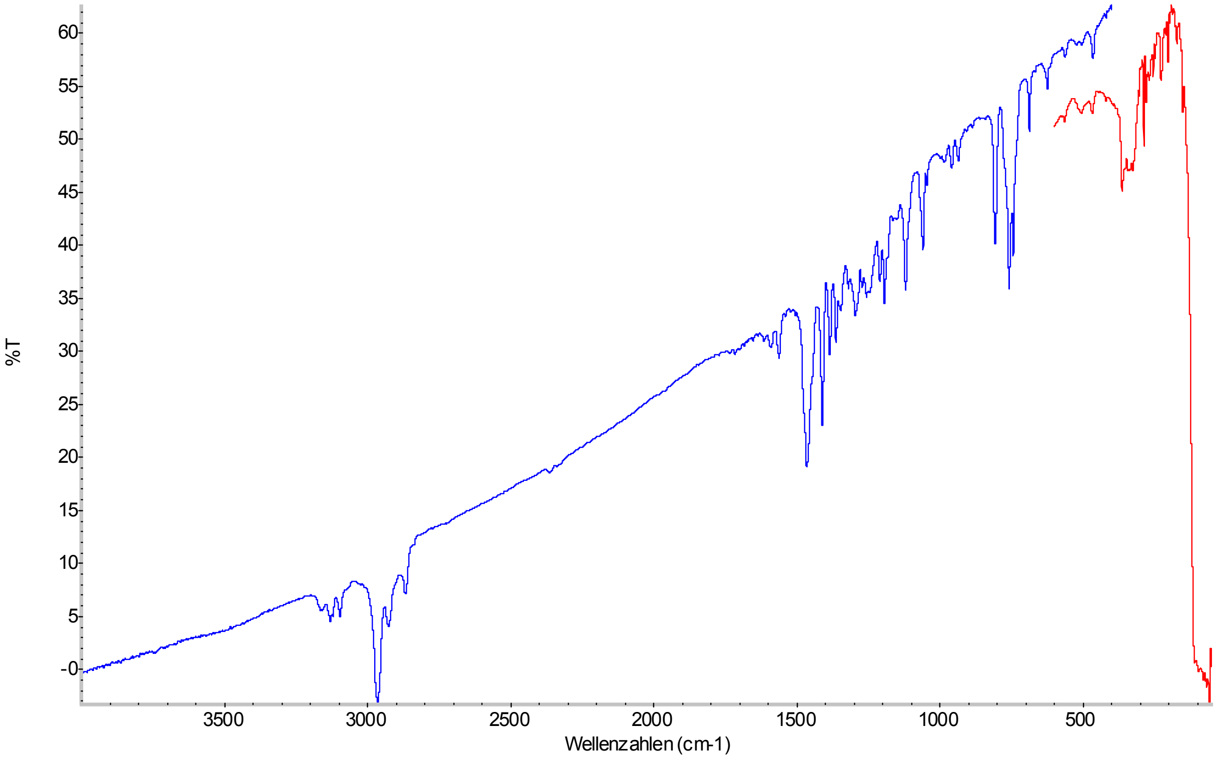

IR spectra (CsI) of **5**.

B. Crystallographic data

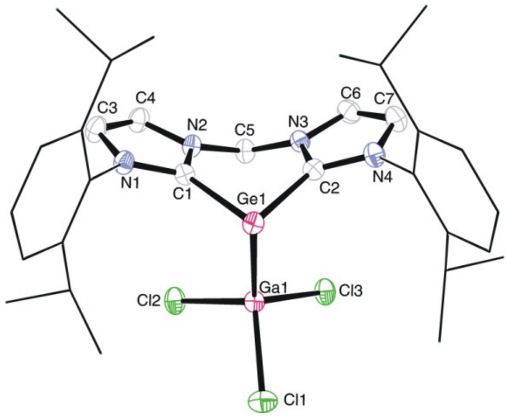

Figure S 1. Molecular structure of compound **1**. Thermal ellipsoids are drawn at 50% probability level. H atoms and four lattice solvent CH_3CN molecules are omitted for clarity.

Table S1. Crystal data and structure refinement for **1**.

Empirical formula	C39 H52 Cl3 Ga Ge N8	
Formula weight	881.55	
Temperature	150(2) K	
Wavelength	1.5418 Å	
Crystal system	Triclinic	
Space group	P-1	
Unit cell dimensions	a = 12.8149(4) Å	α= 109.690(3)°.
	b = 13.6703(4) Å	β= 90.728(2)°.
	c = 14.6341(4) Å	γ = 112.290(3)°.
Volume	2204.15(11) $Å^3$	
Z	2	
Density (calculated)	1.328 Mg/m^3	
Absorption coefficient	3.541 mm^{-1}	
F(000)	912	
Crystal size	0.23 x 0.18 x 0.10 mm^3	
Theta range for data collection	3.25 to 67.49°.	

Index ranges	-11<=h<=15, -16<=k<=13, -16<=l<=17
Reflections collected	14861
Independent reflections	7940 [R(int) = 0.0292]
Completeness to theta = 67.49°	99.5 %
Absorption correction	Semi-empirical from equivalents
Max. and min. transmission	1.00000 and 0.15591
Refinement method	Full-matrix least-squares on F^2
Data / restraints / parameters	7940 / 0 / 481
Goodness-of-fit on F^2	1.092
Final R indices [I>2sigma(I)]	R1 = 0.0458, wR2 = 0.1244
R indices (all data)	R1 = 0.0493, wR2 = 0.1268
Largest diff. peak and hole	1.685 and -0.514 e.Å^{-3}

Table S2. Selected interatomic distances [Å] and angles [°] for compound **1**.

Ge(1)-C(2)	2.033(3)
Ge(1)-C(1)	2.043(3)
Ge(1)-Ga(1)	2.5202(5)
Ga(1)-Cl(1)	2.2464(8)
Ga(1)-Cl(3)	2.2529(8)
Ga(1)-Cl(2)	2.2576(8)
N(1)-C(1)	1.349(4)
N(1)-C(3)	1.382(4)
C(1)-N(2)	1.354(4)
N(2)-C(4)	1.386(4)
N(2)-C(5)	1.461(4)
C(2)-N(4)	1.349(4)
C(2)-N(3)	1.360(4)
N(3)-C(6)	1.385(4)
N(3)-C(5)	1.454(4)
C(3)-C(4)	1.346(5)
N(4)-C(7)	1.389(4)
C(6)-C(7)	1.348(5)

C(2)-Ge(1)-C(1)	85.67(12)
C(2)-Ge(1)-Ga(1)	89.78(8)
C(1)-Ge(1)-Ga(1)	90.83(8)
Cl(1)-Ga(1)-Cl(3)	104.36(3)
Cl(1)-Ga(1)-Cl(2)	104.79(3)
Cl(3)-Ga(1)-Cl(2)	98.87(3)
Cl(1)-Ga(1)-Ge(1)	112.27(3)
Cl(3)-Ga(1)-Ge(1)	118.14(3)
Cl(2)-Ga(1)-Ge(1)	116.58(2)
C(1)-N(1)-C(3)	110.7(2)
N(1)-C(1)-N(2)	104.6(2)
N(1)-C(1)-Ge(1)	128.8(2)
N(2)-C(1)-Ge(1)	126.6(2)
C(1)-N(2)-C(4)	111.4(2)
C(1)-N(2)-C(5)	123.8(2)
C(4)-N(2)-C(5)	124.3(2)
N(4)-C(2)-N(3)	105.2(3)
N(4)-C(2)-Ge(1)	128.3(2)
N(3)-C(2)-Ge(1)	126.4(2)
C(2)-N(3)-C(6)	110.7(3)
C(2)-N(3)-C(5)	124.0(2)
C(6)-N(3)-C(5)	124.5(2)
C(4)-C(3)-N(1)	107.5(3)
C(2)-N(4)-C(7)	110.4(3)
C(3)-C(4)-N(2)	105.8(3)
N(3)-C(5)-N(2)	110.4(2)
C(7)-C(6)-N(3)	106.5(3)
C(6)-C(7)-N(4)	107.2(3)

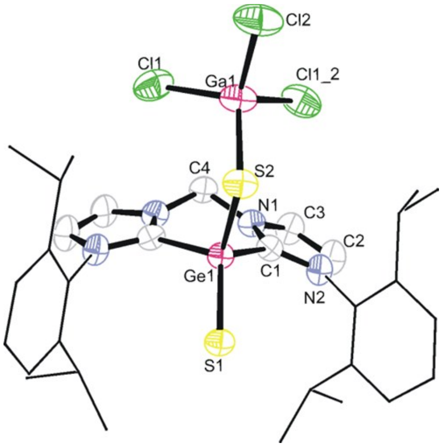

Figure S2. Molecular structure of compound **2**. Thermal ellipsoids are drawn at 50% probability level. H atoms and two lattice solvent THF molecules are omitted for clarity.

Table S3. Crystal data and structure refinement for **2**.

Empirical formula	C39 H56 Cl3 Ga Ge N4 O2 S2	
Formula weight	925.66	
Temperature	150(2) K	
Wavelength	1.54184 Å	
Crystal system	Monoclinic	
Space group	Cm	
Unit cell dimensions	a = 16.3230(2) Å	α= 90°.
	b = 21.5944(3) Å	β= 109.7720(10)°.
	c = 7.62520(10) Å	γ = 90°.
Volume	2529.32(6) $Å^3$	
Z	2	
Density (calculated)	1.215 Mg/m^3	
Absorption coefficient	3.865 mm^{-1}	
F(000)	960	
Crystal size	0.16 x 0.12 x 0.07 mm^3	
Theta range for data collection	3.53 to 67.50°.	
Index ranges	-19<=h<=17, -24<=k<=25, -8<=l<=9	
Reflections collected	8297	
Independent reflections	3656 [R(int) = 0.0215]	
Completeness to theta = 67.50°	99.4 %	

Absorption correction	Semi-empirical from equivalents
Max. and min. transmission	1.00000 and 0.61062
Refinement method	Full-matrix least-squares on F^2
Data / restraints / parameters	3656 / 2 / 248
Goodness-of-fit on F^2	1.123
Final R indices [I>2sigma(I)]	R1 = 0.0423, wR2 = 0.1193
R indices (all data)	R1 = 0.0435, wR2 = 0.1207
Absolute structure parameter	0.03(2)
Largest diff. peak and hole	1.188 and -0.431 e.$Å^{-3}$

Table S4. Selected interatomic distances [Å] and angles [°] for compound **2**.

Ge(1)-C(1)	1.998(3)
Ge(1)-S(1)	2.0873(12)
Ge(1)-S(2)	2.1981(13)
Ga(1)-Cl(2)	2.1810(18)
Ga(1)-Cl(1)	2.2027(14)
Ga(1)-S(2)	2.2493(15)
N(1)-C(1)	1.358(4)
N(1)-C(3)	1.370(5)
N(1)-C(4)	1.449(4)
C(1)-N(2)	1.329(5)
N(2)-C(2)	1.388(5)
N(2)-C(5)	1.457(5)
C(2)-C(3)	1.353(6)

C(1)-Ge(1)-S(1)	112.99(11)
C(1)-Ge(1)-S(2)	110.94(11)
S(1)-Ge(1)-S(2)	115.26(5)
Cl(2)-Ga(1)-Cl(1)	108.95(5)
Cl(2)-Ga(1)-S(2)	108.53(8)
Cl(1)-Ga(1)-S(2)	112.01(4)
C(1)-N(1)-C(3)	110.2(3)
C(1)-N(1)-C(4)	126.1(3)

C(3)-N(1)-C(4)	123.1(3)
N(2)-C(1)-N(1)	105.8(3)
N(2)-C(1)-Ge(1)	130.6(2)
N(1)-C(1)-Ge(1)	123.2(2)
Ge(1)-S(2)-Ga(1)	107.91(6)
C(1)-N(2)-C(2)	110.7(3)
C(1)-N(2)-C(5)	126.5(3)
C(2)-N(2)-C(5)	122.7(3)
C(3)-C(2)-N(2)	106.3(3)
C(2)-C(3)-N(1)	107.0(3)

Symmetry transformations used to generate equivalent atoms:

#1 x,-y+1,z

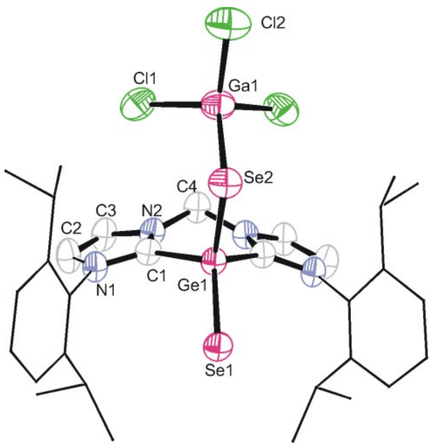

Figure S 3. Molecular structure of compound **3**. Thermal ellipsoids are drawn at 50% probability level. H atoms and two lattice solvent THF molecules are omitted for clarity.

Table S5. Crystal data and structure refinement for **3**.

Empirical formula	C39 H56 Cl3 Ga Ge N4 O2 Se2
Formula weight	1019.46
Temperature	150(2) K
Wavelength	1.54184 Å

Crystal system	Monoclinic	
Space group	Cm	
Unit cell dimensions	a = 16.2434(7) Å	α= 90°.
	b = 21.8215(9) Å	β= 110.197(4)°.
	c = 7.6828(3) Å	γ = 90°.
Volume	2555.76(18) Å^3	
Z	2	
Density (calculated)	1.325 Mg/m^3	
Absorption coefficient	4.691 mm^{-1}	
F(000)	1032	
Crystal size	0.18 x 0.13 x 0.05 mm^3	
Theta range for data collection	3.54 to 67.46°.	
Index ranges	-19<=h<=18, -25<=k<=26, -9<=l<=8	
Reflections collected	7876	
Independent reflections	3421 [R(int) = 0.0326]	
Completeness to theta = 67.46°	99.6 %	
Absorption correction	Semi-empirical from equivalents	
Max. and min. transmission	1.00000 and 0.58147	
Refinement method	Full-matrix least-squares on F^2	
Data / restraints / parameters	3421 / 2 / 248	
Goodness-of-fit on F^2	1.086	
Final R indices [I>2sigma(I)]	R1 = 0.0443, wR2 = 0.1221	
R indices (all data)	R1 = 0.0456, wR2 = 0.1245	
Absolute structure parameter	0.03(3)	
Largest diff. peak and hole	1.265 and -0.701 e.Å^{-3}	

Table S6. Crystal data and structure refinement for **3**.

Se(1)-Ge(1)	2.2144(9)
Ge(1)-C(1)	1.987(5)
Ge(1)-Se(2)	2.3263(10)
Ga(1)-Cl(2)	2.179(3)
Ga(1)-Cl(1)	2.2086(18)

Ga(1)-Se(2)	2.3738(12)
C(1)-N(1)	1.352(6)
C(1)-N(2)	1.366(6)
C(2)-C(3)	1.361(8)
C(2)-N(1)	1.362(7)
C(3)-N(2)	1.383(7)
C(4)-N(2)	1.442(6)
C(1)-Ge(1)-Se(1)	112.96(14)
C(1)-Ge(1)-Se(2)	110.84(15)
Se(1)-Ge(1)-Se(2)	115.19(4)
Cl(2)-Ga(1)-Cl(1)	108.74(7)
Cl(2)-Ga(1)-Se(2)	108.41(10)
Cl(1)-Ga(1)-Se(2)	111.94(5)
N(1)-C(1)-N(2)	105.0(4)
N(1)-C(1)-Ge(1)	131.6(3)
N(2)-C(1)-Ge(1)	123.0(3)
C(3)-C(2)-N(1)	107.7(5)
C(2)-C(3)-N(2)	106.0(4)
C(1)-N(2)-C(3)	110.4(4)
C(1)-N(2)-C(4)	126.5(4)
C(3)-N(2)-C(4)	122.6(4)
Ge(1)-Se(2)-Ga(1)	104.88(4)

Symmetry transformations used to generate equivalent atoms:

#1 x,-y,z

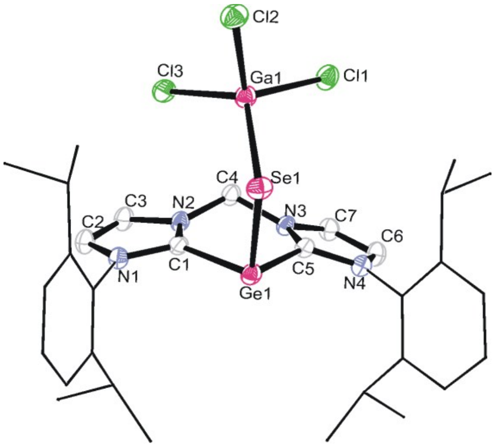

Figure S 4. Molecular structure of compound **4**. Thermal ellipsoids are drawn at 50% probability level. H atoms and one lattice solvent CH_3CN molecule are omitted for clarity.

Table S7. Crystal data and structure refinement for **4**.

Empirical formula	C33 H43 Cl3 Ga Ge N5 Se	
Formula weight	837.34	
Temperature	150(2) K	
Wavelength	1.54184 Å	
Crystal system	Monoclinic	
Space group	P21/c	
Unit cell dimensions	a = 12.53200(10) Å	α= 90°.
	b = 18.88550(10) Å	β= 90.0910(10)°.
	c = 16.07690(10) Å	γ = 90°.
Volume	3804.96(4) Å^3	
Z	4	
Density (calculated)	1.462 Mg/m^3	
Absorption coefficient	5.094 mm^{-1}	
F(000)	1696	
Crystal size	0.27 x 0.19 x 0.14 mm^3	
Theta range for data collection	3.53 to 67.50°.	
Index ranges	-12<=h<=15, -22<=k<=21, -17<=l<=19	
Reflections collected	24155	

Independent reflections	6855 [R(int) = 0.0353]
Completeness to theta = 67.50°	99.9 %
Absorption correction	Semi-empirical from equivalents
Max. and min. transmission	1.00000 and 0.44228
Refinement method	Full-matrix least-squares on F^2
Data / restraints / parameters	6855 / 0 / 406
Goodness-of-fit on F^2	1.070
Final R indices [I>2sigma(I)]	R1 = 0.0373, wR2 = 0.0995
R indices (all data)	R1 = 0.0381, wR2 = 0.1002
Largest diff. peak and hole	1.436 and -0.819 e.Å^{-3}

Table S8. Crystal data and structure refinement for **4**.

Se(1)-Ga(1)	2.3370(4)
Se(1)-Ge(1)	2.4385(4)
Ge(1)-C(1)	2.047(2)
Ge(1)-C(5)	2.052(3)
Ga(1)-Cl(3)	2.2041(7)
Ga(1)-Cl(1)	2.2108(7)
Ga(1)-Cl(2)	2.2205(7)
N(1)-C(1)	1.344(3)
N(1)-C(2)	1.386(3)
C(1)-N(2)	1.351(3)
N(2)-C(3)	1.387(3)
N(2)-C(4)	1.451(3)
C(2)-C(3)	1.340(4)
N(3)-C(5)	1.353(3)
N(3)-C(7)	1.381(3)
N(3)-C(4)	1.462(3)
N(4)-C(5)	1.346(3)
N(4)-C(6)	1.387(3)
C(6)-C(7)	1.342(4)

Ga(1)-Se(1)-Ge(1)	110.724(15)
C(1)-Ge(1)-C(5)	86.07(10)
C(1)-Ge(1)-Se(1)	99.62(7)
C(5)-Ge(1)-Se(1)	103.79(7)
Cl(3)-Ga(1)-Cl(1)	106.06(3)
Cl(3)-Ga(1)-Cl(2)	105.07(3)
Cl(1)-Ga(1)-Cl(2)	107.69(3)
Cl(3)-Ga(1)-Se(1)	117.99(2)
Cl(1)-Ga(1)-Se(1)	112.20(2)
Cl(2)-Ga(1)-Se(1)	107.22(2)
C(1)-N(1)-C(2)	110.4(2)
N(1)-C(1)-N(2)	105.3(2)
N(1)-C(1)-Ge(1)	124.23(18)
N(2)-C(1)-Ge(1)	129.37(18)
C(1)-N(2)-C(3)	110.6(2)
C(1)-N(2)-C(4)	125.4(2)
C(3)-N(2)-C(4)	123.3(2)
C(3)-C(2)-N(1)	107.2(2)
C(5)-N(3)-C(7)	110.9(2)
C(5)-N(3)-C(4)	125.5(2)
C(7)-N(3)-C(4)	123.1(2)
C(2)-C(3)-N(2)	106.4(2)
C(5)-N(4)-C(6)	110.9(2)
N(2)-C(4)-N(3)	111.7(2)
N(4)-C(5)-N(3)	104.8(2)
N(4)-C(5)-Ge(1)	124.62(18)
N(3)-C(5)-Ge(1)	128.10(18)
C(7)-C(6)-N(4)	106.7(2)
C(6)-C(7)-N(3)	106.7(2)

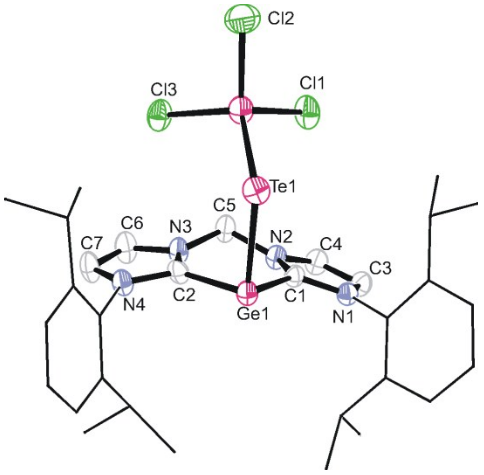

Figure S 5. Molecular structure of compound **5**. Thermal ellipsoids are drawn at 50% probability level. H atoms and three lattice solvent THF molecules are omitted for clarity.

Table S9. Crystal data and structure refinement for **5**.

Empirical formula	C43 H64 Cl3 Ga Ge N4 O3 Te	
Formula weight	1061.24	
Temperature	150(2) K	
Wavelength	1.54184 Å	
Crystal system	Triclinic	
Space group	p-1	
Unit cell dimensions	a = 11.3788(3) Å	α= 69.253(2)°.
	b = 13.0772(3) Å	β= 83.744(2)°.
	c = 17.5125(4) Å	γ = 84.543(2)°.
Volume	2417.94(10) $Å^3$	
Z	2	
Density (calculated)	1.458 Mg/m^3	
Absorption coefficient	7.950 mm^{-1}	
F(000)	1080	
Crystal size	0.17 x 0.11 x 0.05 mm^3	
Theta range for data collection	2.71 to 67.49°.	
Index ranges	-9<=h<=13, -15<=k<=15, -20<=l<=20	
Reflections collected	14971	

Independent reflections	8672 [R(int) = 0.0369]
Completeness to theta = 67.49°	99.4 %
Absorption correction	Semi-empirical from equivalents
Max. and min. transmission	1.00000 and 0.31666
Refinement method	Full-matrix least-squares on F^2
Data / restraints / parameters	8672 / 88 / 559
Goodness-of-fit on F^2	1.086
Final R indices [I>2sigma(I)]	R1 = 0.0560, wR2 = 0.1475
R indices (all data)	R1 = 0.0623, wR2 = 0.1531
Largest diff. peak and hole	2.197 and -1.761 e.Å^{-3}

Table S10. Crystal data and structure refinement for **5**.

Te(1)-Ga(1)	2.5262(7)
Te(1)-Ge(1)	2.6538(6)
Ge(1)-C(2)	2.034(5)
Ge(1)-C(1)	2.040(4)
Ga(1)-Cl(2)	2.2027(16)
Ga(1)-Cl(3)	2.2310(13)
Ga(1)-Cl(1)	2.2379(14)
N(1)-C(1)	1.350(6)
N(1)-C(3)	1.391(6)
C(1)-N(2)	1.357(6)
N(2)-C(4)	1.380(6)
N(2)-C(5)	1.453(6)
C(2)-N(4)	1.348(6)
C(2)-N(3)	1.353(6)
N(3)-C(6)	1.381(6)
N(3)-C(5)	1.459(6)
C(3)-C(4)	1.347(7)
N(4)-C(7)	1.380(6)
C(6)-C(7)	1.361(8)

Ga(1)-Te(1)-Ge(1)	108.95(2)
C(2)-Ge(1)-C(1)	86.40(18)
C(2)-Ge(1)-Te(1)	97.69(13)

C(1)-Ge(1)-Te(1)	101.76(12)
Cl(2)-Ga(1)-Cl(3)	105.05(6)
Cl(2)-Ga(1)-Cl(1)	106.40(7)
Cl(3)-Ga(1)-Cl(1)	104.24(5)
Cl(2)-Ga(1)-Te(1)	112.38(5)
Cl(3)-Ga(1)-Te(1)	115.41(4)
Cl(1)-Ga(1)-Te(1)	112.54(4)
C(1)-N(1)-C(3)	110.2(4)
N(1)-C(1)-N(2)	105.4(4)
N(1)-C(1)-Ge(1)	124.9(3)
N(2)-C(1)-Ge(1)	128.3(3)
C(1)-N(2)-C(4)	110.4(4)
C(1)-N(2)-C(5)	126.3(4)
C(4)-N(2)-C(5)	122.5(4)
N(4)-C(2)-N(3)	104.9(4)
N(4)-C(2)-Ge(1)	125.6(3)
N(3)-C(2)-Ge(1)	129.1(3)
C(2)-N(3)-C(6)	111.4(4)
C(2)-N(3)-C(5)	126.6(4)
C(6)-N(3)-C(5)	121.6(4)
C(4)-C(3)-N(1)	106.8(4)
C(2)-N(4)-C(7)	110.9(4)
C(3)-C(4)-N(2)	107.0(4)
N(2)-C(5)-N(3)	112.0(4)
C(7)-C(6)-N(3)	105.9(4)
C(6)-C(7)-N(4)	106.9(4)

Symmetry transformations used to generate equivalent atoms:

C. Computational Details

C.1. Computational methods

The calculations were performed using Gaussian 09 Rev. D.01[3] and ORCA version 3.0.2.[4] Compounds **1**-**5** were calculated at B3LYP[5]-D3(BJ)/def2-TZVPP[6] and at ZORA[7]-BP86[5a,8]-D3(BJ)/def2-TZVP(-f)[6], employing the DFT-D3 empirical dispersion corrections of Grimme (D3)[9] with Becke-Johnson (BJ)[10] damping. Model compounds, where the Dipp substituent on the nitrogen atoms were replaced by R=Ph (e.g., 1') as well and model compounds with R=Me (e.g., 1'') were optimized at B3LYP-D3(BJ)/def2-SV[6]. Solvent effects were calculated by optimizing the geometries in acetonitrile and THF using the Polarizable Continuum Model (PCM).[11] Harmonic frequencies were calculated to characterise the optimised compounds as minima or transition states. Free energies are calculated at 1 atm. and 298.15 K. The calculated optimised geometries of the experimental compounds and of the model compounds (with all three methods used) are in very good agreement with experiment. NBO 6.0[12] was used to calculate the Natural Population Analysis (NPA) charges[13] and Wiberg bond indices (WBI).[14] The resonance structures and their weights were calculated using Natural Resonance Theory (NRT).[15] These properties were calculated using the optimised geometries at B3LYP-D3(BJ)/def2-SV. Resonance structures were calculated for the model compounds with R=Me.

^{77}Se and ^{125}Te chemical shifts were calculated numerically for the experimental compounds **3**, **4** and **5** at ZORA-M06L/def2-TZVP(-f)//ZORA-PB86-D3(BJ)/def2-TVZP(-f) using the IGLO[16] method. The reference compounds are $SeMe_2$ (σ (^{77}Se) = 1726.4 ppm (calc.) (2069 ppm (exp.)[17a,b]) and $TeMe_2$ (σ(^{125}Te)=2829.0 ppm (calc.) 3388 ppm (exp.)[18]).

C.2. Optimized geometries

Table S11. Measured and calculated geometry parameters (bond lengths in Å, bond angles in degrees) for compounds **1**-**5** (R=Dipp[a]) and calculated values for model compounds **1'**-**5'** (R=Ph[b]).

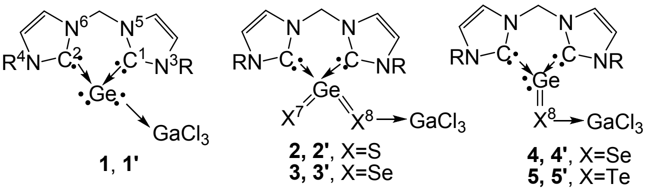

1, **1'**

2, 2', X=S
3, 3', X=Se

4, 4', X=Se
5, 5', X=Te

Parameter		**1**<br>R=Dipp,<br>**1‘**(R=Ph)	**2**<br>R=Dipp,<br>**2‘**(R=Ph)	**3**<br>R=Dipp,<br>**3‘**(R=Ph)	**4**<br>R=Dipp,<br>**4‘**(R=Ph)	**5**<br>R=Dipp,<br>**5‘**(R=Ph)
r(Ge-Gal)	Exp. R=Dipp	2.520				
	Calc. R=Dipp	2.588	-	-	-	-
	Calc. R=Ph	2.549				
r(Ge-C1)	Exp. R=Dipp	2.043	1.997	1.987	2.025	2.040
	Calc. R=Dipp[c]	2.040	2.089	2.029	2.067	2.058
				2.020	*2.065*	*2.053*
	Calc. R=Ph	2.048	2.022	2.020	2.072	2.058
r(Ge-C2)	Exp. R=Dipp	2.033	1.997	1.987	2.047	2.034
	Calc. R=Dipp[c]	2.056	2.089	2.029	2.065	2.059
				2.020	*2.061*	*2.052*
	Calc. R=Ph	2.045	2.020	2.020	2.071	2.058
r(C1-N3)	Exp. R=Dipp	1.349	1.329	1.352	1.346	1.350
	Calc. R=Dipp[c]	1.350	1.342	1.344	1.348	1.349
				1.353	*1.357*	*1.358*
	Calc. R=Ph	1.356	1.344	1.344	1.352	1.352
r(C1-N5)	Exp. R=Dipp	1.354	1.358	1.366	1.353	1.357
	Calc. R=Dipp[c]	1.355	1.349	1.350	1.352	1.352
				1.359	*1.361*	*1.362*
	Calc. R=Ph	1.360	1.354	1.354	1.358	1.359
r(C2-N4)	Exp. R=Dipp	1.348	1.329	1.352	1.344	1.348
	Calc. R=Dipp[c]	1.351	1.342	1.344	1.348	1.349
				1.353	*1.357*	*1.359*
	Calc. R=Ph	1.356	1.343	1.344	1.351	1.352
Exp. R=Dipp	Exp. R=Dipp	1.359	1.358	1.359	1.351	1.353
Calc. R=Dipp[c]	Calc. R=Dipp[c]	1.354	1.349	1.350	1.352	1.352
				1.359	*1.361*	*1.363*
Calc. R=Ph	Calc. R=Ph	1.360	1.354	1.354	1.358	1.359
r(Ge-X7)	Exp. R=Dipp		2.087	2.214		
	Calc. R=Dipp[c]	-	2.081	2.219	-	-
				2.224		
	Calc. R=Ph		2.091	2.231		
r(Ge-X8)	Exp. R=Dipp	-	2.198	2.326	2.438	2.654
	Calc. R=Dipp		2.208	2.347	2.427	2.634
				2.351	*2.438*	*2.644*
	Calc. R=Ph		2.213	2.347	2.435	2.654
r(X8-Ga)	Exp. R=Dipp	-	2.249	2.374	2.337	2.526
	Calc. R=Dipp		2.275	2.403	2.388	2.587
				2.397	*2.381*	*2.587*
	Calc. R=Ph		2.280	2.404	2.381	2.582
∠C1-Ge-C2)	Exp. R=Dipp	85.7	91.3	91.8	86.1	86.4
	Calc. R=Dipp	84.5	88.6	89.2	85.1	85.6
				89.3	84.4	*84.9*
	Calc. R=Ph	85.4	89.2	89.3	85.2	85.3
∠X7-Ge-X8)	Exp. R=Dipp	-	115.3	115.2		
	Calc. R=Dipp[c]		120.3	119.4	-	-
				119.4		
	Calc. R=Ph		125.6	126.2		
∠Ge-X8-Ga)	Exp. R=Dipp	-	107.9	104.8	110.7	109.0
	Calc. R=Dipp[c]		109.2	105.8	110.4	105.2
				104.0	*109.5*	*104.4*
	Calc. R=Ph		102.5	98.7	104.6	99.9

[a] Optimised at B3LYP-D3(BJ)/def2-TZVPP; [b] Optimised at B3LYP-D3(BJ)/def2-SV; [c] In italics, optimised geometries at ZORA-BP86-D3(BJ)/def2-TVZP(-f)

Table S12. Optimised[a,b] geometry parameters (bond lengths in Å, bond angles in degrees) of the stationary points on the $GaCl_3$ migration path from **C**→**D**[c]

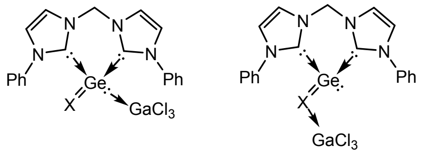

C, X = S, Se **D**, X = S

Parameter	**C(S)**	**C(Se)**	**D**(X=S)	**TS1** C(S)→INT(S)	**TS1** C(Se)→INT(Se)	**INT(S)**	**INT(Se)**	**TS2 (S)**	**TS2 (Se)**
r(Ge-GaI)	2.479	2.480	-	2.547	2.566	-	-	-	-
r(Ge-C1)	2.062	2.063	2.082	2.023	2.017	2.105	2.102	1.890	1.886
r(Ge-C2)	2.068	2.063	2.082	2.023	2.016	2.111	2.105	1.883	1.894
r(C1-N3)	1.344	1.346	1.352	1.352	1.353	1.353	1.354	1.371	1.375
r(C1-N5)	1.353	1.353	1.358	1.360	1.361	1.359	1.359	1.380	1.386
r(C2-N4)	1.346	1.346	1.352	1.356	1.356	1.356	1.357	1.376	1.371
r(C2-N6)	1.354	1.353	1.358	1.363	1.364	1.361	1.361	1.386	1.380
r(Ge-X7)	2.100	2.238	-	2.143	2.281	-	-	-	-
r(Ge-X8)	-	-	2.300	-	-	2.263	2.402	2.149	2.275
r(X8-Ga)	-	-	2.259	2.990	3.080	2.292	2.418	2.341	2.463
∠C1-Ge-C2)	85.9	86.3	85.0	86.7	87.3	81.9	82.6	98.2	97.8
∠Ge-X8-Ga)	-	-	108.1	-	-	95.4	92.5	100.2	96.5
∠X-Ge-Ga	143.0[d]	143.2 [d]	-	78.7 [d]	78.7 [d]	-	-	-	-

[a] Optimised at B3LYP-D3(BJ)/def2-SV; [b] For atom numbering see Table S11; [c] See Figure S7 for the reaction path and for a schematic presentation of the optimised structures.

C.3. Charge distribution

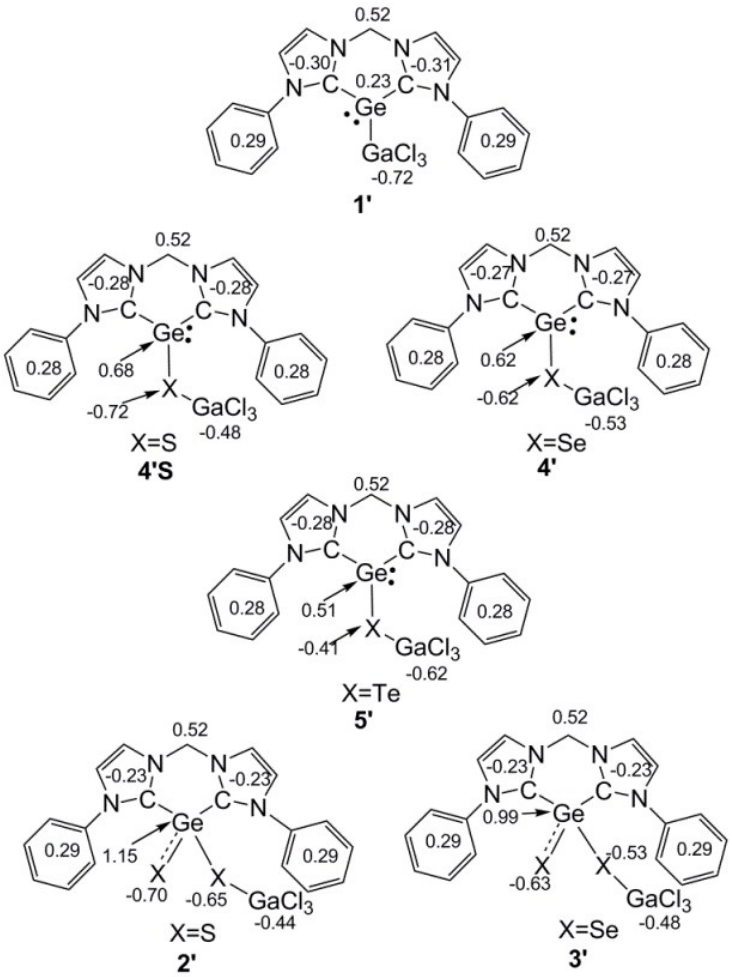

Figure S6. Calculated NPA charge distributions (at B3LYP-D3(BJ)/def2-SV) in compounds **1'**-**5'** and in the sulphur analog of **4'**(X=S).

C.4. Reaction free energies

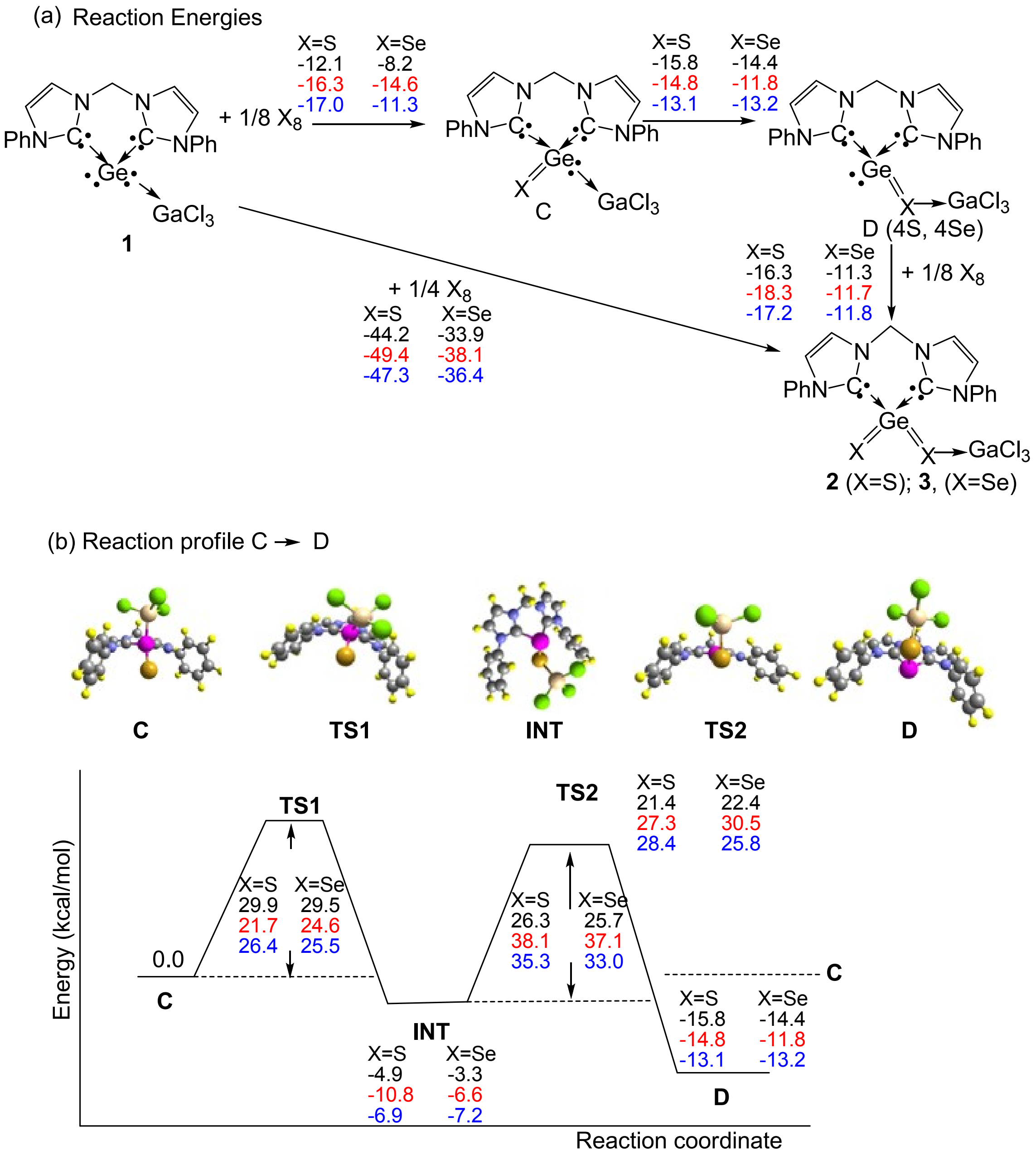

Figure S7. (a) Calculated free energies for the formation of **2**, **3** and **4** from **1**. (b) Reaction Profile for the migration of $GaCl_3$ from C to D (X=S and Se). Gas phase (black), acetonitrile (red), THF (blue).

C.5. Resonance structures: NRT results

2”

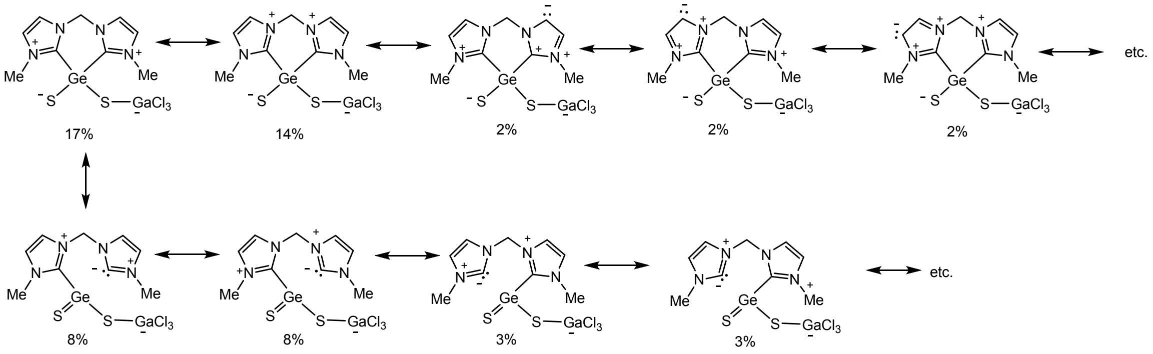

3”

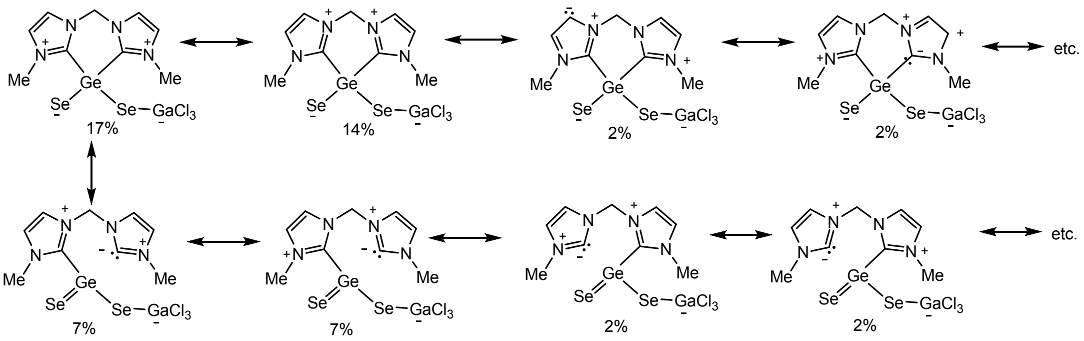

4”

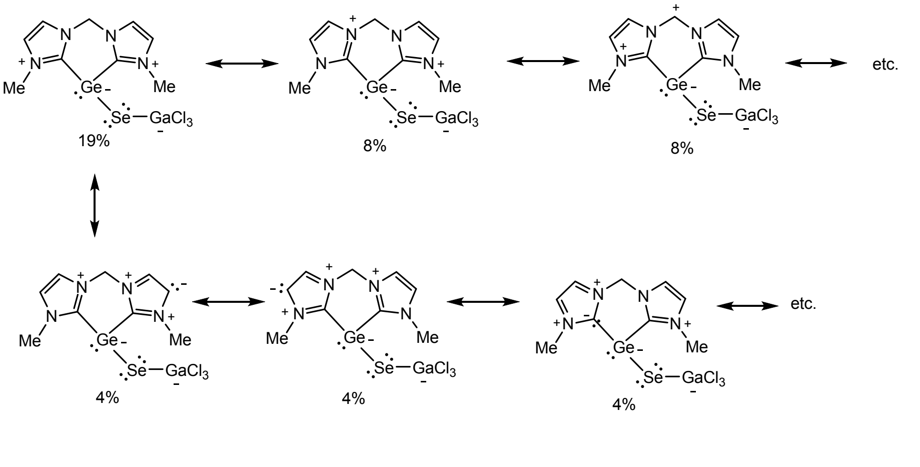

5“

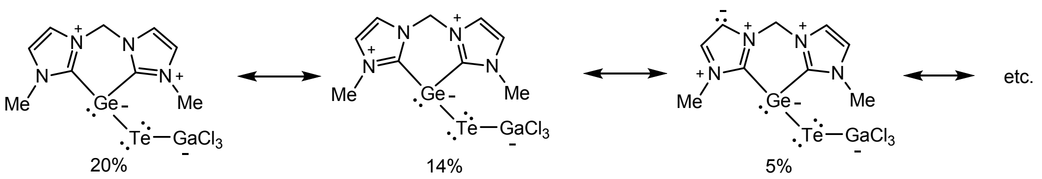

Scheme S1. Leading resonance structures (RS) for **2”, 3”** and **4”** calculated using the NRT method at B3LYP-D3(BJ)/def2-SV and of **5**” calculated at B3LYP-D3(BJ)/def2-SV (SDD for Te) . The percentage represents their weight. Smaller weight permutations exist.

C.6. NMR calculations of ^{77}Se chemical shifts in compounds 3 and 4 and ^{125}Te chemical shift in compound 5.

Table S13. Calculated[a] ^{77}Se chemical shifts in **3** (R=Dipp), **4** (R=Dipp) and ^{125}Te chemical shift in **5** (R=Dipp).

	**3**	**4**	**5**
$\delta(^{77}$Se7)	-400.8[a]; -308.4[b]	-	-
$\delta(^{77}$Se8)	-308.4[a]; -223.5[b]	-515.0[a]; -515.0[b]	-
$\delta(^{125}$Te)	-	-	-1055.5[a] -1042.9[b]

[a] Chemical shifts in ppm, calculated at ZORA-MO6L/def2-TVZP(-f)//ZORA-BP86-D3(BJ)/def2-TZVP(-f). $SeMe_2$ and $TeMe_2$ are used as reference compounds. $\sigma(^{77}\mathbf{Se}Me_2)$=1726.4 ppm (exp. 2069 ppm ref.17 a,b), $\sigma(^{125}\mathbf{Te}Me_2)$=2829.0 (exp. 3388 ppm ref.18); Atom numbering according to the numbering in Table S11; [b] Geometry optimized at B3LYP-D3(BJ)/def2-TVZPP.

C.6.1. Choosing the computational method for the NMR calculations

^{77}Se NMR

The ^{77}Se NMR chemical shifts in a variety of organic compounds containing Se (**6-19**) covering a broad range of chemical shifts was calculated using 4 different density functionals i.e. M06, B3LYP, PBE0 and B97 using ZORA with the def2-TZVP(-f) basis set. Their geometry was optimized at ZORA-BP86-D3(BJ)/def2-TZVP(-f). $SeMe_2$ was used as the reference compound. The experimental[17] and calculated NMR chemical shifts are given in the Table below along with linear correlation charts. All functionals give good correlations between experiment and theory; however, M06L yields the best result in terms of the slope→1. We therefore, choose M06L as the method for calculating ^{77}Se NMR isotropic shifts.

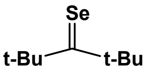
6

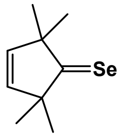
7

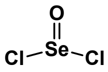
8

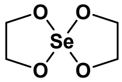
9

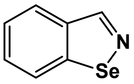
10

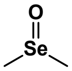
11

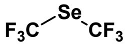
12

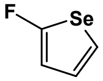
13

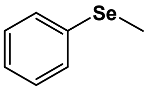
14

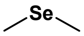
15

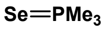
16

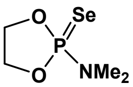
17

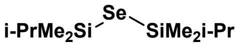
18

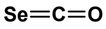
19

Compound	δ(^{77}Se) exp.	δ(^{77}Se) calc. M06L	δ(^{77}Se) calc. B3LYP	δ(^{77}Se) calc. PBE0	δ(^{77}Se) calc. B97
**6**	2131	1886.2	2501.1	2465.5	2442.1
**7**	1803	1526.7	2034.1	2030.6	1996.5
**8**	1483	1379.1	1624.2	1617.2	1604.1
**9**	1261	1211.5	1370.4	1371.6	1366.9
**10**	1013	887.9	1085.1	1100.9	1080.7
**11**	812	719	792.1	803.8	796.4
**12**	717	588.5	792	761.3	761
**13**	513	389.8	491.6	519.4	496.9
**14**	202	185.6	239.7	238	238.5
**15**	0	0	0	0	0
**16**	-235	-483.6	-399.6	-377.5	-395.6
**17**	-317	-437.8	-368.4	-337.3	-359.1
**18**	-419	-688.4	-675.6	-634	-669.1
**19**	-447	-616.5	-616.5	-547.9	-585.3

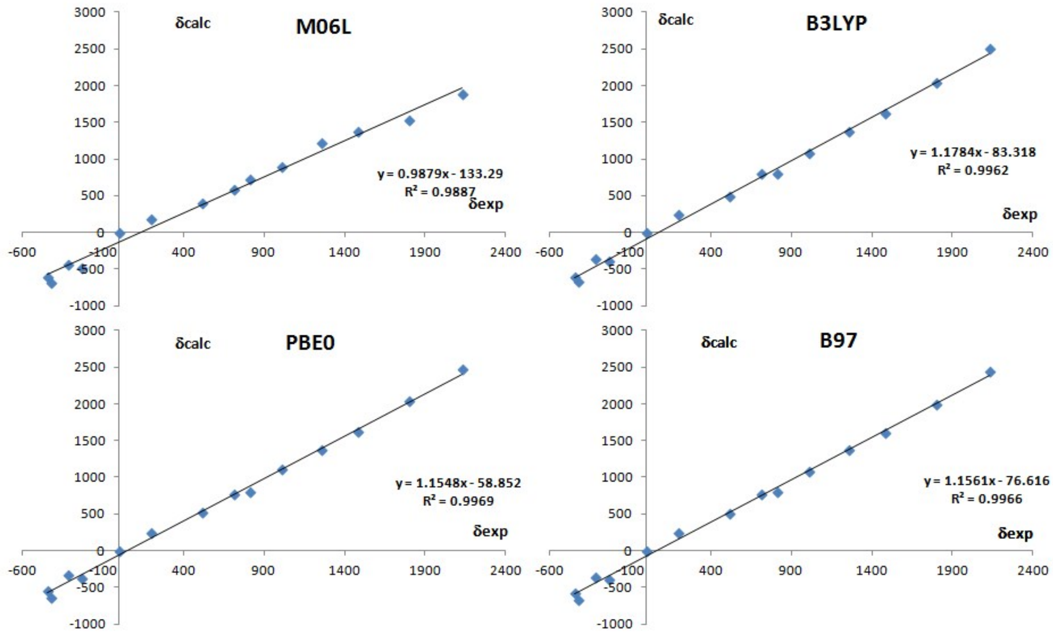

^{125}Te NMR

Similarly we calculated the ^{125}Te chemical shifts for the fourteen compounds (**20**-**33**) given below. The reference compound is $TeMe_2$. The experimental[18] and calculated NMR chemical shift are given in the table below along with linear correlation charts. The best performing functional in terms of the correlation coefficient R^2 and slope→1 is M06L.

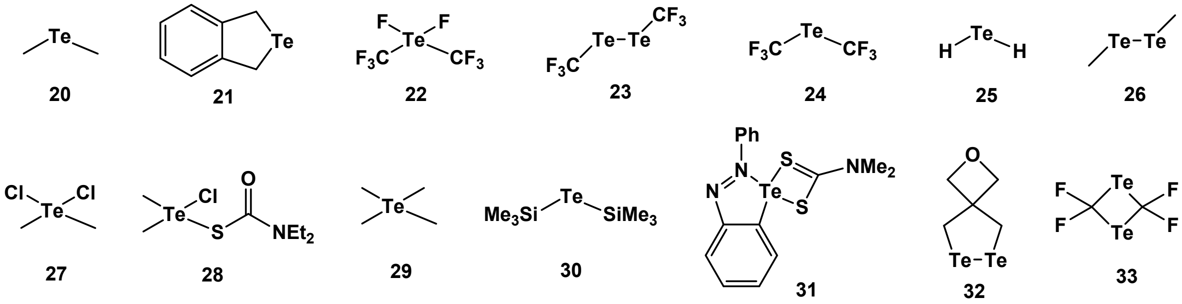

Compound	δ(^{125}Te) exp	δ(^{125}Te) calc M06L	δ(^{125}Te) calc B3LYP	δ(^{125}Te) calc PBE0	δ(^{125}Te) calc B97
**20**	0	0	0	0	0
**21**	268	124.9	187.7	206.5	195.1
**22**	1187	790.9	895.2	943	927.9

**23**	686	624.1	1003.6	915.6	940.7
**24**	1368	1170.3	1523	1452.4	1459.7
**25**	-621	-721.2	-657	-603.9	-627.1
**26**	63	59.3	272.4	225.2	243.3
**27**	733.8	414.5	361.1	403	387.6
**28**	554.1	477.5	436.5	467.2	463.4
**29**	-67	-260.3	-382.9	-327.2	-346.3
**30**	-842	-1292.3	-1291.9	-1220.3	-1279.8
**31**	1228.6	924.7	1201.8	1259.7	1213
**32**	57.1	46.9	271.3	239.7	248.4
**33**	2321.7	2374.7	2828.5	2734.4	2762.2

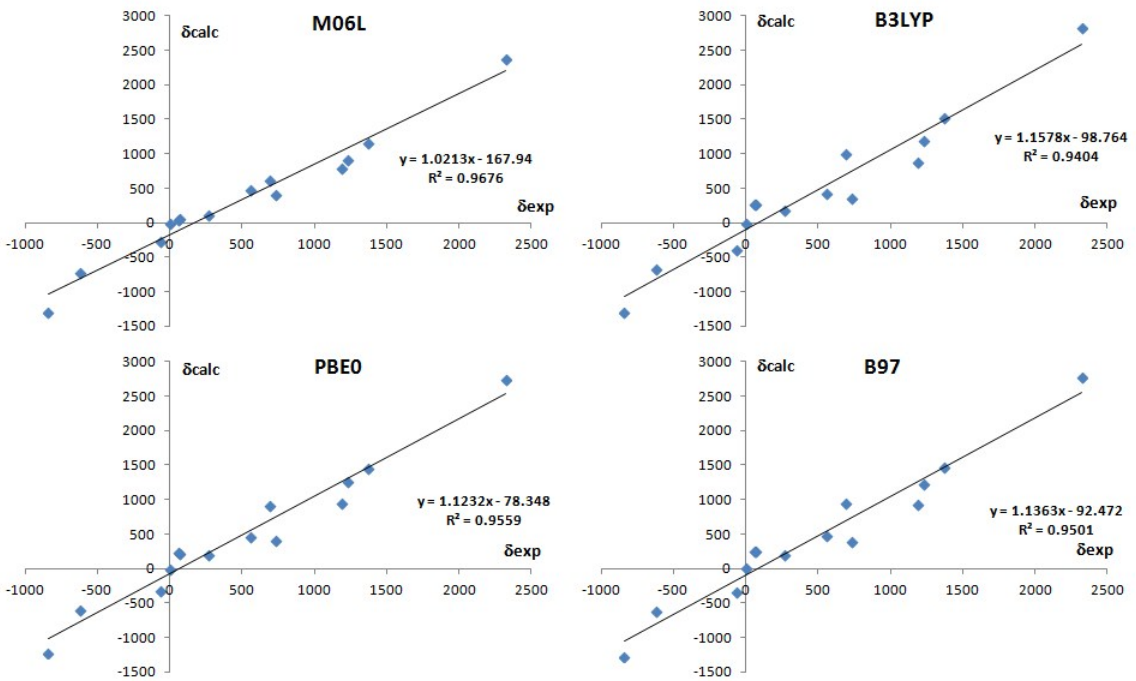

C.6. Calculated molecular xyz coordinates; energies in Hartrees, free energies at 298.15K

a) 1-5 (R=Dipp). B3LYP-D3(BJ)/def2-TZVPP

1

E= -6807.9874529
Free energy = -6807.407136

32	-0.017403000	0.103375000	-0.482055000
31	-0.222527000	-2.448101000	-0.079156000
17	-0.325208000	-3.624959000	-1.955230000
7	2.713002000	0.416650000	0.895899000
6	1.387267000	0.165222000	1.018232000
17	1.453598000	-3.302390000	1.215768000
7	1.203292000	-0.094354000	2.334661000
6	-1.383470000	0.306712000	1.037362000
17	-2.041632000	-2.955219000	1.200159000
7	-1.192010000	0.063034000	2.355822000
6	3.352979000	0.258222000	2.116476000
1	4.411892000	0.392011000	2.220306000
7	-2.664204000	0.734817000	0.946302000
6	2.404768000	-0.058943000	3.022027000
1	2.476711000	-0.280904000	4.070211000
6	-0.030473000	-0.621946000	2.883177000
1	-0.099483000	-1.683558000	2.640521000
1	-0.013619000	-0.491997000	3.961483000
6	-2.347063000	0.304794000	3.081100000
1	-2.408937000	0.136068000	4.139780000
6	-3.269962000	0.732242000	2.195259000
1	-4.290130000	1.037969000	2.326172000
6	3.383809000	0.813812000	-0.321352000
6	3.913642000	-0.193210000	-1.131714000
6	4.613615000	0.199445000	-2.272145000
1	5.034543000	-0.558196000	-2.917164000
6	4.761480000	1.535925000	-2.591851000
1	5.293349000	1.821627000	-3.489495000
6	4.224026000	2.515941000	-1.769808000
1	4.346922000	3.553788000	-2.039928000
6	3.533400000	2.183517000	-0.608118000
6	3.033955000	3.273127000	0.324809000
1	2.128013000	2.907441000	0.810798000
6	2.676237000	4.567053000	-0.408697000
1	2.194961000	5.260767000	0.280132000
1	1.998800000	4.384639000	-1.239734000
1	3.565458000	5.065483000	-0.796020000
6	4.074610000	3.584254000	1.413473000
1	4.275897000	2.726647000	2.049604000
1	3.724459000	4.397318000	2.051598000
1	5.015402000	3.894240000	0.956190000
6	3.735700000	-1.660682000	-0.814063000
1	3.001927000	-1.758462000	-0.020285000
6	5.042825000	-2.269962000	-0.291507000
1	5.815886000	-2.263137000	-1.062243000
1	4.872921000	-3.302134000	0.014879000
1	5.424186000	-1.719964000	0.570416000
6	3.199022000	-2.450030000	-2.011921000
1	2.320484000	-1.972387000	-2.443376000
1	2.902073000	-3.447201000	-1.691918000
1	3.949416000	-2.552065000	-2.797889000
6	-3.290328000	1.280819000	-0.234495000
6	-4.104500000	0.447021000	-1.002933000
6	-4.682029000	0.994880000	-2.148957000
1	-5.318870000	0.376128000	-2.764731000
6	-4.435297000	2.303956000	-2.517492000
1	-4.882600000	2.705441000	-3.416958000
6	-3.606580000	3.103979000	-1.743722000
1	-3.405896000	4.117041000	-2.059418000
6	-3.021504000	2.615999000	-0.580252000
6	-2.124922000	3.502466000	0.262654000
1	-1.416910000	2.858087000	0.780913000
6	-1.292809000	4.472992000	-0.571790000
1	-0.722898000	3.935835000	-1.329057000
1	-0.589995000	4.999792000	0.070570000
1	-1.907745000	5.218937000	-1.075785000
6	-2.946313000	4.253609000	1.322220000
1	-3.760625000	4.808477000	0.853919000
1	-2.318582000	4.960666000	1.866458000
1	-3.385907000	3.567663000	2.045701000
6	-4.349139000	-1.005710000	-0.656767000
1	-3.873100000	-1.222360000	0.296603000
6	-5.841945000	-1.315437000	-0.502329000
1	-6.304419000	-0.684155000	0.258365000
1	-5.973330000	-2.356636000	-0.207063000
1	-6.385279000	-1.163378000	-1.435996000
6	-3.699836000	-1.917581000	-1.705127000
1	-4.217258000	-1.841335000	-2.663458000
1	-3.729998000	-2.954385000	-1.375428000
1	-2.654922000	-1.662978000	-1.870209000

2

E= -7604.5278382

Free energy = -7603.947719

32	-0.000070000	0.410736000	-0.409448000	6	4.965351000	-1.772346000	-0.164987000
31	0.000500000	-3.214619000	0.060277000	1	4.845416000	-2.835074000	0.044458000
17	-1.766614000	-3.107862000	1.443126000	1	5.079462000	-1.257519000	0.789398000
16	-0.000364000	2.192517000	-1.485340000	1	5.885499000	-1.631043000	-0.734962000
7	1.207792000	0.032127000	2.334050000	17	1.767525000	-3.107328000	1.443196000
6	1.421420000	0.333891000	1.036236000	7	-1.207848000	0.031794000	2.334038000
17	0.000782000	-5.096849000	-1.043836000	6	-1.421555000	0.333480000	1.036217000
16	0.000305000	-1.527543000	-1.466862000	7	-2.719131000	0.665444000	0.945048000
7	2.718907000	0.666196000	0.945072000	6	-3.333694000	0.534094000	2.175549000
6	3.333514000	0.534957000	2.175563000	1	-4.377123000	0.743591000	2.307692000
1	4.376899000	0.744675000	2.307701000	6	-2.386126000	0.131323000	3.052034000
6	2.386040000	0.131974000	3.052052000	1	-2.444250000	-0.105453000	4.097659000
1	2.444227000	-0.104808000	4.097672000	6	-3.377174000	1.164063000	-0.245013000
6	0.000051000	-0.564244000	2.876055000	6	-3.864002000	0.239744000	-1.175102000
1	0.000197000	-1.632365000	2.663013000	6	-4.633106000	2.126730000	-2.478636000
1	0.000024000	-0.405010000	3.950329000	1	-5.122142000	2.507092000	-3.365403000
6	3.376821000	1.164938000	-0.245008000	6	-4.496755000	0.759206000	-2.302248000
6	3.863987000	0.240677000	-1.174971000	1	-4.879023000	0.083093000	-3.051946000
6	4.632505000	2.127761000	-2.478702000	6	-4.136717000	3.011786000	-1.534775000
1	5.121443000	2.508181000	-3.365499000	1	-4.235551000	4.074837000	-1.696985000
6	4.496621000	0.760213000	-2.302150000	6	-3.488633000	2.550610000	-0.393389000
1	4.879148000	0.084136000	-3.051749000	6	-2.959281000	3.525288000	0.642367000
6	4.135767000	3.012764000	-1.534973000	1	-2.213682000	3.004775000	1.243683000
1	4.234237000	4.075828000	-1.697317000	6	-4.090137000	3.984510000	1.575355000
6	3.487815000	2.551509000	-0.393545000	1	-4.579817000	3.144397000	2.067894000
6	2.958105000	3.526139000	0.642076000	1	-3.702879000	4.651547000	2.347202000
1	2.212658000	3.005456000	1.243433000	1	-4.852022000	4.525596000	1.012376000
6	4.088782000	3.985868000	1.575033000	6	-2.253336000	4.731984000	0.018604000
1	4.578749000	3.145991000	2.067685000	1	-2.951442000	5.374045000	-0.519729000
1	3.701274000	4.652862000	2.346792000	1	-1.795731000	5.336138000	0.803384000
1	4.850487000	4.527156000	1.012003000	1	-1.473446000	4.403831000	-0.664846000
6	2.251777000	4.732522000	0.018147000	6	-3.753057000	-1.254595000	-0.952817000
1	2.949680000	5.374750000	-0.520249000	1	-2.872253000	-1.443100000	-0.345878000
1	1.793955000	5.336620000	0.802843000	6	-3.568990000	-2.050184000	-2.245102000
1	1.472009000	4.404026000	-0.665279000	1	-4.474895000	-2.057117000	-2.853609000
6	3.753522000	-1.253666000	-0.952508000	1	-2.749055000	-1.651191000	-2.838821000
1	2.872687000	-1.442394000	-0.345682000	1	-3.329262000	-3.084584000	-2.000072000
6	3.569941000	-2.049485000	-2.244724000	6	-4.964833000	-1.773809000	-0.165564000
1	4.475947000	-2.056186000	-2.853083000	1	-4.844571000	-2.836528000	0.043732000
1	2.749967000	-1.650848000	-2.838627000	1	-5.079259000	-1.259178000	0.788888000
1	3.330526000	-3.083937000	-1.999601000	1	-5.884943000	-1.632723000	-0.73565500

3

E = -11611.2798373
Free energy = -11610.699268

34	-2.370135000	-1.391234000	0.000000000	6	-0.436760000	2.293841000	3.341068000
32	-0.442571000	-0.290351000	0.000000000	1	-0.621390000	2.432207000	4.388025000
31	3.320589000	0.154262000	0.000000000	6	-0.019861000	3.155762000	2.387538000
17	3.179048000	1.530794000	1.771686000	1	0.256500000	4.191786000	2.441608000
6	-0.309566000	1.148171000	1.425060000	6	0.641447000	2.962271000	0.000000000
17	5.233173000	-0.901959000	0.000000000	1	0.504976000	4.039798000	0.000000000

1	1.705251000	2.728496000	0.000000000
6	-1.140158000	-0.101539000	3.408959000
6	-0.229843000	-1.043928000	3.899425000
6	-2.135133000	-2.297137000	4.706465000
1	-2.528499000	-3.170293000	5.209476000
7	0.035131000	2.435259000	1.208238000
6	-3.005501000	-1.337623000	4.214331000
1	-4.069956000	-1.473964000	4.332050000
6	-2.528375000	-0.213665000	3.547903000
6	-3.484762000	0.848230000	3.035965000
1	-2.983166000	1.390301000	2.233524000
6	-3.828446000	1.846148000	4.153234000
1	-4.350869000	1.339510000	4.966095000
1	-4.479358000	2.635113000	3.773369000
1	-2.940581000	2.315240000	4.575412000
7	-0.622082000	1.068515000	2.729918000
6	-4.768294000	0.266126000	2.439544000
1	-4.534773000	-0.468692000	1.672905000
1	-5.356051000	1.064864000	1.985047000
1	-5.391841000	-0.200359000	3.203120000
6	1.268579000	-0.851951000	3.777767000
1	1.466629000	-0.286073000	2.871430000
6	1.805500000	-0.026487000	4.956361000
1	1.318175000	0.945677000	5.028165000
1	2.873490000	0.149402000	4.830053000
1	1.647636000	-0.552648000	5.899540000
6	2.041556000	-2.164791000	3.652064000
1	2.043394000	-2.727124000	4.587292000
1	3.078327000	-1.949205000	3.395382000
1	1.627891000	-2.792077000	2.865082000
34	1.572209000	-1.493810000	0.000000000
6	-0.766119000	-2.152159000	4.551703000
1	-0.102277000	-2.911576000	4.935966000
17	3.179048000	1.530794000	-1.771686000
6	-0.309566000	1.148171000	-1.425060000
6	-0.436760000	2.293841000	-3.341068000
1	-0.621390000	2.432207000	-4.388025000
6	-0.019861000	3.155762000	-2.387538000
1	0.256500000	4.191786000	-2.441608000
6	-1.140158000	-0.101539000	-3.408959000
6	-0.229843000	-1.043928000	-3.899425000
6	-2.135133000	-2.297137000	-4.706465000
1	-2.528499000	-3.170293000	-5.209476000
7	0.035131000	2.435259000	-1.208238000
6	-3.005501000	-1.337623000	-4.214331000
1	-4.069956000	-1.473964000	-4.332050000
6	-2.528375000	-0.213665000	-3.547903000
6	-3.484762000	0.848230000	-3.035965000
1	-2.983166000	1.390301000	-2.233524000
6	-3.828446000	1.846148000	-4.153234000
1	-4.350869000	1.339510000	-4.966095000
1	-4.479358000	2.635113000	-3.773369000
1	-2.940581000	2.315240000	-4.575412000
7	-0.622082000	1.068515000	-2.729918000
6	-4.768294000	0.266126000	-2.439544000
1	-4.534773000	-0.468692000	-1.672905000
1	-5.356051000	1.064864000	-1.985047000
1	-5.391841000	-0.200359000	-3.203120000
6	1.268579000	-0.851951000	-3.777767000
1	1.466629000	-0.286073000	-2.871430000
6	1.805500000	-0.026487000	-4.956361000
1	1.318175000	0.945677000	-5.028165000
1	2.873490000	0.149402000	-4.830053000
1	1.647636000	-0.552648000	-5.899540000
6	2.041556000	-2.164791000	-3.652064000
1	2.043394000	-2.727124000	-4.587292000
1	3.078327000	-1.949205000	-3.395382000
1	1.627891000	-2.792077000	-2.865082000
6	-0.766119000	-2.152159000	-4.551703000
1	-0.102277000	-2.911576000	-4.935966000

4

E = -9209.6475767

Free energy = -9209.066882

34	-0.020815000	-1.239937000	-1.479321000
32	0.013986000	0.930685000	-0.394598000
31	-0.038263000	-2.984271000	0.151675000
17	1.724167000	-2.928999000	1.562037000
7	-2.706621000	0.810827000	0.953485000
6	-1.386747000	0.569247000	1.079644000
17	-0.067235000	-4.913491000	-0.897294000
7	-1.202803000	0.279515000	2.386896000
6	-3.352982000	0.645520000	2.165388000
1	-4.411235000	0.787324000	2.267009000
17	-1.788736000	-2.886207000	1.574807000
7	1.214315000	0.246121000	2.387069000
6	-2.409281000	0.301711000	3.068319000
1	-2.487169000	0.049579000	4.109174000
7	2.732703000	0.741090000	0.956558000
6	-0.002023000	-0.297582000	2.961039000
1	-0.017262000	-1.376226000	2.803022000
1	0.000906000	-0.081129000	4.026451000
6	1.406810000	0.533123000	1.080227000
6	3.373298000	0.557642000	2.168678000
1	4.434509000	0.674321000	2.271735000
6	2.419964000	0.236380000	3.069888000
1	2.489827000	-0.018574000	4.110639000
6	-3.344654000	1.254797000	-0.263335000
6	-3.908959000	0.297554000	-1.116326000
6	-4.536082000	0.770701000	-2.267135000
1	-4.976316000	0.064066000	-2.954645000
6	-4.592870000	2.126851000	-2.547274000
1	-5.083393000	2.469482000	-3.448588000
6	-4.016259000	3.046871000	-1.685638000

1	-4.059682000	4.100149000	-1.922650000
6	-3.374510000	2.630040000	-0.522254000
6	-2.764991000	3.645301000	0.425978000
1	-2.091394000	3.117426000	1.099846000
6	-3.854042000	4.305189000	1.283282000
1	-4.556925000	4.855761000	0.656344000
1	-3.412314000	5.006127000	1.993227000
1	-4.421424000	3.563727000	1.846437000
6	-1.925776000	4.695571000	-0.306370000
1	-1.180391000	4.220349000	-0.941706000
1	-1.407708000	5.329785000	0.414012000
1	-2.542904000	5.344450000	-0.928336000
6	-3.872984000	-1.181061000	-0.794145000
1	-3.019427000	-1.362052000	-0.147491000
6	-3.684970000	-2.066521000	-2.026798000
1	-4.570200000	-2.074504000	-2.665602000
1	-3.500147000	-3.091755000	-1.706716000
1	-2.826633000	-1.744861000	-2.612756000
6	-5.131957000	-1.599422000	-0.021298000
1	-5.264321000	-1.015366000	0.889430000
1	-5.057408000	-2.648432000	0.264506000
1	-6.025320000	-1.469598000	-0.635298000
6	3.382231000	1.179190000	-0.256048000
6	3.906426000	0.214768000	-1.126918000
6	4.537450000	0.681748000	-2.277714000
1	4.946885000	-0.029839000	-2.978929000
6	4.638696000	2.039360000	-2.540237000
1	5.131034000	2.377008000	-3.442433000
6	4.107299000	2.966436000	-1.658081000
1	4.188524000	4.021539000	-1.878184000
6	3.463457000	2.555585000	-0.493272000
6	2.899717000	3.582710000	0.469990000
1	2.310401000	3.058582000	1.221077000
6	4.028917000	4.317162000	1.204765000
1	4.672652000	3.618912000	1.740965000
1	3.619560000	5.025841000	1.926302000
1	4.652969000	4.872849000	0.503527000
6	1.958314000	4.565958000	-0.231570000
1	2.491810000	5.188334000	-0.950513000
1	1.493692000	5.230190000	0.498523000
1	1.170900000	4.031801000	-0.760375000
6	3.828649000	-1.264957000	-0.818319000
1	2.970509000	-1.426929000	-0.172689000
6	5.075336000	-1.723545000	-0.047892000
1	5.972372000	-1.612446000	-0.660237000
1	4.972329000	-2.772542000	0.228980000
1	5.222682000	-1.151311000	0.868076000
6	3.618281000	-2.135196000	-2.057746000
1	2.769833000	-1.785896000	-2.642206000
1	3.405475000	-3.157460000	-1.745781000
1	4.503998000	-2.162152000	-2.695397000

5

E = -7076.1723657
Free energy (298.15) = -7075.512761

52	0.095675000	-1.227303000	-1.583986000
32	-0.067845000	1.071108000	-0.309131000
31	0.167365000	-3.027483000	0.272522000
17	1.900362000	-2.771042000	1.702207000
7	2.660435000	1.021068000	1.020956000
6	1.350451000	0.719547000	1.139575000
7	1.175930000	0.411322000	2.444841000
17	0.287040000	-5.019254000	-0.652300000
6	-1.441244000	0.551784000	1.134408000
17	-1.624172000	-2.950868000	1.649206000
7	-1.235138000	0.261290000	2.439294000
6	3.307367000	0.879272000	2.235878000
1	4.357244000	1.068283000	2.344183000
7	-2.776853000	0.700796000	1.014916000
6	2.377344000	0.485509000	3.131585000
1	2.462672000	0.228778000	4.170661000
6	0.004044000	-0.229466000	3.010281000
1	-0.011224000	-0.023686000	4.077705000
1	0.072905000	-1.304661000	2.843884000
6	-2.437689000	0.194433000	3.123923000
1	-2.493851000	-0.073211000	4.162250000
6	-3.405583000	0.481849000	2.227595000
1	-4.470190000	0.553743000	2.334637000
6	3.287141000	1.461668000	-0.202038000
6	3.961112000	0.516561000	-0.986287000
6	4.558208000	0.978795000	-2.157461000
1	5.078838000	0.279346000	-2.794371000
6	4.481174000	2.312979000	-2.522735000
1	4.947469000	2.647079000	-3.439969000
6	3.805038000	3.222450000	-1.724017000
1	3.750907000	4.258270000	-2.024657000
6	3.191146000	2.816820000	-0.541997000
6	2.498037000	3.825020000	0.356211000
1	1.779487000	3.288324000	0.974637000
6	1.707563000	4.874778000	-0.427214000
1	1.124665000	5.489579000	0.259062000
1	1.021568000	4.401615000	-1.127883000
1	2.363383000	5.543563000	-0.985427000
6	3.515703000	4.490901000	1.294185000
1	4.043715000	3.753936000	1.899303000
1	3.016972000	5.188710000	1.968589000
1	4.259439000	5.045543000	0.720208000
6	4.079615000	-0.935244000	-0.571241000
1	3.248890000	-1.166901000	0.090096000
6	3.998866000	-1.910887000	-1.746789000
1	4.893067000	-1.871451000	-2.371644000
1	3.126888000	-1.709090000	-2.364813000
1	3.907644000	-2.927279000	-1.364626000
6	5.377390000	-1.170448000	0.217275000
1	6.250486000	-0.986061000	-0.411531000

1	5.415776000	-2.202660000	0.565595000	1	-4.929915000	-2.859922000	0.346969000
1	5.453558000	-0.521761000	1.089448000	6	-3.673515000	-2.212078000	-1.983635000
6	-3.447654000	1.119983000	-0.191818000	1	-4.584825000	-2.255643000	-2.583144000
6	-3.565577000	2.493593000	-0.430777000	1	-3.434358000	-3.229270000	-1.674343000
6	-4.224287000	2.885601000	-1.593902000	1	-2.855337000	-1.855286000	-2.605940000
1	-4.333883000	3.937794000	-1.815139000	6	-3.040019000	3.536690000	0.537295000
6	-4.737590000	1.943616000	-2.470634000	1	-2.418311000	3.033951000	1.276695000
1	-5.242289000	2.266723000	-3.371321000	6	-2.155234000	4.574981000	-0.158180000
6	-4.605421000	0.589382000	-2.203568000	1	-1.343275000	4.088991000	-0.696166000
1	-5.003867000	-0.132699000	-2.900131000	1	-1.721564000	5.253465000	0.577853000
6	-3.956815000	0.140426000	-1.055422000	1	-2.723306000	5.178435000	-0.866774000
6	-3.845905000	-1.337219000	-0.741429000	6	-4.197545000	4.205053000	1.291347000
1	-2.961286000	-1.483530000	-0.126635000	1	-4.853251000	4.739675000	0.602626000
6	-5.058233000	-1.812963000	0.072993000	1	-3.816814000	4.922432000	2.020040000
1	-5.976693000	-1.716625000	-0.509246000	1	-4.802497000	3.469166000	1.822221000
1	-5.184059000	-1.243261000	0.992967000				

(b) Compounds 3-5 calculated at ZORA-BP86-D3(BJ)/def2-TVZP(-f)

3

E = -11772.7600822
Free energy= -11772.1929191

Se	-2.48547000	-1.28132200	0.00109500	C	1.28835700	-0.86759500	3.69235500
Ge	-0.52347300	-0.23382500	0.00038300	H	1.48289500	-0.28911400	2.77900700
Ga	3.19744800	0.15333500	-0.00224200	C	1.80041000	-0.03224900	4.88000700
Cl	3.09011100	1.55014900	1.76258400	H	1.29688500	0.94147000	4.94194900
C	-0.33750900	1.19301200	1.41906300	H	2.87658500	0.15833300	4.76634100
Cl	5.10223400	-0.93076600	-0.00348800	H	1.63449700	-0.56340100	5.82967700
C	-0.42020400	2.31619500	3.36520600	C	2.08921600	-2.16574700	3.57179900
H	-0.58277300	2.44197900	4.42679300	H	2.09871500	-2.73161800	4.51544500
C	-0.02745800	3.19957600	2.40537500	H	3.13034300	-1.92488800	3.31740500
H	0.25426900	4.24285500	2.46777100	H	1.69276300	-2.80665400	2.77352400
C	0.60910600	3.01861900	-0.00189900	Se	1.45945700	-1.49724300	0.00021100
H	0.48536200	4.10762200	-0.00238200	C	-0.74550800	-2.24122600	4.36960500
H	1.68113900	2.75961000	-0.00253300	H	-0.07112500	-3.03464300	4.68875200
C	-1.12801700	-0.10130300	3.38279600	Cl	3.08828600	1.54819700	-1.76817500
C	-0.20896400	-1.08009900	3.80075900	C	-0.33873600	1.19147000	-1.42011800
C	-2.12107900	-2.39841300	4.52067300	C	-0.42601700	2.31361100	-3.36670200
H	-2.51616800	-3.31671500	4.95755000	H	-0.59085000	2.43865600	-4.42802900
N	-0.00425000	2.49422500	1.20971200	C	-0.03201200	3.19776500	-2.40810800
C	-2.99772700	-1.39684700	4.11197400	H	0.24875100	4.24124600	-2.47159000
H	-4.07193300	-1.54157900	4.22348400	C	-1.13200800	-0.10432100	-3.38189500
C	-2.52163700	-0.22114900	3.52262900	C	-0.21325900	-1.08370800	-3.79921600
C	-3.47465900	0.88297600	3.09566800	C	-2.12590900	-2.40258500	-4.51677600
H	-2.97314600	1.47306300	2.31307000	H	-2.52128800	-3.32135000	-4.95241500
C	-3.78506700	1.81015100	4.28645600	N	-0.00599600	2.49306600	-1.21211900
H	-4.30846100	1.25116800	5.07623600	C	-3.00221000	-1.40033000	-4.10911300
H	-4.43246700	2.64068300	3.96927000	H	-4.07648500	-1.54493000	-4.22016700
H	-2.87540600	2.23579300	4.73128800	C	-2.52565500	-0.22407500	-3.52120900
N	-0.61722600	1.09494800	2.73911300	C	-3.47847700	0.88062700	-3.09528800
C	-4.77787300	0.35202200	2.48806700	H	-2.97584300	1.47307700	-2.31521400
H	-4.55993000	-0.32662700	1.65333100	C	-3.79142200	1.80445200	-4.28803700
H	-5.38039900	1.19131800	2.11119200	H	-4.31579000	1.24300300	-5.07541700
H	-5.38703100	-0.17840500	3.23473200	H	-4.43878700	2.63548300	-3.97208600

H	-2.88271800	2.22927100	-4.73561200
N	-0.62090100	1.09252700	-2.73958300
C	-4.78040000	0.35058000	-2.48410700
H	-4.56077600	-0.32574500	-1.64792500
H	-5.38286000	1.19054600	-2.10862600
H	-5.39031400	-0.18233900	-3.22836700
C	1.28414900	-0.87105300	-3.69234400
H	1.47956000	-0.29194700	-2.77959300
C	1.79488800	-0.03650200	-4.88111000
H	1.29149100	0.93728800	-4.94297800
H	2.87123700	0.15396900	-4.76888700
H	1.62771500	-0.56819300	-5.83025700
C	2.08527200	-2.16902100	-3.57164600
H	2.09383800	-2.73558600	-4.51488900
H	3.12663600	-1.92783100	-3.31854200
H	1.68976500	-2.80940000	-2.77246700
C	-0.75024000	-2.24545100	-4.36634800
H	-0.07614000	-3.03935900	-4.68485100

4

E = -9326.5215626
Free energy = -9325.9558887

Se	-0.03559900	-1.17855700	-1.44943900
Ge	0.02886100	0.99800900	-0.35379300
Ga	-0.05168500	-2.89722400	0.19899600
Cl	1.71981600	-2.86478400	1.60535000
N	-2.69830500	0.82826600	0.96121900
C	-1.36733200	0.60871600	1.11122900
Cl	-0.09679400	-4.82553400	-0.86553200
N	-1.20443000	0.32378200	2.43237700
C	-3.36842900	0.65080100	2.16356800
H	-4.44004300	0.77386900	2.24623600
Cl	-1.79246000	-2.81647500	1.64489000
N	1.21668500	0.26952800	2.44173300
C	-2.42907900	0.31985400	3.09159200
H	-2.52507100	0.05488200	4.13698300
N	2.74202900	0.72918000	0.99001400
C	-0.00853200	-0.26543600	3.01646300
H	-0.03361200	-1.35623700	2.84941900
H	-0.00771800	-0.04720800	4.09147000
C	1.40240300	0.55663600	1.12382900
C	3.39344800	0.51935200	2.19689700
H	4.46726700	0.61281600	2.29175900
C	2.43296900	0.21724500	3.11327300
H	2.50750400	-0.05573100	4.15831400
C	-3.29937200	1.25238500	-0.28467300
C	-3.82034300	0.27128800	-1.14868800
C	-4.39253700	0.72236000	-2.34453300
H	-4.79399500	-0.00809100	-3.04612600
C	-4.43523600	2.07934900	-2.65565600
H	-4.88164600	2.40720600	-3.59551600
C	-3.89612700	3.02184100	-1.78244600
H	-3.92234500	4.07897000	-2.04659800
C	-3.30732300	2.62812100	-0.57527900
C	-2.73096700	3.65515200	0.38459900
H	-2.01684200	3.13578400	1.04109600
C	-3.84092200	4.24161800	1.27303400
H	-4.59208800	4.76128600	0.66006500
H	-3.42562700	4.96388400	1.99180600
H	-4.35755700	3.45473300	1.84012200
C	-1.95395200	4.76108100	-0.33916800
H	-1.19258000	4.33257600	-1.00519700
H	-1.44661200	5.40664000	0.39142900
H	-2.61634300	5.40120900	-0.94012800
C	-3.78274000	-1.20044300	-0.79513600
H	-2.91825000	-1.36163800	-0.13771200
C	-3.59396200	-2.11371900	-2.00902700
H	-4.49754400	-2.16038500	-2.63637300
H	-3.37550200	-3.13329100	-1.66376800
H	-2.74527400	-1.78500400	-2.62277500
C	-5.04182500	-1.60798900	-0.00897500
H	-5.17672600	-0.99956100	0.89489500
H	-4.95826000	-2.65778000	0.30404800
H	-5.94328500	-1.49703100	-0.63113400
C	3.36743000	1.17930900	-0.23421900
C	3.81490200	0.21621700	-1.15858900
C	4.39209500	0.69746800	-2.33961300
H	4.73684100	-0.01580100	-3.08745200
C	4.51494800	2.06558300	-2.57762100
H	4.96377000	2.41584200	-3.50829100
C	4.06000200	2.98942700	-1.64014200
H	4.15354100	4.05753700	-1.84188600
C	3.47003800	2.56460600	-0.44323300
C	2.96470000	3.57944100	0.56774200
H	2.49351300	3.03043000	1.39542700
C	4.12496000	4.39478400	1.15828500
H	4.87789500	3.74031500	1.62052200
H	3.75783400	5.09288800	1.92520900
H	4.62907900	4.98570300	0.37916700
C	1.89003500	4.48635600	-0.04747100
H	2.30525600	5.11018200	-0.85272200
H	1.46844900	5.15721400	0.71501100
H	1.07415400	3.88411100	-0.47029000
C	3.69930400	-1.26649400	-0.87397400
H	2.81616600	-1.41216900	-0.23791500
C	4.92212800	-1.76759600	-0.08521100
H	5.84061300	-1.66809700	-0.68395400
H	4.78437300	-2.82573800	0.17593000
H	5.06419100	-1.21046900	0.85042100
C	3.48922100	-2.11600700	-2.12929100
H	2.66353200	-1.72438000	-2.73742600
H	3.22196000	-3.13905100	-1.83172900

H	4.39870800	-2.17491900	-2.7468250

5

E = -13774.649943
Free energy = -13774.083655

Te	0.07900500	-1.10016400	-1.54624400
Ge	-0.05977800	1.19866500	-0.24648200
Ga	0.16077900	-2.88269200	0.31500100
Cl	1.89928700	-2.65139600	1.75137000
N	2.66256400	1.01083900	1.06768500
C	1.33296100	0.76466500	1.19800400
N	1.15959000	0.46258100	2.51495200
Cl	0.28601200	-4.86765300	-0.64025500
C	-1.43267800	0.61489900	1.16309100
Cl	-1.61824900	-2.85329300	1.71820600
N	-1.25820800	0.33615100	2.48535100
C	3.31945100	0.83893400	2.27821100
H	4.38607000	0.98794100	2.37816600
N	-2.77746800	0.72662600	1.00391900
C	2.37562700	0.47874300	3.18982100
H	2.46352300	0.20211500	4.23279100
C	-0.02702900	-0.15888900	3.08229600
H	-0.05227300	0.06509400	4.15596700
H	0.03184400	-1.24958100	2.92290400
C	-2.48382600	0.23410400	3.13505800
H	-2.56575300	-0.04489000	4.17788000
C	-3.43909000	0.49496700	2.20199500
H	-4.51732200	0.53653400	2.27784400
C	3.26328800	1.47306400	-0.16353000
C	3.85750300	0.52863000	-1.02162200
C	4.41297100	1.01875200	-2.20984700
H	4.86879800	0.31808800	-2.90846600
C	4.36946100	2.37615400	-2.52020500
H	4.80174900	2.73244400	-3.45631300
C	3.76685100	3.28178700	-1.64967000
H	3.73261200	4.33949700	-1.91075000
C	3.19511900	2.84875200	-0.44773400
C	2.57257100	3.83819700	0.52252800
H	1.88903300	3.28095600	1.17973500
C	1.73358300	4.90895600	-0.18325500
H	1.20693800	5.52539700	0.55900800
H	0.98345100	4.44553200	-0.83820400
H	2.35297600	5.58553400	-0.79016500
C	3.65870900	4.47013900	1.40951100
H	4.22092100	3.70390800	1.96210200
H	3.21139100	5.15879300	2.14182300
H	4.37679500	5.03749300	0.79911700
C	3.91647200	-0.94362200	-0.66790600
H	3.04277800	-1.16944600	-0.04011400
C	3.84746200	-1.86746000	-1.88713600
H	4.77843400	-1.84353800	-2.47416400
H	3.00398700	-1.60180600	-2.53712700
H	3.69265100	-2.90127000	-1.54957700
C	5.17522700	-1.25995400	0.16079500
H	6.08545400	-1.08557500	-0.43342800
H	5.15758800	-2.31341200	0.47203300
H	5.24067300	-0.64543100	1.06758600
C	-3.40509300	1.10158400	-0.24368800
C	-3.49000300	2.47054400	-0.55216500
C	-4.10497200	2.81589800	-1.76133100
H	-4.19041700	3.86686100	-2.03717600
C	-4.59791500	1.83430400	-2.61797100
H	-5.06695100	2.12459900	-3.55913500
C	-4.48328400	0.48581900	-2.28765500
H	-4.85131800	-0.27406100	-2.97597900
C	-3.88055600	0.08200700	-1.09029900
C	-3.76250900	-1.38162400	-0.71799000
H	-2.87111500	-1.49051100	-0.08492500
C	-4.97876200	-1.84370200	0.10502700
H	-5.89994200	-1.78296300	-0.49461400
H	-5.12235000	-1.23728800	1.00829200
H	-4.83493200	-2.88551600	0.42244200
C	-3.56981000	-2.30296200	-1.92557400
H	-4.48955700	-2.39890400	-2.52293100
H	-3.29634500	-3.30670600	-1.57325000
H	-2.75687800	-1.94567300	-2.57113700
C	-2.97284600	3.54125300	0.39394500
H	-2.23726900	3.06932600	1.06220500
C	-2.24578700	4.67093500	-0.34468600
H	-1.45383500	4.26628400	-0.98901200
H	-1.78398400	5.35874900	0.37748600
H	-2.93284200	5.26029000	-0.96954400
C	-4.11872900	4.08356900	1.26424700
H	-4.89364400	4.54808600	0.63676200
H	-3.74919800	4.84240700	1.97019700
H	-4.59417600	3.27995500	1.84370400

(c) Compounds 1‘-5‘ (R=Ph) optimized at B3LYP-D3(BJ)/def2-SV

1‘

E = -6333.9048153

Free Energy = -6333.646585

32	-0.000040000	0.665365000	0.581496000
31	0.000028000	-0.347321000	-1.757977000
17	-0.000488000	1.116373000	-3.420943000
7	2.714342000	-0.537122000	1.260691000
6	1.387090000	-0.750919000	1.085090000
17	1.796331000	-1.738690000	-1.969525000
7	1.201458000	-2.066868000	1.374829000
6	-1.387149000	-0.750934000	1.085080000
17	-1.795618000	-1.739561000	-1.969396000
7	-1.201494000	-2.066870000	1.374866000
6	3.362152000	-1.711636000	1.624949000
1	4.435977000	-1.745511000	1.787912000
7	-2.714407000	-0.537151000	1.260672000
6	2.409211000	-2.678493000	1.692849000
1	2.482094000	-3.738130000	1.925977000
6	-0.000023000	-2.792766000	1.009655000
1	-0.000065000	-2.955996000	-0.083591000
1	-0.000012000	-3.758717000	1.533788000
6	-2.409243000	-2.678506000	1.692885000
1	-2.482111000	-3.738136000	1.926045000
6	-3.362200000	-1.711668000	1.624959000
1	-4.436025000	-1.745556000	1.787927000
6	3.360649000	0.712035000	0.980331000
6	3.398417000	1.165269000	-0.341203000
6	3.997322000	2.396801000	-0.616453000
1	4.019962000	2.762906000	-1.646353000
6	4.563258000	3.150694000	0.416571000
1	5.031070000	4.114568000	0.196098000
6	4.531514000	2.677335000	1.732908000
1	4.970590000	3.269017000	2.541163000
6	3.922211000	1.454038000	2.022037000
6	-3.360704000	0.712005000	0.980289000
6	-3.398415000	1.165265000	-0.341237000
6	-3.997296000	2.396806000	-0.616497000
1	-4.019873000	2.762929000	-1.646392000
6	-4.563280000	3.150682000	0.416513000
1	-5.031075000	4.114563000	0.196035000
6	-4.531603000	2.677297000	1.732842000
1	-4.970717000	3.268965000	2.541087000
6	-3.922316000	1.453995000	2.021978000
1	-3.863977000	1.081300000	3.047866000
1	-2.974318000	0.542972000	-1.133552000
1	3.863823000	1.081359000	3.047928000
1	2.974356000	0.542943000	-1.133517000

2‘

E = -7130.168278
Free energy = -7129.906940

32	-0.107481000	-1.070965000	-0.014949000
31	0.248294000	2.334671000	-0.756320000
17	1.983792000	2.432813000	0.683850000
16	-0.334332000	-3.147194000	-0.117485000
7	-1.270984000	0.108754000	2.519427000
6	-1.495591000	-0.346606000	1.263931000
17	0.428089000	3.889441000	-2.279457000
16	0.108069000	0.286650000	-1.748958000
7	-2.826146000	-0.500207000	1.154545000
6	-3.457409000	-0.107383000	2.323658000
1	-4.538680000	-0.138432000	2.427315000
6	-2.477839000	0.283984000	3.185957000
1	-2.531426000	0.675715000	4.198785000
6	-0.016865000	0.678405000	2.981309000
1	0.077700000	1.700735000	2.573803000
1	-0.036066000	0.707704000	4.079545000
6	-3.473209000	-0.938558000	-0.054410000
6	-3.540310000	-0.052102000	-1.130491000
6	-4.636187000	-1.786247000	-2.418038000
1	-5.088891000	-2.124580000	-3.354570000
6	-4.127558000	-0.487729000	-2.320626000
1	-4.176573000	0.190991000	-3.176116000
6	-4.554821000	-2.662134000	-1.329844000
1	-4.931776000	-3.684738000	-1.416421000
6	-3.961684000	-2.242997000	-0.138249000
17	-1.545577000	2.708987000	0.560565000
7	1.146397000	-0.090020000	2.565855000
6	1.329807000	-0.598922000	1.324846000
7	2.631227000	-0.921180000	1.239865000
6	3.290771000	-0.582985000	2.410125000
1	4.357031000	-0.752210000	2.533530000
6	2.356687000	-0.052621000	3.248090000
1	2.445208000	0.352415000	4.253189000
6	3.248344000	-1.417825000	0.034822000
6	3.713600000	-0.489486000	-0.898819000
6	4.376365000	-2.334613000	-2.319498000
1	4.816365000	-2.697849000	-3.252802000
6	4.285914000	-0.959157000	-2.082469000
1	4.645638000	-0.245089000	-2.828015000
6	3.889172000	-3.248528000	-1.379632000
1	3.937373000	-4.322397000	-1.578577000
6	3.313908000	-2.792723000	-0.191498000
1	3.597195000	0.578103000	-0.699909000
1	2.885177000	-3.485813000	0.533713000
1	-3.841715000	-2.924735000	0.705885000
1	-3.123220000	0.951811000	-1.029065000

3'

E = -11136.606975

Free Energy = -11136.348451

34	0.404067000	-3.165783000	-0.049457000
32	0.118906000	-0.955823000	0.058765000
31	-0.322620000	2.569872000	-0.556607000
17	1.464798000	2.889647000	0.785069000
6	1.486922000	-0.219719000	1.350939000
17	-0.541259000	4.234603000	-1.958117000
6	3.442477000	0.081067000	2.407235000
1	4.524631000	0.094602000	2.504907000
6	2.451751000	0.408266000	3.282884000
1	2.493431000	0.778472000	4.304293000
6	-0.019099000	0.715189000	3.098313000
1	-0.000677000	0.704294000	4.196950000
1	-0.139885000	1.749194000	2.728691000
6	3.491290000	-0.675798000	0.009508000
6	3.545646000	0.261394000	-1.023550000
6	4.714959000	-1.375969000	-2.371853000
1	5.192051000	-1.655874000	-3.315553000
7	1.250213000	0.197407000	2.617667000
6	4.645759000	-2.303744000	-1.326708000
1	5.056514000	-3.308891000	-1.453903000
6	4.023230000	-1.958720000	-0.126079000
7	2.823406000	-0.310190000	1.231434000
34	-0.124297000	0.501094000	-1.765833000
6	4.164355000	-0.099593000	-2.222687000
1	4.204665000	0.619672000	-3.044846000
17	-2.061124000	2.517510000	0.883470000
6	-1.335179000	-0.538833000	1.397160000
6	-3.295342000	-0.599154000	2.483857000
1	-4.357989000	-0.793362000	2.601195000
6	-2.371512000	-0.078694000	3.338669000
1	-2.467029000	0.290925000	4.356662000
6	-3.247966000	-1.350444000	0.085291000
6	-3.698419000	-0.400368000	-0.833645000
6	-4.387337000	-2.212076000	-2.284736000
1	-4.831745000	-2.553419000	-3.224204000
7	-1.162004000	-0.066552000	2.654657000
6	-3.917632000	-3.148494000	-1.358123000
1	-3.983894000	-4.218260000	-1.573265000
6	-3.337573000	-2.720511000	-0.161941000
7	-2.631463000	-0.882319000	1.301635000
6	-4.276064000	-0.842249000	-2.025475000
1	-4.624734000	-0.110678000	-2.759155000
1	-2.926085000	-3.432097000	0.555484000
1	-3.570180000	0.662008000	-0.615415000
1	3.915603000	-2.680587000	0.685508000
1	3.095651000	1.246077000	-0.882401000

4'

E = -8735.2675094

Free energy = -8735.006468

34	0.027010000	0.116969000	-1.723682000
32	-0.020580000	-1.517441000	0.081153000
31	0.037666000	2.228324000	-0.622854000
17	-1.758503000	2.448539000	0.739496000
7	2.716426000	-0.904228000	1.273099000
6	1.382059000	-0.715400000	1.376045000
17	0.063738000	3.889456000	-2.061603000
7	1.194081000	-0.173210000	2.606864000
6	3.371858000	-0.443882000	2.404669000
1	4.454006000	-0.482655000	2.494320000
17	1.816919000	2.416474000	0.766359000
7	-1.227481000	-0.149329000	2.596228000
6	2.411766000	0.021644000	3.247378000
1	2.485468000	0.490951000	4.225371000
7	-2.756441000	-0.848675000	1.252639000
6	-0.012017000	0.508964000	3.038417000
1	0.000021000	1.535898000	2.630601000
1	-0.016779000	0.538278000	4.137778000
6	-1.419278000	-0.681288000	1.361204000
6	-3.408437000	-0.385429000	2.385190000
1	-4.491418000	-0.406935000	2.470766000
6	-2.444157000	0.060337000	3.233737000
1	-2.514116000	0.524372000	4.214533000
6	3.365444000	-1.398111000	0.089545000
6	3.521284000	-0.532525000	-0.995300000
6	4.139508000	-1.011332000	-2.152656000
1	4.256326000	-0.346876000	-3.012716000
6	4.591742000	-2.332976000	-2.214156000
1	5.070883000	-2.703051000	-3.125261000
6	4.428124000	-3.188154000	-1.118605000
1	4.775715000	-4.223687000	-1.170737000
6	3.808220000	-2.721725000	0.042896000
6	-3.410516000	-1.329037000	0.066944000
6	-3.479089000	-0.486669000	-1.044761000
6	-4.102946000	-0.954060000	-2.203873000
1	-4.150888000	-0.308168000	-3.084433000
6	-4.651017000	-2.239833000	-2.239279000
1	-5.135366000	-2.600933000	-3.151223000
6	-4.578241000	-3.070633000	-1.115352000
1	-5.001557000	-4.078473000	-1.146380000
6	-3.951211000	-2.616879000	0.047198000
1	-3.866745000	-3.255597000	0.930119000
1	-3.038892000	0.509998000	-0.989131000

1	3.149209000	0.490611000	-0.920848000
1	3.655417000	-3.376578000	0.904449000

5

E = -6602.0770578
Free energy = -6601.819376

52	0.000088000	-0.096671000	-1.762457000
32	0.000077000	-1.625067000	0.407836000
31	-0.000154000	2.239323000	-0.661440000
17	-1.785631000	2.497288000	0.715412000
7	-2.732929000	-0.773066000	1.427798000
6	-1.393646000	-0.638139000	1.555767000
7	-1.213522000	0.025774000	2.727608000
17	-0.000267000	3.862485000	-2.147588000
6	1.393754000	-0.638091000	1.555789000
17	1.785225000	2.497569000	0.715512000
7	1.213590000	0.025828000	2.727621000
6	-3.396189000	-0.165023000	2.482166000
1	-4.480960000	-0.146238000	2.540014000
7	2.733043000	-0.772927000	1.427810000
6	-2.438949000	0.342150000	3.302920000
1	-2.517125000	0.918348000	4.221538000
6	0.000015000	0.715078000	3.126325000
1	0.000004000	0.806761000	4.222885000
1	0.000008000	1.717767000	2.660584000
6	2.439001000	0.342296000	3.302917000
1	2.517143000	0.918517000	4.221524000
6	3.396272000	-0.164831000	2.482171000
1	4.481043000	-0.145972000	2.540005000
6	-3.371863000	-1.339587000	0.271223000
6	-3.646129000	-0.501019000	-0.811570000
6	-4.256823000	-1.044047000	-1.943877000
1	-4.468398000	-0.400501000	-2.801742000
6	-4.572618000	-2.405502000	-1.988085000
1	-5.042875000	-2.826664000	-2.881455000
6	-4.280335000	-3.235131000	-0.900044000
1	-4.520087000	-4.301299000	-0.939743000
6	-3.674908000	-2.701810000	0.240647000
6	3.372003000	-1.339403000	0.271227000
6	3.675001000	-2.701635000	0.240564000
6	4.280431000	-3.234895000	-0.900151000
1	4.520139000	-4.301072000	-0.939920000
6	4.572780000	-2.405202000	-1.988128000
1	5.043046000	-2.826325000	-2.881511000
6	4.257041000	-1.043738000	-1.943834000
1	4.468665000	-0.400138000	-2.801645000
6	3.646335000	-0.500773000	-0.811504000
1	-3.429516000	-3.332433000	1.098595000
1	-3.360384000	0.551539000	-0.757892000
1	3.360607000	0.551786000	-0.757758000
1	3.429554000	-3.332310000	1.098459000

C (S)

E = -6732.0180601
Free energy = -6731.760768

32	-0.054344000	0.499191000	0.019988000
31	0.232588000	-1.426885000	-1.513339000
17	0.428927000	-1.169256000	-3.678472000
7	2.586650000	0.262904000	1.562407000
6	1.315847000	-0.151097000	1.416370000
17	2.008567000	-2.532360000	-0.623383000
7	1.137780000	-1.128177000	2.334857000
6	-1.485582000	-0.353617000	1.244523000
17	-1.535610000	-2.734419000	-0.954155000
7	-1.261318000	-1.259853000	2.224745000
6	3.228708000	-0.470528000	2.550239000
1	4.268630000	-0.291338000	2.810104000
7	-2.788096000	-0.029507000	1.338562000
6	2.314590000	-1.355279000	3.038182000
1	2.395926000	-2.114223000	3.812791000
6	-0.021877000	-2.007075000	2.345683000
1	0.056941000	-2.712241000	1.499952000
1	-0.035651000	-2.558790000	3.295023000
6	-2.431798000	-1.520044000	2.927781000
1	-2.476410000	-2.233598000	3.747228000
6	-3.395840000	-0.737579000	2.367137000
1	-4.453710000	-0.626632000	2.590436000
6	3.209447000	1.218343000	0.680737000
6	3.732223000	0.753995000	-0.528029000
6	4.289306000	1.676361000	-1.417060000
1	4.690601000	1.329417000	-2.373029000
6	4.316158000	3.035666000	-1.090269000
1	4.744205000	3.756095000	-1.793276000
6	3.778769000	3.482292000	0.121114000
1	3.775079000	4.548768000	0.361388000
6	3.212703000	2.571714000	1.014535000
6	-3.447946000	0.864557000	0.427158000
6	-3.501051000	0.513907000	-0.922804000
6	-4.083129000	1.405400000	-1.825857000
1	-4.117667000	1.149366000	-2.888118000
6	-4.610813000	2.618317000	-1.373748000
1	-5.058990000	3.317131000	-2.085806000
6	-4.557839000	2.948033000	-0.015381000
1	-4.955716000	3.904660000	0.334239000
6	-3.966586000	2.071177000	0.895109000

1	-3.871488000	2.333805000	1.951293000
1	-3.084704000	-0.442878000	-1.249607000
1	2.743089000	2.904819000	1.941497000
1	3.668542000	-0.310004000	-0.769479000
16	-0.261798000	2.588297000	0.0678070

C(Se)

E = -8735.2399658
Free energy = -8734.983567

32	-0.000164000	0.306674000	0.023004000
31	0.000758000	-1.736825000	1.428066000
17	0.001532000	-1.624249000	3.614470000
7	-2.691356000	0.064678000	-1.450602000
6	-1.411144000	-0.334587000	-1.338738000
17	-1.784836000	-2.880669000	0.623758000
7	-1.201118000	-1.187592000	-2.368377000
6	1.410699000	-0.333953000	-1.339155000
17	1.786229000	-2.880085000	0.622699000
7	1.200794000	-1.187106000	-2.368701000
6	-3.300019000	-0.544468000	-2.539613000
1	-4.342227000	-0.361345000	-2.787528000
7	2.690793000	0.065632000	-1.451159000
6	-2.359705000	-1.341284000	-3.120212000
1	-2.413562000	-1.998286000	-3.985129000
6	-0.000021000	-1.996271000	-2.492524000
1	0.000238000	-2.762974000	-1.699020000
1	-0.000062000	-2.477277000	-3.479473000
6	2.359371000	-1.340602000	-3.120595000
1	2.413324000	-1.997685000	-3.985446000
6	3.299524000	-0.543481000	-2.540155000
1	4.341643000	-0.360051000	-2.788215000
6	-3.337983000	0.926264000	-0.497941000
6	-3.517145000	0.456750000	0.804376000
6	-4.085499000	1.308994000	1.753235000
1	-4.217952000	0.958689000	2.780292000
6	-4.473803000	2.602174000	1.391403000
1	-4.910847000	3.269457000	2.139720000
6	-4.294594000	3.051962000	0.079061000
1	-4.581996000	4.070070000	-0.197320000
6	-3.715976000	2.214197000	-0.875122000
6	3.337315000	0.927383000	-0.498560000
6	3.517194000	0.457726000	0.803603000
6	4.085468000	1.310088000	1.752415000
1	4.218466000	0.959654000	2.779358000
6	4.472996000	2.603520000	1.390676000
1	4.909991000	3.270887000	2.138946000
6	4.293062000	3.053462000	0.078479000
1	4.579853000	4.071765000	-0.197816000
6	3.714508000	2.215585000	-0.875639000
1	3.517865000	2.566759000	-1.891026000
1	3.206544000	-0.558871000	1.059154000
1	-3.519896000	2.565295000	-1.890646000
1	-3.205887000	-0.559649000	1.059982000
34	-0.000669000	2.543135000	0.114240000

TS1 C(S)→D(S)

E = -6731.9728586
Free energy = -6731.713079

32	0.037846000	0.183431000	-0.204440000
31	-0.186963000	-2.325906000	-0.812032000
17	-0.498925000	-4.145036000	0.375824000
7	-2.544019000	1.987834000	-0.132507000
6	-1.294752000	1.697085000	-0.577475000
17	-1.993014000	-2.080588000	-2.132339000
7	-1.057992000	2.580985000	-1.587477000
6	1.507612000	1.556115000	-0.608208000
17	1.754844000	-2.516127000	-1.915390000
7	1.338927000	2.453810000	-1.620894000
6	-3.073798000	3.054824000	-0.852249000
1	-4.074635000	3.432528000	-0.657118000
7	2.792678000	1.717584000	-0.197846000
6	-2.140659000	3.430726000	-1.767762000
1	-2.161044000	4.205581000	-2.531420000
6	0.127485000	2.488152000	-2.419340000
1	0.067960000	1.552302000	-3.008793000
1	0.164199000	3.354492000	-3.093526000
6	2.500080000	3.182631000	-1.837313000
1	2.582097000	3.945060000	-2.609329000
6	3.412343000	2.717249000	-0.942368000
1	4.452380000	2.988546000	-0.777857000
6	-3.261458000	1.301924000	0.912504000
6	-3.520019000	-0.065984000	0.790615000
6	-4.232363000	-0.711290000	1.804885000
1	-4.423368000	-1.784583000	1.720573000
6	-4.686693000	0.004332000	2.916362000
1	-5.240729000	-0.508991000	3.707957000
6	-4.428327000	1.375722000	3.022021000
1	-4.774864000	1.937039000	3.894806000
6	-3.709459000	2.030825000	2.019688000
6	3.459031000	0.980727000	0.845550000
6	3.530225000	-0.413307000	0.776418000
6	4.199370000	-1.105561000	1.789342000

1	4.245815000	-2.197071000	1.744763000
6	4.794438000	-0.413546000	2.847942000
1	5.313892000	-0.963983000	3.638042000
6	4.721235000	0.982991000	2.902137000
1	5.177410000	1.528209000	3.733747000
6	4.048403000	1.686547000	1.900907000
1	3.962651000	2.776001000	1.948052000
1	3.081443000	-0.957811000	-0.059073000
1	-3.478795000	3.096637000	2.105023000
1	-3.179609000	-0.622145000	-0.087550000
16	-0.001419000	-0.675077000	1.756141000

TS1 C(Se) →4'(Se)

E = -8735.1950011
Free energy = -8734.936506

32	0.205536000	0.360603000	-0.261224000
31	-0.754384000	-1.823926000	-1.206103000
17	-2.174829000	-3.290537000	-0.380997000
7	-2.102341000	2.341767000	0.273850000
6	-0.898067000	2.048907000	-0.269167000
17	-1.951189000	-0.763977000	-2.811862000
7	-0.537860000	3.156673000	-0.972394000
6	1.834167000	1.536729000	-0.434983000
17	1.075461000	-2.801821000	-1.998293000
7	1.811042000	2.710008000	-1.131326000
6	-2.489552000	3.630408000	-0.074454000
1	-3.436647000	4.047244000	0.258522000
7	3.130129000	1.356314000	-0.075738000
6	-1.505563000	4.148075000	-0.861110000
1	-1.417208000	5.112571000	-1.355981000
6	0.626571000	3.161969000	-1.835581000
1	0.434007000	2.475268000	-2.682147000
1	0.795758000	4.180400000	-2.209147000
6	3.080830000	3.270199000	-1.192978000
1	3.284981000	4.204431000	-1.711262000
6	3.908017000	2.417631000	-0.528192000
1	4.980192000	2.452135000	-0.353268000
6	-2.887469000	1.430031000	1.063919000
6	-3.349995000	0.255400000	0.471474000
6	-4.055390000	-0.662131000	1.251769000
1	-4.374154000	-1.604303000	0.801571000
6	-4.304035000	-0.388991000	2.598663000
1	-4.845677000	-1.115742000	3.210762000
6	-3.849962000	0.803354000	3.174185000
1	-4.035235000	1.009387000	4.232159000
6	-3.130779000	1.721044000	2.407290000
6	3.638053000	0.212086000	0.624987000
6	3.302239000	-1.063850000	0.171000000
6	3.776748000	-2.177997000	0.864003000
1	3.488444000	-3.172526000	0.514633000
6	4.587608000	-2.013045000	1.989060000
1	4.953985000	-2.888191000	2.533174000
6	4.925585000	-0.727309000	2.429118000
1	5.551383000	-0.595943000	3.316343000
6	4.446176000	0.394670000	1.751101000
1	4.673360000	1.402833000	2.108134000
1	2.681955000	-1.207996000	-0.716326000
1	-2.730619000	2.636087000	2.851727000
1	-3.146410000	0.052118000	-0.583130000
34	-0.006700000	-0.893061000	1.632965000

D (S) = 4'(X=S)

E = -6732.0488923
Free Energy = -6731.78603

32	-0.000445000	-1.428241000	-0.112180000
31	0.000449000	2.217573000	-0.692227000
17	-1.787624000	2.420580000	0.678238000
7	2.740440000	-0.895553000	1.139640000
6	1.407389000	-0.686759000	1.230653000
17	0.001454000	3.865164000	-2.142683000
7	1.209247000	-0.204900000	2.484447000
6	3.384256000	-0.505848000	2.304590000
1	4.464133000	-0.566032000	2.409299000
17	1.788125000	2.420287000	0.678808000
7	-1.209827000	-0.204560000	2.484389000
6	2.418276000	-0.065070000	3.154037000
1	2.483729000	0.350813000	4.156549000
7	-2.741142000	-0.894709000	1.139394000
6	-0.000242000	0.461755000	2.930289000
1	-0.000039000	1.493996000	2.536593000
1	-0.000238000	0.475894000	4.029594000
6	-1.408045000	-0.686118000	1.230497000
6	-3.384934000	-0.504995000	2.304377000
1	-4.464815000	-0.565261000	2.409027000
6	-2.418903000	-0.064565000	3.153938000
1	-2.484365000	0.351215000	4.156472000
6	3.404310000	-1.353839000	-0.048225000
6	3.404028000	-0.531238000	-1.176762000
6	4.038036000	-0.981792000	-2.337021000
1	4.030305000	-0.351015000	-3.229752000

6 4.671451000 -2.227983000 -2.356114000
1 5.165636000 -2.574918000 -3.268237000
6 4.673289000 -3.036214000 -1.213446000
1 5.164285000 -4.013236000 -1.230124000
6 4.032501000 -2.602123000 -0.051095000
6 -3.404880000 -1.352908000 -0.048578000
6 -3.404035000 -0.530501000 -1.177232000
6 -4.037802000 -0.981093000 -2.337634000
1 -4.029632000 -0.350444000 -3.230478000
6 -4.671472000 -2.227117000 -2.356706000
1 -5.165461000 -2.574166000 -3.268904000
6 -4.673840000 -3.035194000 -1.213904000
1 -5.165078000 -4.012093000 -1.230647000
6 -4.033309000 -2.601097000 -0.051450000
1 -4.004749000 -3.227201000 0.844156000
1 -2.906265000 0.438653000 -1.135440000
1 2.906417000 0.438009000 -1.135044000
1 4.003584000 -3.228398000 0.844378000
16 0.000136000 0.209052000 -1.726824000

INT (X=S)

E= -6732.0289292
Free Energy = -6731.768577

32 0.639929000 0.409452000 -1.122104000
31 -2.182365000 -1.312200000 -0.465926000
17 -3.926560000 -1.424886000 0.880709000
7 0.169954000 3.228850000 0.319275000
6 0.931312000 2.360772000 -0.385757000
17 -2.658977000 -0.001996000 -2.227459000
7 2.091900000 3.024681000 -0.629603000
6 2.623754000 0.176060000 -0.439377000
17 -1.628828000 -3.291683000 -1.325939000
7 3.548084000 1.135730000 -0.720564000
6 0.857273000 4.419132000 0.533951000
1 0.403665000 5.247364000 1.072452000
7 3.334063000 -0.841729000 0.108959000
6 2.076805000 4.291582000 -0.057952000
1 2.910632000 4.985184000 -0.139853000
6 3.177065000 2.389570000 -1.350456000
1 2.844248000 2.185324000 -2.383752000
1 4.046803000 3.059062000 -1.365504000
6 4.823888000 0.735765000 -0.344757000
1 5.704104000 1.357263000 -0.493053000
6 4.687075000 -0.514188000 0.172656000
1 5.425115000 -1.210006000 0.562142000
6 -1.138659000 2.940405000 0.841578000
6 -2.143821000 2.517774000 -0.025317000
6 -3.376617000 2.131720000 0.500880000
1 -4.140673000 1.737901000 -0.171451000
6 -3.599537000 2.190145000 1.877996000
1 -4.554281000 1.850078000 2.286756000
6 -2.592975000 2.647879000 2.734480000
1 -2.762308000 2.681127000 3.814401000
6 -1.350030000 3.020179000 2.219599000
6 2.795704000 -2.068435000 0.626617000
6 1.831741000 -2.777824000 -0.090196000
6 1.309279000 -3.954648000 0.449981000
1 0.520833000 -4.474985000 -0.096615000
6 1.757631000 -4.420956000 1.687530000
1 1.338693000 -5.339280000 2.108439000
6 2.727099000 -3.705221000 2.397905000
1 3.068437000 -4.057223000 3.375540000
6 3.244382000 -2.520513000 1.872869000
1 3.969246000 -1.933331000 2.442777000
1 1.470621000 -2.425027000 -1.057400000
1 -0.535261000 3.320441000 2.883959000
1 -1.958904000 2.432584000 -1.096762000
16 -0.424212000 -0.413317000 0.697799000

INT (X=Se)

E = -8735.2467567
Free energy= -8734.988797

32 0.721448000 0.443385000 -1.119838000
31 -2.219048000 -1.307590000 -0.475656000
17 -4.041627000 -1.265068000 0.775327000
7 0.232281000 3.290487000 0.244740000
6 1.016841000 2.399524000 -0.406889000
17 -2.534443000 -0.048375000 -2.309291000
7 2.193126000 3.050409000 -0.612135000
6 2.701448000 0.189623000 -0.450460000
17 -1.765255000 -3.357214000 -1.218143000
7 3.637598000 1.150027000 -0.687115000
6 0.920300000 4.479078000 0.465056000
1 0.450755000 5.322214000 0.965254000
7 3.397522000 -0.852612000 0.071550000
6 2.163065000 4.329625000 -0.069630000
1 3.005770000 5.014729000 -0.128826000
6 3.293845000 2.415076000 -1.307304000

1	2.993113000	2.229690000	-2.354203000
1	4.168490000	3.078107000	-1.285652000
6	4.905086000	0.727085000	-0.307605000
1	5.791930000	1.346181000	-0.422550000
6	4.751375000	-0.537975000	0.166127000
1	5.477846000	-1.252472000	0.543407000
6	-1.106961000	3.036424000	0.700400000
6	-2.060942000	2.571631000	-0.202479000
6	-3.329216000	2.227906000	0.265622000
1	-4.054786000	1.803623000	-0.430197000
6	-3.637705000	2.367589000	1.620174000
1	-4.620202000	2.059102000	1.985761000
6	-2.681013000	2.864866000	2.511129000
1	-2.916865000	2.961230000	3.574669000
6	-1.404123000	3.197506000	2.055535000
6	2.844563000	-2.096147000	0.527828000
6	1.901720000	-2.775653000	-0.244258000
6	1.358563000	-3.969019000	0.235454000
1	0.584266000	-4.467277000	-0.350034000
6	1.764396000	-4.478935000	1.470427000
1	1.327263000	-5.408382000	1.845799000
6	2.712270000	-3.792659000	2.236731000
1	3.019260000	-4.179881000	3.212400000
6	3.251722000	-2.593209000	1.770481000
1	3.959528000	-2.028906000	2.383246000
1	1.574732000	-2.381457000	-1.208229000
1	-0.629508000	3.528337000	2.752545000
1	-1.811683000	2.423853000	-1.254064000
34	-0.389068000	-0.415962000	0.829392000

TS2 (X=S)

E = -6731.9873278
Free Energy = -6731.726586

32	0.217797000	0.777172000	0.122292000
31	-0.596838000	-2.454285000	-0.761203000
17	-2.165316000	-3.848137000	-0.099479000
7	-2.302591000	2.409344000	0.067862000
6	-1.008030000	2.151604000	-0.305826000
17	-1.327467000	-1.178217000	-2.434440000
7	-0.597828000	3.266308000	-1.010191000
6	1.806375000	1.679527000	-0.334604000
17	1.290626000	-3.435933000	-1.367404000
7	1.754897000	2.881841000	-1.023974000
6	-2.673115000	3.679118000	-0.369945000
1	-3.670104000	4.069339000	-0.183343000
7	3.129782000	1.509941000	0.004591000
6	-1.618795000	4.212548000	-1.040385000
1	-1.511586000	5.163360000	-1.557017000
6	0.602089000	3.232678000	-1.813983000
1	0.466697000	2.455125000	-2.601449000
1	0.759883000	4.219195000	-2.270762000
6	3.024290000	3.454353000	-1.074563000
1	3.212892000	4.397465000	-1.582002000
6	3.870518000	2.606343000	-0.437190000
1	4.944338000	2.660110000	-0.279806000
6	-3.134681000	1.511677000	0.803991000
6	-3.287381000	0.197490000	0.352843000
6	-4.062118000	-0.695226000	1.095709000
1	-4.143082000	-1.731813000	0.758416000
6	-4.701377000	-0.265467000	2.262093000
1	-5.307866000	-0.966625000	2.842462000
6	-4.561267000	1.058701000	2.694086000
1	-5.055778000	1.393996000	3.610324000
6	-3.766815000	1.952081000	1.972656000
6	3.655298000	0.390561000	0.709976000
6	3.278315000	-0.900988000	0.329424000
6	3.769844000	-1.998143000	1.037586000
1	3.445305000	-2.997533000	0.737092000
6	4.652372000	-1.808038000	2.104395000
1	5.038543000	-2.669309000	2.656907000
6	5.038761000	-0.512979000	2.469915000
1	5.722892000	-0.359335000	3.309487000
6	4.534736000	0.592265000	1.782056000
1	4.802764000	1.607317000	2.087000000
1	2.612502000	-1.067775000	-0.518614000
1	-3.617845000	2.977168000	2.322541000
1	-2.808236000	-0.124161000	-0.574656000
16	-0.145381000	-1.103436000	1.097028000

TS2 (X=Se)

E= -8735.2066002
Free energy = -8734.947858

32	-0.254042000	0.876271000	0.128761000
31	0.648299000	-2.392663000	-0.885313000
17	2.266985000	-3.781785000	-0.332508000
7	-3.181726000	1.532584000	-0.032942000
6	-1.858754000	1.748573000	-0.343301000
17	-1.228527000	-3.388743000	-1.509557000
7	-1.833859000	2.956755000	-1.023259000
6	0.942190000	2.277909000	-0.310650000
17	1.310202000	-1.021950000	-2.512924000
7	0.510893000	3.389861000	-1.005951000
6	-3.951179000	2.605027000	-0.484644000
1	-5.028900000	2.621677000	-0.346708000
7	2.240322000	2.546018000	0.040090000
6	-3.121526000	3.484480000	-1.100414000

1	-3.331289000	4.424407000	-1.605483000
6	-0.689007000	3.342395000	-1.809237000
1	-0.538896000	2.582406000	-2.610906000
1	-0.868076000	4.332662000	-2.249859000
6	1.525150000	4.342871000	-1.056913000
1	1.400776000	5.293400000	-1.570234000
6	2.594761000	3.818021000	-0.404424000
1	3.591373000	4.215949000	-0.232575000
6	-3.689341000	0.380470000	0.633411000
6	-3.282511000	-0.890900000	0.217065000
6	-3.764194000	-2.019270000	0.881885000
1	-3.417112000	-3.002435000	0.554293000
6	-4.663935000	-1.879609000	1.941973000
1	-5.041906000	-2.765312000	2.460513000
6	-5.077956000	-0.604302000	2.344884000
1	-5.775562000	-0.490520000	3.179690000
6	-4.585587000	0.531486000	1.699596000
6	3.101205000	1.644844000	0.739868000
6	3.289058000	0.354032000	0.237638000
6	4.095888000	-0.542712000	0.940603000
1	4.205674000	-1.563090000	0.563898000
6	4.728169000	-0.139471000	2.120062000
1	5.358857000	-0.843948000	2.669850000
6	4.549748000	1.161640000	2.605068000
1	5.038697000	1.475336000	3.531863000
6	3.725547000	2.058360000	1.922032000
1	3.548530000	3.064877000	2.310645000
1	2.807799000	0.051931000	-0.695482000
1	-4.876914000	1.531521000	2.031646000
1	-2.599980000	-1.017431000	-0.624914000
34	0.184589000	-1.103735000	1.161775000

Compound E

E = -9133.386066
Free energy = -9133.126398

32	-1.203175000	0.127817000	0.000000000
31	2.329272000	-0.621668000	0.000000000
17	2.534834000	0.774842000	1.765211000
6	-0.556959000	1.409957000	1.417174000
17	3.946941000	-2.093732000	0.000000000
6	-0.298959000	2.423932000	3.399213000
1	-0.340191000	2.510182000	4.481595000
6	0.123558000	3.292504000	2.439039000
1	0.546204000	4.291362000	2.514165000
6	0.583294000	3.117209000	0.000000000
1	0.568897000	4.216411000	0.000000000
1	1.625105000	2.749009000	0.000000000
6	-1.149722000	0.056548000	3.377643000
6	-0.168436000	-0.841822000	3.802823000
6	-1.930136000	-2.333158000	4.533803000
1	-2.239599000	-3.280509000	4.984774000
7	-0.062763000	2.654913000	1.217860000
6	-2.897553000	-1.429023000	4.084600000
1	-3.960406000	-1.668914000	4.173407000
6	-2.511320000	-0.221834000	3.497247000
7	-0.723446000	1.279956000	2.744247000
34	0.205031000	-1.746991000	0.000000000
6	-0.569154000	-2.042520000	4.391444000
1	0.186423000	-2.760845000	4.720499000
17	2.534834000	0.774842000	-1.765211000
6	-0.556959000	1.409957000	-1.417174000
6	-0.298959000	2.423932000	-3.399213000
1	-0.340191000	2.510182000	-4.481595000
6	0.123558000	3.292504000	-2.439039000
1	0.546204000	4.291362000	-2.514165000
6	-1.149722000	0.056548000	-3.377643000
6	-0.168436000	-0.841822000	-3.802823000
6	-1.930136000	-2.333158000	-4.533803000
1	-2.239599000	-3.280509000	-4.984774000
7	-0.062763000	2.654913000	-1.217860000
6	-2.897553000	-1.429023000	-4.084600000
1	-3.960406000	-1.668914000	-4.173407000
6	-2.511320000	-0.221834000	-3.497247000
7	-0.723446000	1.279956000	-2.744247000
6	-0.569154000	-2.042520000	-4.391444000
1	0.186423000	-2.760845000	-4.720499000
1	-3.247999000	0.476152000	-3.097518000
1	0.885168000	-0.605684000	-3.638072000
1	-3.247999000	0.476152000	3.097518000
1	0.885168000	-0.605684000	3.638072000
16	-3.300067000	0.085794000	0.000000000

Compound F

E = -9133.3889204
Free energy = -9133.127578

34	-3.086464000	-0.119711000	0.000000000
32	-0.863844000	-0.016619000	0.000000000
31	2.561424000	-0.766035000	0.000000000
17	2.781463000	0.608595000	1.775243000
6	-0.268568000	1.290969000	1.420799000
17	4.128863000	-2.286369000	0.000000000
6	-0.166187000	2.381820000	3.377580000
1	-0.277866000	2.503820000	4.451570000
6	0.305960000	3.223911000	2.416473000
1	0.703337000	4.233763000	2.482768000

6	0.893592000	2.969606000	0.000000000
1	0.932471000	4.067592000	0.000000000
1	1.916298000	2.552184000	0.000000000
6	-1.011177000	0.012029000	3.378465000
6	-0.133238000	-1.060255000	3.546013000
6	-1.948404000	-2.319264000	4.538995000
1	-2.321377000	-3.243149000	4.990479000
7	0.216363000	2.538339000	1.211357000
6	-2.814391000	-1.235103000	4.359418000
1	-3.862937000	-1.311948000	4.659185000
6	-2.349496000	-0.059834000	3.767441000
7	-0.521316000	1.205194000	2.738414000
6	-0.613984000	-2.233872000	4.131058000
1	0.058671000	-3.086170000	4.257714000
17	2.781463000	0.608595000	-1.775243000
6	-0.268568000	1.290969000	-1.420799000
6	-0.166187000	2.381820000	-3.377580000
1	-0.277866000	2.503820000	-4.451570000
6	0.305960000	3.223911000	-2.416473000
1	0.703337000	4.233763000	-2.482768000
6	-1.011177000	0.012029000	-3.378465000
6	-0.133238000	-1.060255000	-3.546013000
6	-1.948404000	-2.319264000	-4.538995000
1	-2.321377000	-3.243149000	-4.990479000
7	0.216363000	2.538339000	-1.211357000
6	-2.814391000	-1.235103000	-4.359418000
1	-3.862937000	-1.311948000	-4.659185000
6	-2.349496000	-0.059834000	-3.767441000
7	-0.521316000	1.205194000	-2.738414000
6	-0.613984000	-2.233872000	-4.131058000
1	0.058671000	-3.086170000	-4.257714000
1	-3.019052000	0.780770000	-3.575574000
1	0.900515000	-0.968865000	-3.207381000
1	-3.019052000	0.780770000	3.575574000
1	0.900515000	-0.968865000	3.207381000
16	0.506783000	-1.758942000	0.000000000

S_8

E= -3184.7327859
Free energy = -3184.759692

16	-2.208780000	0.914878000	-0.508956000
16	0.914873000	2.208815000	-0.508954000
16	2.208780000	-0.914878000	-0.508956000
16	-0.914873000	-2.208815000	-0.508954000
16	-0.914873000	2.208626000	0.508964000
16	2.208603000	0.914875000	0.508946000
16	-2.208603000	-0.914875000	0.508946000
16	0.914873000	-2.208626000	0.508964000

Se_8

E = -19210.5504065
Free energy = -19210.59163

34	0.000107000	2.672478000	0.587731000
34	-2.672172000	0.000094000	0.587963000
34	-0.000107000	-2.672478000	0.587731000
34	2.672172000	-0.000094000	0.587963000
34	-1.889116000	1.889212000	-0.587784000
34	-1.889116000	-1.889002000	-0.587909000
34	1.889116000	1.889002000	-0.587909000
34	1.889116000	-1.889212000	-0.587784000

D. References

1. Y. Xiong, S. Yao, G. Tan, S. Inoue, and M. Driess, *J. Am. Chem. Soc.* 2013, **135**, 5004.
2. G. M. Sheldrick, *SHELX-97 Program for Crystal Structure Determination*, Universität Göttingen (Germany) **1997**.
3 Gaussian 09, Revision D.01, M. J. Frisch, G. W. Trucks, H. B. Schlegel, G. E. Scuseria, M. A. Robb, J. R. Cheeseman, G. Scalmani, V. Barone, B. Mennucci, G. A. Petersson, H. Nakatsuji, M. Caricato, X. Li, H. P. Hratchian, A. F. Izmaylov, J. Bloino, G. Zheng, J. L. Sonnenberg, M. Hada, M. Ehara, K. Toyota, R. Fukuda, J. Hasegawa, M. Ishida, T. Nakajima, Y. Honda, O. Kitao, H. Nakai, T. Vreven, J. A. Montgomery, Jr., J. E. Peralta, F. Ogliaro, M. Bearpark, J. J. Heyd, E. Brothers, K. N. Kudin, V. N. Staroverov, T. Keith, R. Kobayashi, J. Normand, K. Raghavachari, A. Rendell, J. C. Burant, S. S. Iyengar, J. Tomasi, M. Cossi, N. Rega, J. M. Millam, M. Klene, J. E. Knox, J. B. Cross, V. Bakken, C. Adamo, J. Jaramillo, R. Gomperts, R. E. Stratmann, O. Yazyev, A. J. Austin, R. Cammi, C. Pomelli, J. W. Ochterski, R. L. Martin, K. Morokuma, V. G. Zakrzewski, G. A. Voth, P. Salvador, J. J. Dannenberg, S. Dapprich, A. D. Daniels, O. Farkas, J. B. Foresman, J. V. Ortiz, J. Cioslowski, and D. J. Fox, Gaussian, Inc., Wallingford CT, 2013.
4 F. Neese, *ORCA, version 3.0.2, An ab initio, density functional and semiempirical program package,* Max Planck Institute for Bioinorganic Chemistry, Mülheim a.d. Ruhr, Germany, **2012**.
5 (a) A. D. Becke, *Phys. Rev. A* 1988, **38**, 3098; (b) C. T. Lee, W. T. Yang, R. G. Parr, *Phys. Rev. B* 1988, **37**, 785; (c) A.D. Becke, *J. Phys. Chem*. 1993, **98**, 5648.
6 (a) A. Schaefer, C. Huber, and R. Ahlrichs, *J. Chem. Phys*., 1994, **100** 5829; (b) F. Weigend, *Phys. Chem. Chem. Phys.*, 2006, **8**, 1057.
7 0th order regular approximation. E. van Lenthe, E. J. Baerends, and J. G. Snijders, *J. Chem. Phys*. 1993, **99** 4597.
8 J. P. Perdew, *Phys. Rev. B*, 1986, **33**, 8822.
9 S. Grimme, J. Antony, S. Ehrlich, H. Krieg, *J. Chem. Phys*., 2010, **132**, 154104.
10 S. Grimme, S. Ehrlich and L. Goerigk, *J. Comp. Chem*. 2011, **32** 1456.
11 J. Tomasi, B. Mennucci, and R. Cammi, *Chem. Rev.*, 2005, **105** 2999.
12 NBO 6.0. E. D. Glendening, J. K. Badenhoop, A. E. Reed, J. E. Carpenter, J. A. Bohmann, C. M. Morales, C. R. Landis, and F. Weinhold, Theoretical Chemistry Institute, University of Wisconsin, Madison (2013).

13 A. E. Reed, R. B. Weinstock, and F. Weinhold, *J. Chem. Phys.* 1985, **83**, 735.
14 K. B. Wiberg, *Tetrahedron*, 1968, **24**, 1083.
15 (a) E. D. Glendening and F. Weinhold, *J. Comp. Chem*. 1998, **19**, 593; (b) E. D. Glendening and F. Weinhold, J. Comp. Chem. 1998, **19**, 610; (c) E. D. Glendening, J. K. Badenhoop, and F. Weinhold, *J. Comp. Chem*. 1998, **19**, 628.
16 W. Kutzelnigg, U. Fleischer, M. Schindler. *The IGLO-Method: Ab Initio Calculation and Interpretation of NMR Chemical Shifts and Magnetic Susceptibilities*, volume 23. Springer Verlag, 1990.
17 (a) C. J. Jameson, in *Specialist Periodic Reports on NMR*, edited by G. A. Webb (Royal Society of Chemistry, London, 1994), Vol. 24. P. 1. (b) G. Schreckenbach, Y. Ruiz-Morales, T. Ziegler, *J.Chem. Phys*, 1996, **104**, 8605; (c)W. Gombler, *Phosphorus and Sulfur*, 1988, Vol. **38**, pp. 231-243; (d) E. R. Cullen, F. S. Guziec, Jr., C. J. Murphy, T. C. Wong, K. K. Andersen, *J. Am. Chem. SOC.* 1981, **103**, 7055; (e) W. Nakanishi, S. Hayashi, *J. Phys. Chem. A* 1999, **103**, 6074; (f) J. Peralta-Cruz, V. I. Bakhmutov, A. Ariza-Castolo, *Magn. Reson. Chem*. 2001, **39**, 187; (g) Y. Yu. Rusakov, L. B. Krivdin, *J. Comput. Chem.* 2015, **36**, 1756; (h) T. W. Keal, D. J. Tozer, *Molecular Physics*, 2005, **103**, 1007; (i) B. Wrackmeyer, Z. G. Hernandez, M. Herberhold, *Magn. Reson. Chem*. 2007, **45**, 198; (j) W. McFarlane, D.S. Rycroft, *J. Chem. Soc. Chem. Comm.*, 1972, 902.
18 (a) R. Boese, A. Hass, C. Limberg, *J. Chem. Soc.,Chem. Commun*. **1991**, 1378; (b) Y. Ruiz-Morales, G. Schreckenbach, T. Ziegler, *J. Phys. Chem. A* 1997, **101**, 4121.
